# Encapsulated Non‐Exchangeable Na^+^ Ions Determining the Upper Limit of Al Inclusion in FAU—A Multiscale Simulation

**DOI:** 10.1002/anie.202524044

**Published:** 2026-02-03

**Authors:** Qi Dong, Tao Zhang, Chuanhao Zhang, Tong Zhang, Yanze Du, Jinghong Ma, Ruifeng Li, Bo Qin, Haijun Jiao

**Affiliations:** ^1^ State Key Laboratory of Clean and Efficient Coal Utilization College of Chemistry and Chemical Engineering Taiyuan University of Technology Taiyuan China; ^2^ Leibniz‐Institut für Katalyse e.V. Rostock Germany; ^3^ School of Chemistry and Life Resources Renmin University of China Beijing China; ^4^ SINOPEC Dalian Research Institute of Petroleum & Petrochemicals Co., Ltd Dalian China

**Keywords:** acid strength, Al distribution, non‐exchangeable Na^+^ ions, Si/Al ratio, Y‐zeolite

## Abstract

Na^+^ ion as charge‐balance agent controlling the distribution of framework aluminum atoms in Y‐zeolite from 1 to 14 Al atoms (Si/Al = 47‐2.4) and the acid strength have been investigated based on DFT computations, descriptors from machine learning, AIMD simulations, and experimental analysis. Different from previous results with proton (H^+^) as charge‐balancing agent preferring next‐nearest‐neighbor Al separation (3N‐Al), the framework Al atoms, in the presence of Na^+^ ions, prefer larger separations (4N‐Al and 5N‐Al) due to the balance between Na^+^/AlO_4_^−^ attractive and AlO_4_^−^/AlO_4_^−^ repulsive interaction and the preferential occupancy of Na^+^ ions following the order of six‐membered ring (6MR) > double six‐membered ring (D6R) > four‐membered ring (4MR). The computed sequential substitution enthalpy for 1‐14 Al shows the thermodynamically favorable upper limit of rather low Si/Al ratio (≤ 3) and explains the difficult synthesis of Y‐zeolite with higher Si/Al ratios. Y‐zeolite with non‐exchangeable Na^+^ ions has stronger acid strength based on the adsorption energy of pyridine and ammonia and exhibits higher catalytic activity in propane cracking.

## Graphical abstract

DFT computation, AIMD simulations, machine learning, statistical analysis, and experimental characterization reveal that the upper limit of Al inclusion in FAU zeolite synthesized under hydrothermal conditions is determined by the encapsulated non‐exchangeable Na^+^ ions. The balanced repulsive‐attractive electrostatic interaction among Na^+^ ions and multiple AlO_4_^−^ units is the driving force to control the location and distribution of Al inclusion. This affects the acid strength and catalytic activity.

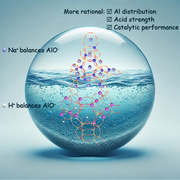

## 1 Introduction

Zeolites, characterized by their well‐defined microporous frameworks, tunable acidity, high thermal or hydrothermal stability, and large specific surface areas, have found wide applications in heterogeneous catalysis and materials sciences [1, 2, 3, 4]. Since the first natural zeolite was identified in 1756, over 250 distinct framework types, encompassing natural and synthetic materials, have been catalogued by the International Zeolite Association. One industrially important example is the Faujasite type zeolite (FAU), synthesized under hydrothermal conditions at Union Carbide in 1954, having a three‐dimensional pore structure, consisting of roughly 1.2 nm super‐cages and 0.8 nm windows interconnected via sodalite cages (β cages) and double six‐membered rings (D6Rs). This makes it suitable for applications in catalytic cracking, hydrocracking, biomass conversion, adsorption, and separation [5, 6, 7, 8].

For Y‐zeolites as catalysts, the Si/Al ratio and spatial distribution of framework Al atoms have been recognized as the intrinsic factors governing the Brønsted acid strength, hydrothermal stability, and catalytic activity. However, conventionally synthesized Y‐zeolites from inorganic systems typically exhibit high Al contents with Si/Al ratios rarely exceeding 3 (Si/Al > 3) [9], while the spatial distribution of framework Al atoms has long been intensely debated [10]. Consequently, dealumination is a widely used post‐synthetic strategy to increase the Si/Al ratio of Y‐zeolites. However, this approach is inherently costly and suffers from significant drawbacks, including partial loss of crystallinity, high energy and labor demands, and the inevitable formation of amorphous by‐products. These drawbacks have stimulated extensive efforts toward the direct in situ synthesis of Y‐zeolites with high Si/Al ratios, using inorganic structure‐directing agents (ISDAs) [10, 11, 12], organic structure‐directing agents (OSDAs) [13, 14, 15, 16], and zeolite seeds [17, 18]. All these methods point out that the high amounts of alkali metal (Na^+^ ions) in reactant gels might hinder the synthesis of Y‐zeolite with a high Si/Al ratio (> 3). The spatial distribution of framework Al atoms exerts an equal decisive influence as the overall Si/Al ratio, since the binding environments of charge‐compensating cations determine the location and strength of Brønsted acid sites and thereby govern catalytic activity and selectivity [19, 20, 21, 22]. Considerable efforts have then been devoted to exploring Al siting through advanced spectroscopic techniques, probing molecular experimental and theoretical approaches [10, 23, 24, 25, 26]. For example, solid‐state ^29^Si NMR spectroscopy has been used to analyze Si(nAl) distributions and Al‐Al correlations in FAU‐type zeolites, revealing nonrandom distributions of framework Al atoms [27]. Nevertheless, these approaches provide only limited insight into the intrinsic factors governing the Si/Al ratio and Al distribution. It remains difficult to distinguish the effects of bulk composition (e.g., Si/Al ratio) from local Al configurations, mainly between isolated and adjacent sites. This ambiguity fueled the ongoing debate about how site proximity affects acid strength and catalytic behavior.

Theoretical studies have been employed to complement atomic‐level studies; however, the vast configurational space associated with framework heterogeneity remains difficult to resolve. Bridging atomistic simulations with experimental observables often requires a statistical thermodynamic treatment, where Boltzmann weighting provides a rigorous basis for deriving equilibrium populations and energy‐averaged structural descriptors, enabling direct comparison with experiments [28, 29]. Recently, machine learning has emerged as a powerful complement, offering accelerated property predictions and systematic identification [30, 31, 32] of local structural environments and key descriptors governing framework stability and reactivity [33, 34, 35]. However, it should be noted that previous theoretical studies on Y‐zeolites have adopted protonic forms (H‐Y), in which framework AlO_4_^−^ units are directly charge‐compensated by protons (H^+^) [36]. Based on the thermodynamic stability of such models, it has been commonly concluded that framework Al atoms tend to adopt regular spatial distributions and preferentially occupy next‐nearest‐neighbor (3N‐Al) separations across four‐membered rings [36, 37, 38, 39]. Considering that FAU crystallization typically proceeds under alkaline hydrothermal conditions, the role of these alkali species in determining Al siting should not be overlooked [40, 41, 42, 43]. In 1966, Sherry et al. [44], found that within the Li^+^, K^+^, Rb^+^, Cs^+^, Ag^+^, and NH_4_^+^ ion‐exchange experiments, about 16 of the original Na^+^ ions per unit cell in Na‐Y cannot be exchanged. These show that current theoretical models of H‐Y‐zeolite are oversimplified and ignore the role of alkaline ions in determining the Si/Al ratio, the distribution of framework Al atoms, and the acid properties, thereby biasing our understanding of Y‐zeolite's properties.

Here, we present an integrated computational‐experimental method to elucidate the stability and structural principle governing Al distributions in Na‐Y zeolites over a broad compositional range (Si/Al = 47‐2.4, *n*_Al_ = 1–14). Reasonable structural models were constructed by incorporating the positional preference of Na^+^ ions under synthetic alkaline conditions, followed by systematic density functional theory (DFT) computations and ab initio molecular dynamics (AIMD) simulations. To capture configurational thermodynamics beyond discrete structural snapshots, Boltzmann‐weighted statistics were employed, revealing a preference for spatially separated Al sites, driven by the competitive electrostatic attraction (Na^+^‐AlO_4_^−^) and framework charge repulsion, resulting in Al‐Al separations different from the next‐nearest‐neighbor (3N‐Al) separation. In addition, the acid strength of these models has been evaluated via the adsorption of pyridine and ammonia, as well as the catalytic cracking of propane as a model substrate. By combining computational and experimental results, the detailed structures of weak acid, medium acid, and strong acid sites in NH_3_‐TPD were revealed and verified.

This comprehensive multiscale approach, spanning first‐principles calculations, statistical thermodynamics, machine learning classification, and experimental verification, provides fundamental insight into the interplay between Al distributions, charge‐compensating cation (Na^+^) positions, and acid strength in FAU‐type zeolites. The predicted Al distributions agree with those reported from structural characterizations, thereby validating the predictive model and its identification of key factors controlling Al distribution. That the synthesized Y‐zeolites with rather lower Si/Al ratio (≤3) are due to the intrinsic nature of the balanced attractive interaction between Na^+^ ions and AlO_4_^−^ units and the repulsive interaction among the AlO_4_^−^ units. These findings not only reconcile theoretical predictions with experimentally accessible observables but also establish a robust platform for the rational design of framework compositions and acid site topologies in zeolite catalysis.

## 2 Results and Discussion

### 2.1 Na^+^ Ions Preferred Locations

**FIGURE 1:**
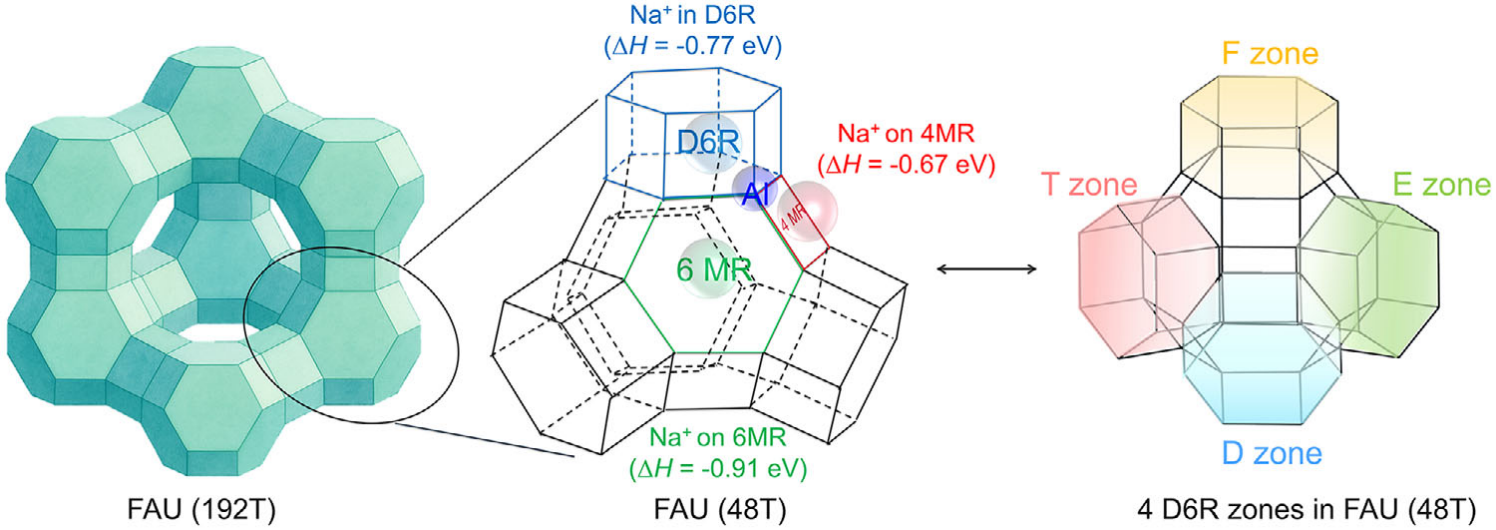
FAU structure (48T) and substitution enthalpy for 1Al substitution with Na^+^ ion on 6MR, 4MR, and D6R, and the labeled E, F, D, and T zones.

Given that the distribution of framework Al atoms in zeolite is predetermined during the synthesis under the influence of a Na^+^‐containing environment, elucidating the nature and location of Na^+^ ions becomes essential. Since FAU‐type zeolite has only one equivalent tetrahedral site, a simple Na‐Y model comprising a single framework Al atom charge‐balanced by one Na^+^ cation can be easily constructed. The computed substitution enthalpy of this model shows that the Na^+^ ion, compensating for the negative charge of the AlO_4_^−^, resides most preferentially at the six‐membered ring (6MR, ‐0.91 eV), followed by the double six‐membered ring (D6R, ‐0.77 eV) and least preferentially at the four‐membered ring (4MR, ‐0.67 eV). (Figure 1 and Table S2). Compared to the most stable structure with Na^+^ ion at the 6MR, the lower stability of Na^+^ ion at 4MR shows not only shorter average distances of Na^+^ ion and AlO_4_^−^ unit (Na‐O: 2.38 vs. 2.23 Å) but also the lower coordination number of Na^+^ ion to the surrounding framework oxygen atoms (4 vs. 2). Based on these energetic differences, the expected population of the most stable Na^+^ ion location at the 6MR should be as high as over 99%, while those at D6R and 4MR should not be negligible, indicating the preferred location of Na^+^ ions.

### 2.2 Al Distribution

Based on the most stable configuration of one Al atom in the framework and the Löwenstein's rule, we explored further Al substitution by gradually replacing Si with Al balanced by the corresponding number of Na^+^ ions to locate all possible Al sites within a series of 48T periodic models of zeolite Y, where all potential locations of Na^+^ ions (D6R, 6MR and 4MR) were considered. Since the 48T model has four interconnected D6R units, we labeled them as E, F, D, and T zones for simplicity (Figures 1 and S2).

For two Al substitutions, for example, the second Al was put in different separations from the first Al atom, Al‐(O‐Si)_x_‐OAl, that is, the next‐nearest neighbor of Al pairs (*x* = 1, 3N‐Al), the next‐next‐nearest‐neighbor Al pairs (*x* = 2, 4N‐Al), the next‐next‐next‐nearest‐neighbor Al pairs (*x* = 3, 5N‐Al) and the next‐next‐next‐next‐nearest‐neighbor Al pairs (*x* = 4, 6N‐Al) along with all possible locations of Na^+^ ions, giving totally 126 configurations (Table S8).

**FIGURE 2:**
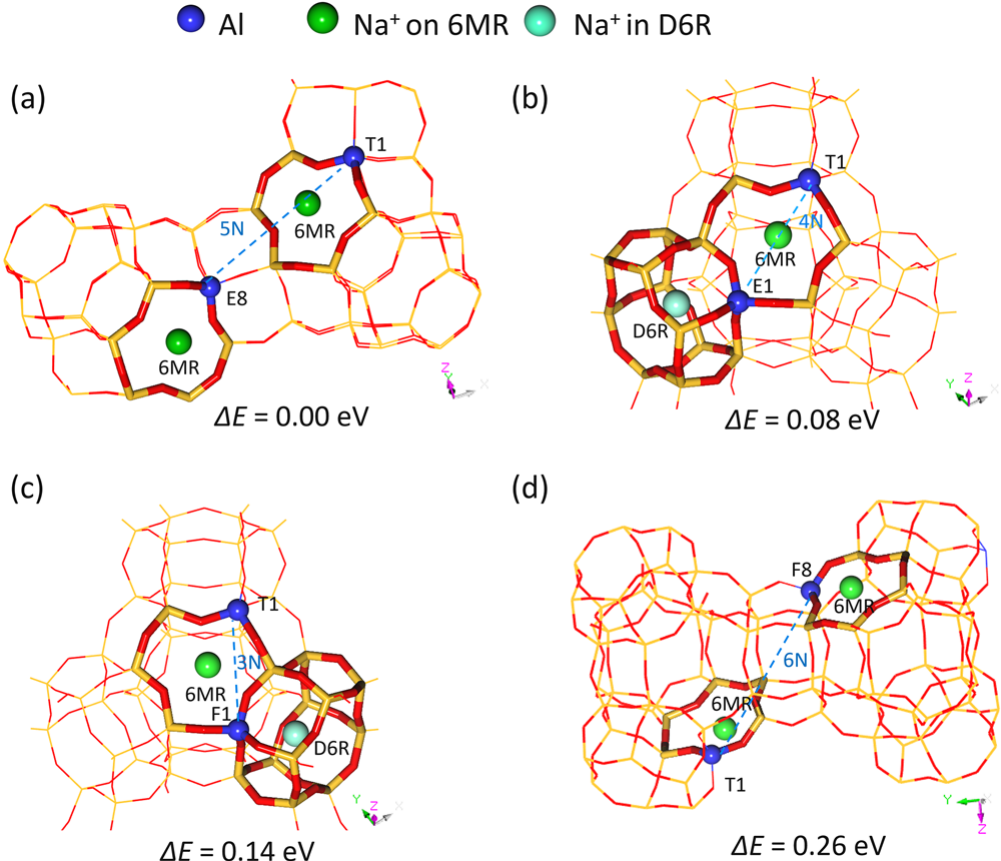
Distribution of two Na^+^ ions and two Al atoms: (a) E8‐6MR (T+E/5N‐Al/6MR+6MR). (b) E1‐D6R (T+E/4N‐Al/6MR+D6R). (c) F1‐D6R (T+F/3N‐Al/6MR+D6R). (d) F8‐6MR (T+F/6N‐Al/6MR+6MR).

Within 0.20 eV, there are nine stable configurations (Table S8). As shown in Figure 2, the most stable configuration E8‐6MR has one Al in the T zone and one Al in the E zone in 5N‐Al separation, as well as two Na^+^ ions located at the 6MR (T1+E8/5N‐Al/6MR+6MR), where each Na^+^ ion interacts with one AlO_4_^−^ unit. The second stable configuration E1‐D6R (0.08 eV) has one Al in T zone and one Al in E zone in 4N‐Al separation as well as the first Na^+^ ion located at the 6MR interacting with two AlO_4_^−^ units, and the second Na^+^ ion located at the D6R interacting with one AlO_4_^−^ unit (T1+E1/4N‐Al/6MR+D6R). The third stable configuration, F1‐D6R (0.14 eV), has one Al in the T zone and one Al in the F zone in 3N‐Al separation, as well as the first Na^+^ ion at the 6MR interacting with two AlO_4_^−^ units and the second Na^+^ ion at the D6R interacting with one AlO_4_^−^ unit (T1+F1/3N‐Al/6MR+D6R).

In H‐Y models, two Al atoms located diagonally across the same 4MR (3N‐Al) are computed to represent the most stable configuration [36], while in our Na‐Y model, the 4MR‐diagonal configuration of two Al atoms (T3‐D6R, Table S8) is much less stable by 0.46 eV than the 5N‐separated configuration (E8‐6MR, Table S8).

In another case, the configuration F8‐6MR (Figure 2d) with the largest 6N‐Al separation and two Na^+^ ions at the 6MR (T1+F8/6N‐Al/6MR+6MR) is much less stable by 0.26 eV (Table S8). Based on these energetic differences, the expected population of the most stable configuration should be as high as over 95%, and those of the second one by 4%, and the third most stable one by much less than 1%. The energetic preference shows that not only the attractive interaction between Na^+^ ions and AlO_4_^−^ units and the repulsive interaction between AlO_4_^−^ units, but also the coordination number of the Na^+^ ions to the surrounding framework oxygen atoms, should determine the stability. In addition, the configuration of 2Al atoms at the same D6R with one Na^+^ ion located at 6MR and one Na^+^ ion at D6R (T+T/5N‐Al/6MR+D6R) is found to be less stable (0.16 eV, Table S8). Nevertheless, we took the most stable 2‐Al configuration (E8‐6MR in Table S8) as the starting point for further Al substitution to capture more possibilities.

**FIGURE 3:**
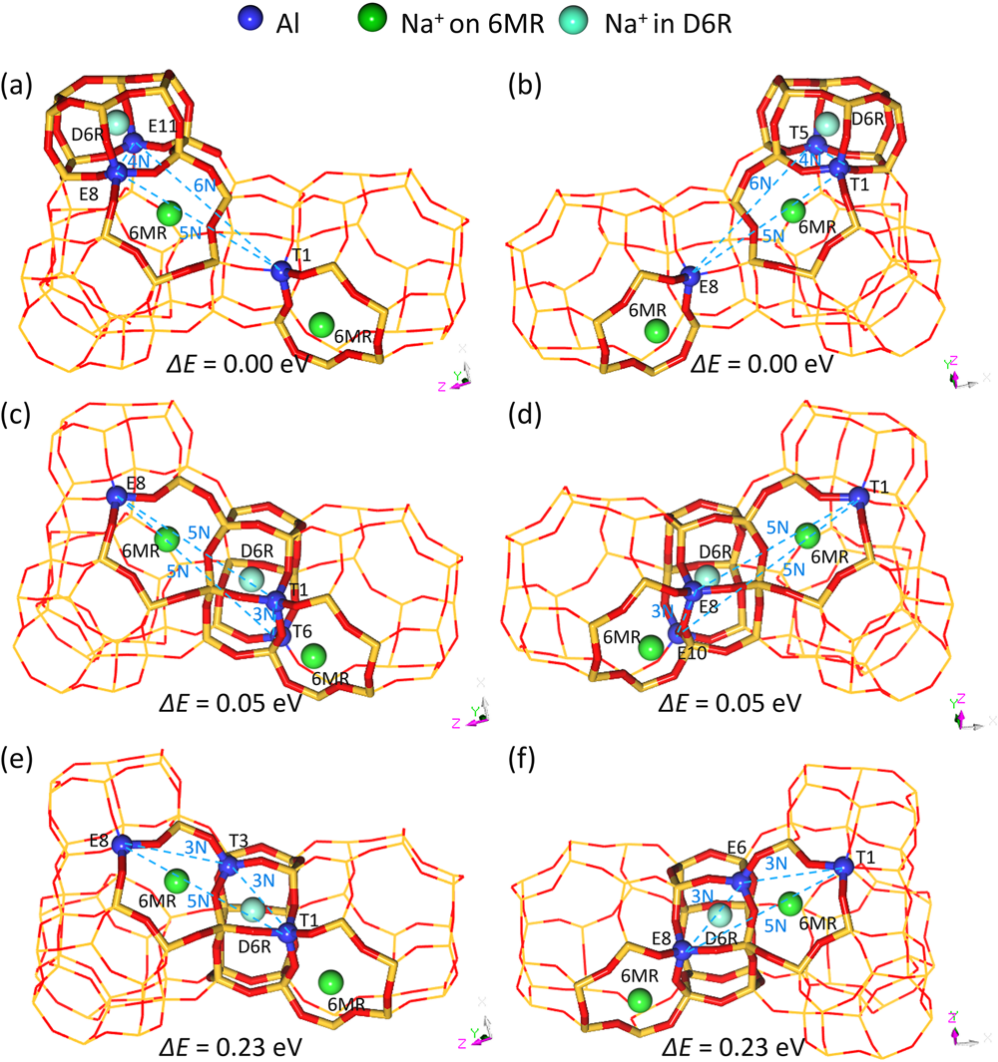
Distribution of three Na^+^ ions and three Al atoms: (a) E11‐D6R (T+E+E/5N+4N+6N‐Al/6MR+6MR+D6R). (b) T5‐D6R (T+E+T/5N+4N+6N‐Al/6MR+6MR+D6R). (c) T6‐D6R (T+E+T/5N+3N+5N‐Al/6MR+6MR+D6R). (d) E10‐D6R (T+E+E/5N+3N+5N‐Al/6MR+6MR+D6R). (e) T3‐D6R (T+E+T/5N+3N+3N‐Al/6MR+6MR+D6R). (f) E6‐D6R (T+E+E/5N+3N+3N‐Al/6MR+6MR+D6R).

Based on the energetic and geometric results for 2Al substitution, we further computed the structures and stability of 3Al substitution with three Na^+^ ions, and there are 110 configurations (Table S9). There are four configurations close in energy (0.05 eV), and the next two are higher in energy by 0.23 eV. The first two most stable degenerated configurations (T5‐D6R and E11‐D6R) have three Al atoms at T+E+T in 5N‐Al, 4N‐Al and 6N‐Al separations with Na^+^ ions at 6MR, 6MR and D6R (T+E+T/5N+4N+6N‐Al/6MR+6MR+D6R) as well as T+E+E in 5N‐Al, 4N‐Al and 6N‐Al separations with Na^+^ ions at 6MR, 6MR and D6R (T+E+E/5N+4N+6N‐Al/6MR+6MR+D6R) (Figure 3a,b). The second two most stable degenerated configurations (T6‐D6R and E10‐D6R) have three Al atoms at T+E+T in 5N‐Al, 3N‐Al and 5N‐Al separations with Na^+^ ions at 6MR, 6MR and D6R (T+E+T/5N+3N+5N‐Al/6MR+6MR+D6R) as well as T+E+E in 5N‐Al, 3N‐Al and 5N‐Al separations with Na^+^ ions at 6MR, 6MR and D6R (T+E+E/5N+3N+5N‐Al/6MR+6MR+D6R) (Figure 3c,d). The third two most stable degenerated configurations (T3‐D6R and E6‐D6R) in the same energy (0.23 eV) have three Al atoms at T+E+T in 5N‐Al, 3N‐Al and 3N‐Al separations with Na^+^ ions at 6MR, 6MR and D6R (T+E+T/5N+3N+3N‐Al/6MR+6MR+D6R) as well as T+E+E in 5N‐Al, 3N‐Al and 3N‐Al separations with Na^+^ ions at 6MR, 6MR and D6R (T+E+E/5N+3N+3N‐Al/6MR+6MR+D6R) (Figure 3e,f). This indicates once again that not only the attractive interaction between Na^+^ ions and AlO_4_^−^ units, but also the repulsive interaction among AlO_4_^−^ units, as well as the coordination number of Na^+^ ions to the surrounding framework oxygen atoms, determine the stability. These energy differences show that the first and second most stable configurations represent the most populated by over 99%, and the third one should be rather negligible.

### 2.3 Statistical Analysis Based on Machine Learning (ML)

**FIGURE 4:**
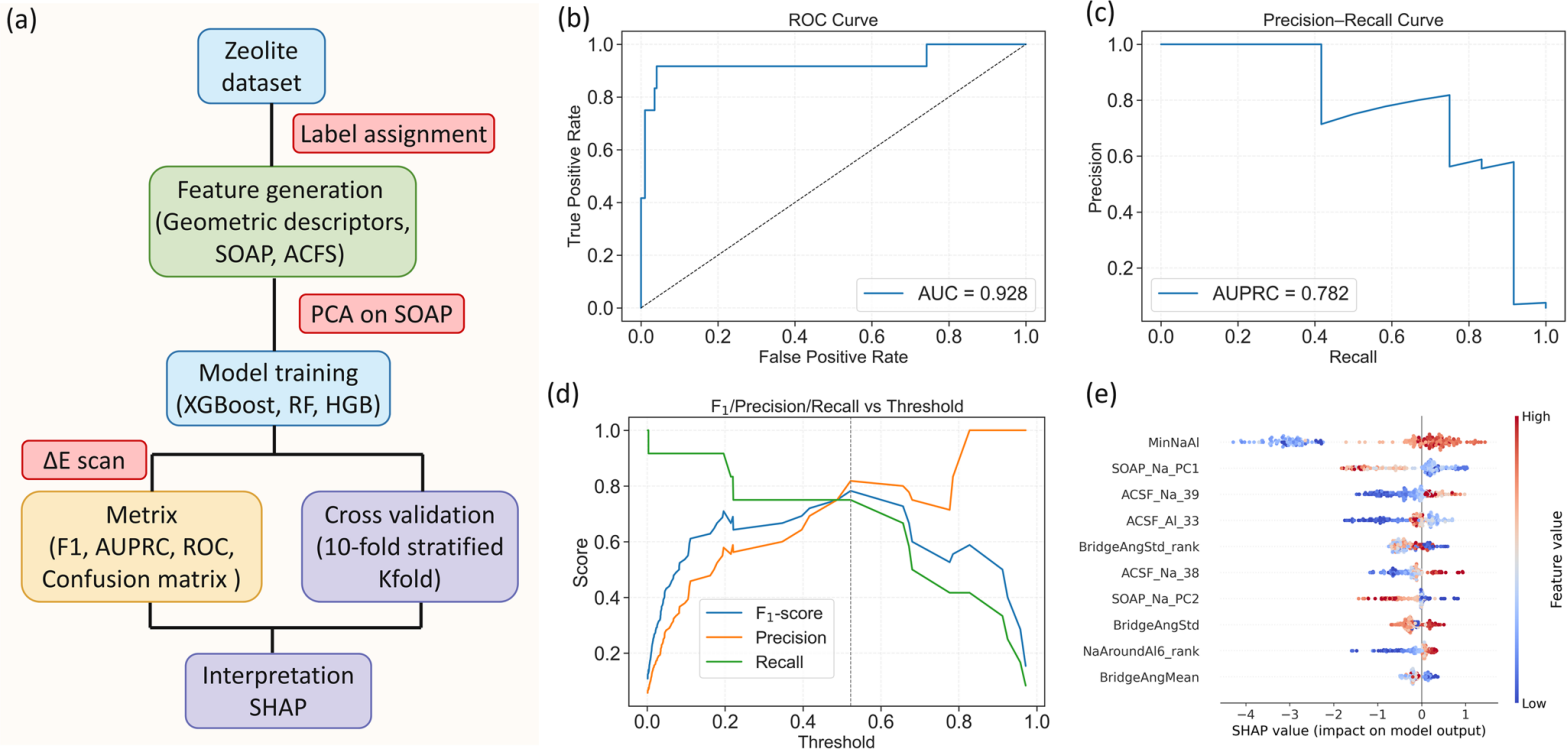
(a) The Machine Learning process. (b) Receiver operating characteristic (ROC) curve (AUC = 0.928). (c) Precision‐recall (PR) curve (AUPRC = 0.782). (d) Threshold‐dependent precision, recall, and F_1_‐score, with the optimal threshold. (e) SHAP summary plot of the top 10 descriptors contributing to the classification model.

Based on these results for 1Al to 3Al substitutions (Tables S3, S7–S9), we further constructed models with 4Al to 14Al substitutions (Tables S10–S20), and all 1288 constructed configurations were fully optimized without any constraints. In these models, charge‐compensating Na^+^ ions were systematically placed at 6MR, D6R, and 4MR. To ensure statistical robustness for classification and to avoid underfitting, we divided data groups by Al atom number and selected those containing over 80 configurations of 1048 samples for further analysis. To include as many stable configurations as possible in training Machine‐Learning in each data group (Figure 4a), we used the energy threshold at 0.25 eV, which covers around 99.994% (298 K) of all low‐level energy structures, to enable a quantitative assessment of the relationship between framework Al distributions and structural stability.

Model performance was rigorously assessed through a 10‐fold stratified cross‐validation using an ensemble voting classifier comprising XGBoost, random forest, and histogram‐based gradient boosting, yielding a mean ROC‐AUC of 0.928 (Figure 4b), which is indicative of the robust discriminative power across diverse Al‐count configurations. Due to the severe class imbalance (positive class 12/210 ≈ 0.06), we adopted precision‐recall (PR [45]) analysis as a more informative alternative to ROC‐based evaluation. Unlike the ROC curve, which may overestimate performance in imbalanced scenarios, the PR curve directly evaluates the model's ability to identify the minority (stable) class. Our model achieved an average precision [46] of 0.782 (Figure 4c), which significantly exceeds the no‐skill baseline of 0.06 and demonstrates a meaningful predictive power in identifying energetically stable configurations. Given the inherent trade‐off between precision and recall [47], we selected a classification threshold that maximizes the F_1_ score [48], and obtained a precision of 82% and a recall of 75% (Figure S3) at an optimal threshold of 0.52 (Figure 4d). This F_1_‐optimal setting represents a conservative screening criterion minimizing false positives, that is, unstable structures mistakenly classified as stable, while ensuring that a representative subset of low‐energy candidates is still captured. This balance supports informed structural selection during model development and enables efficient discrimination between thermodynamically favorable and unfavorable configurations. The result further demonstrates that the model effectively learns physically meaningful features governing stability across Al‐substituted zeolite frameworks.

SHapley Additive exPlanations (SHAP) analysis was applied to the trained ensemble classifier to quantitatively elucidate the structural features governing stability in Al‐substituted Y‐zeolite frameworks (Figure 4e). Among these descriptors, the minimum Na‐Al distance (MinNaAl), angular atom‐centered symmetry function (ACSF) components characterizing O‐Na‐O angle (ACSF_Na_38 and ACSF_Na_39) and Al‐Na‐O angle (ACSF_Al_33), as well as the rank‐normalized (*_rank) average number of framework Al atoms within a six Å coordination shell of Na^+^ (NaAroundAl6_rank) emerge as the most influential predictors of stable configurations. The MinNaAl descriptor, defining the shortest distance between framework Al atoms and charge‐compensating Na^+^ ions, serves as a critical geometric indicator of structural stability, and a larger MinNaAl value is generally associated with a positive SHAP score, which reflects the increased stability of the predicated models.

**FIGURE 5:**
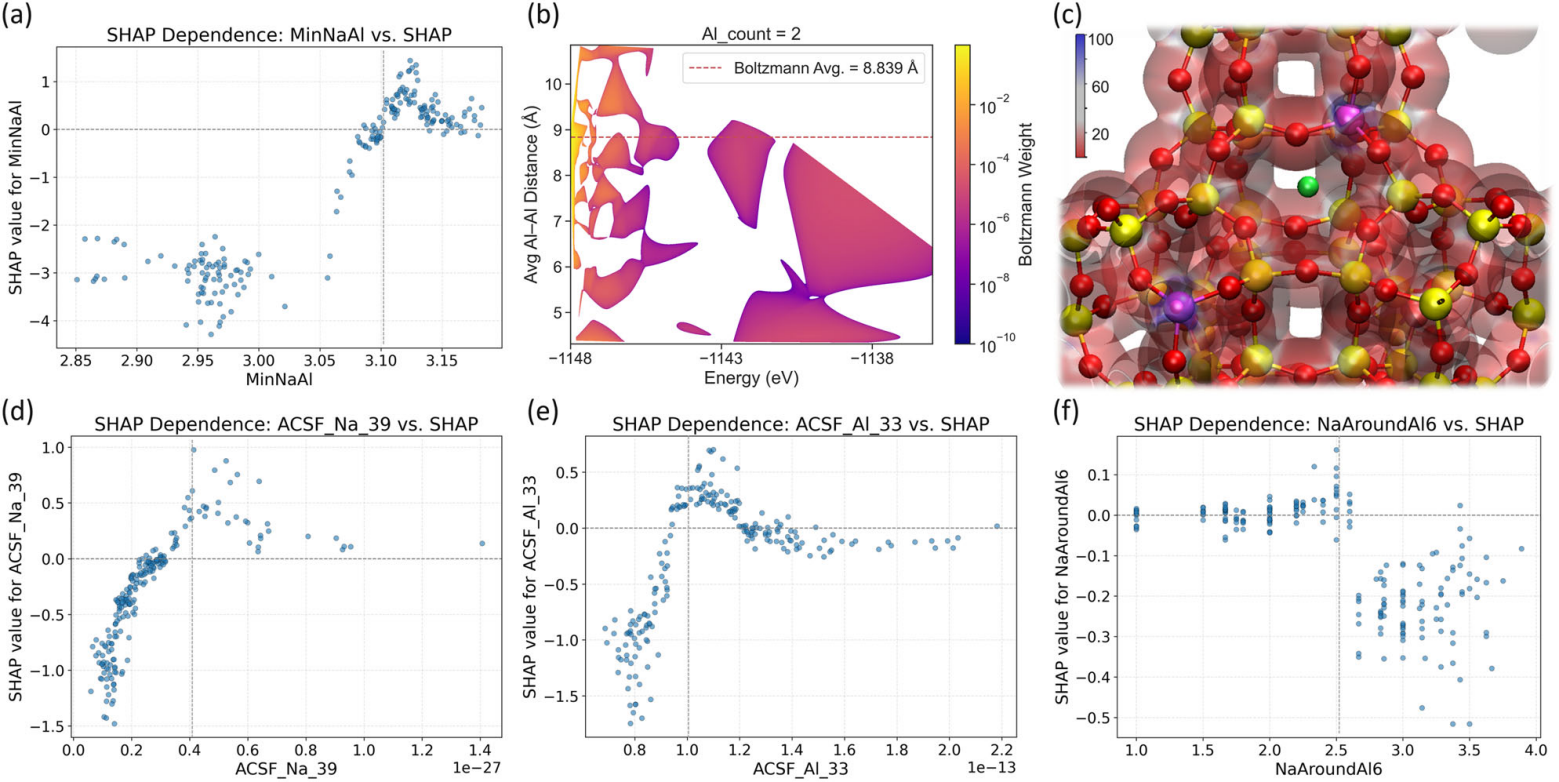
(a) SHAP dependence plot for the descriptor of MinNaAl. (b) Boltzmann‐weighted distribution of average Al‐Al distances as a function of total energy for configurations containing two Al atoms. (c) Electrostatic potential distribution of zeolite Y framework containing 2 Al atoms (gold, pink, red, and green spheres represent Si, Al, O, and Na). SHAP dependence plot for the descriptor of (d) ACSF_Na_39, (e) ACSF_Al_33, and (f) NaAroundAl6.

Detailed analysis shows that over a threshold MinNaAl of 3.1 Å, SHAP values become suddenly positive (Figure 5a), indicating that an optimal separation of Na^+^ ions and AlO_4_^−^ units increases the stability, whereas rather shorter distances decrease the stability. Structural analysis of relaxed configurations supports this trend, that is, Na^+^ ions located in 4MR typically exhibit MinNaAl values around 2.9 Å, while those in 6MR or D6R cage show values closer to 3.1 Å. These geometric and energetic trends align with the stability preferences observed across the dataset, that is, Na^+^ ion preferentially occupies 6MR, followed by D6R over a broad range of Si/Al ratios (Tables S7–S18, and S21) and 4MR as the least favorable sites. For the Si/Al ratio at 2.7 (13Al, Table S19) and 2.4 (14Al, Table S20), all eight 6MR and four D6R sites are occupied with a total of 12 Na^+^ ions; the remaining one Na^+^ ion and two Na^+^ ions can only occupy the least favorable 4MR sites. Analyzing the data along with SHAP‐derived trends in the MinNaAl descriptor reveals a consistent thermodynamic preference for Na^+^ ions to reside in 6MR. When each AlO_4_^−^ unit is locally charge‐compensated by a Na^+^ ion located within a 6MR, the resulting structure typically exhibits enhanced stability, reflecting an optimal balance between spatial proximity and favorable electrostatic interactions between Na^+^ ions and their coordinating framework.

Notably, among all configurations (Tables S7–S20), the 2Al‐2Na^+^ system (Table S8) most clearly illustrates the impact of Al and Na^+^ ions positioning on stability. In the relatively stable configurations identified (Table S8), almost all Al‐Al distances exceed the 3N‐Al separation, indicating a clear energetic penalty associated with close Al‐Al proximity. Based on these energy differences, we further analyzed the ensemble of two‐Al configurations by applying Boltzmann weighting to each structure and obtained a statistically preferred separation of over 8.8 Å (Figure 5b), which exceeds that of the 3N‐Al separation (≈4.3 Å), consistent with the expected electrostatic repulsion between adjacent AlO_4_^−^ units. These results highlight a thermodynamic bias toward spatially diluted Al configurations, even under low Al content, driven by long‐range electrostatic interactions. Electrostatic potential analysis provides further insight into the driving forces governing Al distribution during framework substitution. As Si atoms are progressively replaced by Al atoms, the introduction of negatively charged AlO_4_^−^ units generates localized regions of high electrostatic potential (Figure 5c). This inhomogeneous potential landscape gives rise to homoionic repulsion, destabilizing configurations where Al atoms are located in close proximity. To minimize this potential and stabilize the structure, each successive Al atom tends to occupy a remote position. This relationship can be found in the most stable configurations in Figure 2.

In addition to Na^+^‐Al and Al‐Al distances, ACSF_Na_38 (Figure S4) and ACSF_Na_39 (Figure 5d) are the most influential descriptors related to Gaussian‐angular G_4_ basis functions (three‐body interaction) describing the O‐Na‐O bond angle. The SHAP dependence plots reveal that ACSF_Na_38 and ACSF_Na_39 increase the predicted stability when their values exceed the characteristic thresholds (about 1×10^−14^ for ACSF_Na_38 and 3×10^−28^ for ACSF_Na_39). Notably, ACSF_Na_39 attains its maximum positive contribution at about 4×10^−28^, corresponding to an O─Na─O bond angle of approximately 180° (Figure 2a), and this explains the enhanced stability of Na^+^ ions at 6MR or D6R over those at 4MR.

Similar to ACSF_Na_38 and ACSF_Na_39, the descriptor ACSF_Al_33 (ζ = 2) encodes the three‐body Al‐Na‐O interaction between a Na^+^ ion and its adjacent AlO_4_^−^ units. As shown in the SHAP dependence plot (Figure 5e), model stability increases sharply once ACSF_Al_33 exceeds approximately 0.9×10^−13^ and reaches a maximal positive contribution at 1.0×10^−13^, indicating an essentially linear Al‐Na‐O geometry such as E8‐6MR (Figure 2). This behaviour confirms the critical structural stability enhancement of Na^+^ ion locations.

Complementary to the Na‐Al distances and the O‐Na‐Al angles, the descriptor NaAroundAl6, which quantifies the relative amount of framework Al atoms within a 6 Å radius of each Na^+^ ion, exhibits increasingly positive SHAP values at higher ranks (Figure 5f). It is also found that when the number of nearby Al atoms approaches 2.5 per Na^+^ ion, the SHAP contribution reaches a maximum, indicating that configurations with up to 2–3 surrounding Al atoms are energetically favorable, likely due to efficient electrostatic stabilization by nearby AlO_4_^−^ units with the Na^+^ ion. Beyond this threshold, SHAP values decline sharply, indicating that configurations with four AlO_4_^−^ units coordinated to the same Na^+^ within a single D6R become energetically unfavorable, where all four Al atoms are overcrowded and have the 3N‐Al separation within one D6R, resulting in a strong repulsive interaction, which outweighs the Na^+^ ions and AlO_4_^−^ units' attractive interaction. Such unfavorable configurations become only possible at very low Si/Al ratios, Si/Al = 3 to 2.4 for 12Al to 14Al substitution.

**FIGURE 6:**
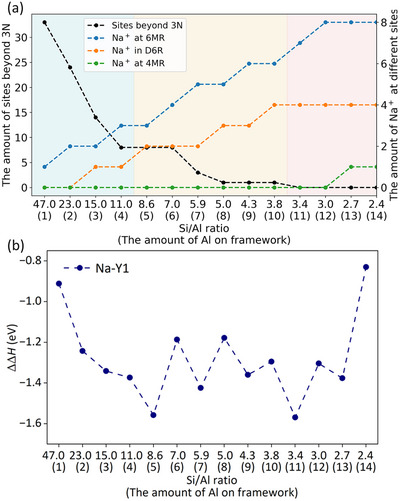
Number of available sites beyond 3N‐Al separation (black line), Na^+^ ions at the 6MR (blue line), D6R (orange line), and 4MR (green line) (a) and the computed sequential substitution enthalpy (b) at given Si/Al ratio with the number of framework Al atoms in parentheses.

All these results verify that the stability and structure of Na‐Y zeolite should be controlled by the counter‐balanced factors, that is, Na^+^ ions preferentially occupy 6MR and D6R sites, minimizing the repulsive interaction among negatively charged AlO_4_^−^ units by remote distribution, and maximizing the attractive interaction between the Na^+^ ions with the surrounding negatively charged AlO_4_^−^ units sharing 6MR and/or D6R. Having all these stable configurations in hand, we further analyzed the relationship between the location of the Na^+^ ions and the number of Al substitutions along with their seperations. As illustrated in Figure 6a, the number of 3N‐Al separations increases with the increase in Al content. From 1A to 4Al substitution, there is no 3N‐Al separation, and the repulsive electrostatic interactions among negatively charged AlO_4_^−^ units are minimized to a large extent, and at the same time, the Na^+^ ions are preferrably locacted at the 6MR and D6R. From 5Al to 10Al substitution, the number of 3N‐Al separation increases inevitably with the increase in Al content, leading to the increase of repulsive electrostatic interactions among negatively charged AlO_4_^−^ units, and this is counter‐balanced by the attractive electrostatic interactions between Na^+^ and surrounding AlO_4_^−^ units, where the corresponding Na^+^ ions are preferrably locacted at the specific D6R cages or adjacent 6MR positions. From 11A to 14Al substitution, the number of 3N‐Al separation increases drastically, and at the same time Na^+^ ions occupy not only the favorable 6MR and D6R sites but also inevitably the less favorable and only available 4MR positions. Based on these substitutions, we computed sequential substitution enthalpy for 1Al to 14 Al (Figure 6b and Table S24). All these substitutions are exothermic and favorable thermodynamically, demonstrating possible formation of Y‐zeolites with Si/Al ratio of 3, such as 2.7 and 2.4 for 13Al and 14Al substitution, respectively. This explains reasonably the preferred formation of Y‐zeolite with rather lower Si/Al ratio (≤ 3) under the synthetic condition and the difficulty to synthesize Y‐zeolite with higher Si/Al ratio (>3).

At this point, it is possible to explain the finding that the experimentally synthesized Y‐zeolites always have rather low Si/Al ratio (≤ 3) [10, 11, 12, 13, 14, 15, 16, 17, 18], although Y‐zeolites with higher Si/Al ratios are highly desired (>3), and this is due to the intrinsic nature of the well balanced attractive interaction between Na^+^ ions and AlO_4_^−^ units and the repulsive interaction among the AlO_4_^−^ units.

### 2.4 Experimental Validation

It is now interesting to compare our results with the available experimental data. At first, we compared the Si centers associated with different numbers of Al atoms, Q^4^(*x*Al)Si, which can be differentiated by the ^29^Si NMR chemical shifts, which have been widely used to infer Al distributions in zeolites (Table 1). Theoretically, there are five distinct Si centers, connected with *x* = 0, 1, 2, 3 and 4 Al centers, that is, Q^4^(0Al)Si, Q^4^(1Al)Si, Q^4^(2Al)Si, Q^4^(3Al)Si and Q^4^(4Al)Si, while the number of Q^4^(4Al)Si is always zero. For the structure with Si/Al = 3, for example, we found that there are 4 Q^4^(0Al)Si (11.1%), 18 Q^4^(1Al)Si (50.0%), 12 Q^4^(2Al)Si (33.3%), 2 Q^4^(3Al)Si (5.6%) and 0 Q^4^(4Al)Si (0.0%), and this agrees perfectly with the experimental results estimated by Oleksiak et al., [12]. For our 48T model with Si/Al = 3, all 12 Al atoms are not equally distributed, that is, two D6Rs have three Al atoms, one D6R has four Al atoms and one D6R has two Al atoms, and this structure is more stable than the one, in which each D6R has three distributed Al atoms by 0.23 eV (Table S18). This indicates the not fully balanced Na^+^/AlO_4_^−^ attractive and AlO_4_^−^/AlO_4_^−^ repulsive interaction. Excellent agreements have also been found for structures with Si/Al = 10.5 [13], 6.9 [17], and 6.2 [18]. Structures with other Si/Al ratios are collected in Table 1 and should attract potential interest for further experimental investigations.

**TABLE 1:** Compare ^29^Si NMR results between the predicted model with real samples from references.

Si/Al (n×Al)	Synthetic gel	Q^4^(0Al)Si	Q^4^(1Al)Si	Q^4^(2Al)Si	Q^4^(3Al)Si
2.5 [49]	Organic‐free & Na^+^	7.9	39.4	42.5	10.2
2.4 (14×Al)	Our model	5.9	44.1	32.4	17.7
2.7 [13]	Organic‐free & Na^+^	9.2	42.2	39.9	8.8
2.7 (13×Al)	Our model	8.6	45.7	34.3	11.4
3 [12]	Organic‐free & low Na^+^	10.0	51.0	31.0	8.0
3 (12×Al)	Our model	11.1	50.0	33.3	5.6
2.9 [11]	Organic‐free & Na^+^	14.6	40.5	36.8	8.1
3 (12×Al)	Our model	11.1	50.0	33.3	5.6
3.2 [11]	Organic‐free & Na^+^ & H_2_O_2_	19.1	42.8	31.1	7.0
3.4 (11×Al)	Our model	18.9	51.4	27.0	2.7
3.7 [18]	ChOH & Na^+^	19.1+7.1^a^	45.0	24.4	4.4
3.8 (10×Al)	Our model	26.3	47.4	23.7	2.6
4.5 [18]	CE & Na^+^	33.4+5.4^a^	40.0	15.0	6.2
4.3 (9×Al)	Our model	33.3	43.6	20.5	2.6
5 (8×Al)	Our model	40.0	42.5	15.0	2.5
6.2 [18]	ChOH & CE & Na^+^ & CBV760^b^	38.0+6.0^a^	47.1	8.9	0.0
5.9 (7×Al)	Our model	46.3	39.0	14.6	0.0
6.9 [17]	TBAOH & Na^+^ & USY_38.4_ ^b^	46.6+2.7^a^	43.3	7.4	0.0
7 (6×Al)	Our model	52.4	33.3	14.3	0.0
9.0 [17]	TBAOH & Na^+^ & USY_38.4_ ^b^	55.2+3.74^a^	37.8	3.2	0.0
8.6 (5×Al)	Our model	62.8	30.2	7.0	0.0
10.5 [13]	TBAOH & TMAOH& low Na^+^	59.86+4.91^a^	32.9	2.4	0.0
11 (4×Al)	Our model	68.2	31.8	0.0	0.0
15 (3×Al)	Our model	73.3	26.7	0.0	0.0
23 (2×Al)	Our model	82.6	17.4	0.0	0.0

Abbreviations: CE, 15‐crown‐5; ChOH, choline hydroxide; TMAOH, tetramethylammonium hydroxide; TBAOH, tetrabutylammonium hydroxide.

^a^ Si(3Si,1OH): −102 ppm.

^b^ Zeolite seeds.

**FIGURE 7:**
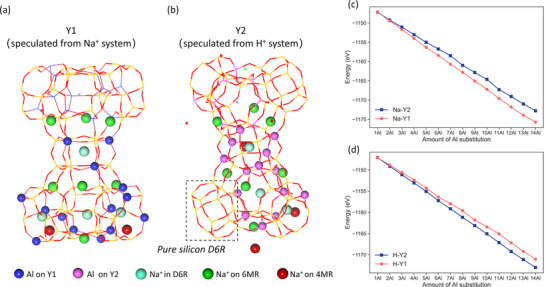
(a) Al distribution based on Na^+^‐balanced model (Y1). (b) Al distribution based on H^+^‐balanced model (Y2). Stability of (c) Na‐Y and (d) H‐Y with different Al distribution.

In addition, the inferred Al distribution was also compared with the previous computational result (Figure 7). Our current Na‐Y1 model, deduced based on Na^+^ ions resulting in 4N‐Al and 5N‐Al separations at low Al content and then 3N‐Al separation at high Al content, is different from the previously reported H‐Y2 model deduced based on H^+^ ions resulting only in 3N‐Al separation, which is thermodynamically more stable than 4N‐Al separation [38]. To explain this energetic preference, we analyzed the corresponding Al‐O/OH and Si‐O/OH distances in H‐Y2 and found that the Al‐OH distances in 3N‐Al is shorter than that of 4N‐Al (1.886/1.878 vs. 1.903/1.909 Å), and they are longer than the corresponding Si‐O distance (1.624–1.633 Å), indicating a stronger structural strain in 4N‐Al than in 3N‐Al, explaining the higher thermodynamic stability of 3N‐Al than 4N‐Al.

We compared these two models in two ways. At first, we converted the H‐Y2 model to the corresponding Na‐Y2 model by replacing H^+^ ions with Na^+^ ions, preferably located at 6MR, D6R, and 4MR. Structurally, Na‐Y1 is different from Na‐Y2; that is, the distribution of Al atoms is more separated in Na‐Y1, while more aggregated in Na‐Y2 (Figure S5). For 14Al substitution of the 48T model, for example, 14Na‐Y1 has four D6R (Figure 7a), and there are two D6R, each with four Al atoms, and there are also two D6R, each with three Al atoms (4+4+3+3). Although 14Na‐Y2 also has four D6R, the Al atoms are differently distributed (Figure 7b), that is, there are two D6R, and each has six Al atoms, and one D6R has two Al atoms, and one pure silicon D6R does not have an Al atom (6+6+2+0). Based on the fully optimized structures, Na‐Y1 is more stable than Na‐Y2, and the energy difference increases with the increase of the number of Al substitutions (Figure 7c), for example, 14Na‐Y1 is more stable than 14N‐Y2 by 2.94 eV.

Next, we converted our Na‐Y1 model to the corresponding H‐Y1 model by replacing the Na^+^ ions by H^+^ ions with the bridged OH groups towards the super cage. Full structure optimization shows that H‐Y1 is less stable than H‐Y2, and the energy difference increases with the increase of the number of Al substitutions (Figure 7d), and 14H‐Y1 is less stable than 14H‐Y2 by 1.89 eV.

This reverse stability order between Na‐Y1 and Na‐Y2, as well as between H‐Y1 and H‐Y2, highlights the different roles of Na^+^ ions and H^+^ ions in determining the framework Al distribution and stability. This will affect the acid strength and catalytic activity.

### 2.5 Y‐zeolite With Non‐exchangeable Na^+^ Ions

Experimental isotherm ion‐exchange showed that for the Na‐Y zeolite with a Si/Al ratio of about 2.4, about 32% Na^+^ ions in sodalite cage and D6R are non‐exchangeable, that is, 16 non‐exchangeable Na^+^ ions per unit cell (192 T) [44]. In our primitive 48T model with 14Al substitution (14Na‐Y1), there are 14 Na^+^ ions, eight at eight 6MR, four encapsulated in four D6R, and two on two 4MR. The four Na^+^ ions encapsulated in D6R units, about 28% (4/14) of the total Na^+^ ions, allows rough comparison with the experimentally measured value of approximately 32% from the result of isotherm ion‐exchange.

**FIGURE 8:**
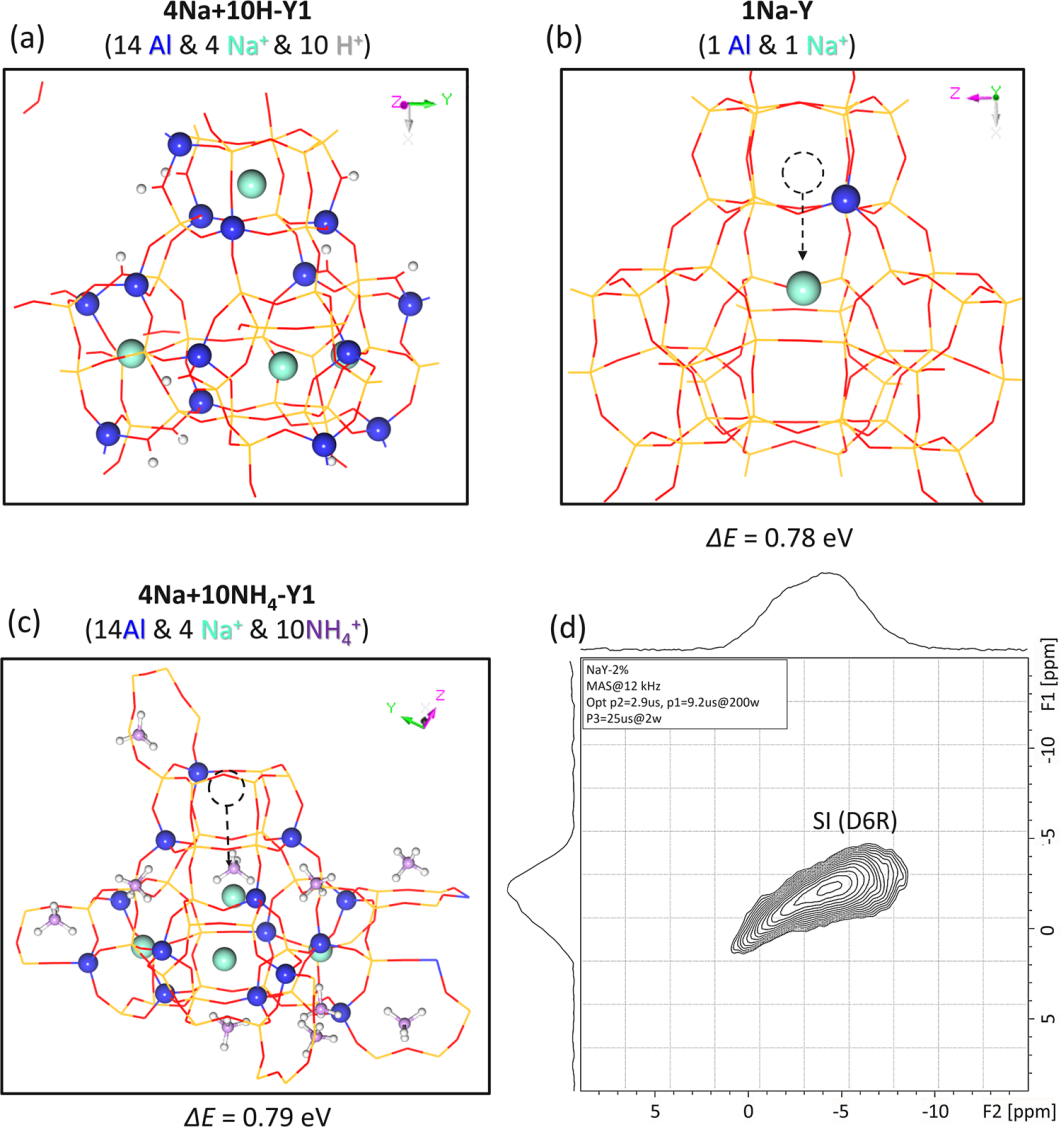
Model of (a) 14Al and 4Na^+^ ions in D6R and 10H^+^ (4Na+10H‐Y1). Energy difference between Na^+^ ion in D6R and sodalite cage: (b) with 1Al and 1Na^+^ ion (1Na‐Y), (c) with 14Al and 4Na^+^ ions in D6R and 10 NH_4_^+^ ions (4Na+10NH_4_‐Y1). (d) ^23^Na MQMAS NMR of Na‐H‐Y (2 wt.% Na^+^ ions).

To check this non‐exchangeable behavior, we constructed several models with one Na^+^ ion initially put in the sodalite cage. For the 14Na‐Y1, one Na^+^ ion put in the sodalite cage was optimized back to the D6R. The same result is also found for the mixed Na‐H‐Y1 model with four Na^+^ ions and 10 H^+^ ions (4Na+10H‐Y1, Figure 8a), and one Na^+^ ion put in the sodalite cage was optimized back to the D6R. Finally, we used the 1Al model with 1Na^+^ ion (1Na‐Y, Figure 8b) and 14Al model with four Na^+^ ions in D6R and 10 NH_4_^+^ ions at 6MR and 4MR (4Na+10NH_4_‐Y1, Figure 8c) and found that the Na^+^ ion at the D6R is more stable than that at the sodalite cage by about 0.78 and 0.79 eV, respectively. All these indicate the enhanced stability of Na^+^ ions in the D6R.

Furthermore, we validated the unexchangeability of Na^+^ ions in D6Rs in our model (Figure S7a) by comparing the calculated isotropic ^23^Na NMR chemical shifts (Figure S7b) with the available experimental data of Na‐Y samples. The calculated ^23^Na chemical shifts in 6MR are −0.68, −10.14, −10.32, and −13.12 ppm, which closely match the experimental values at −2 and −14 ppm [50]. For Na^+^ ions located in D6Rs, the computed ^23^Na chemical shift is approximately −9.6 ppm, consistent with the experimental value at near −7.3 ppm [51]. Our experimental results of ^23^Na solid‐state NMR Spectroscopy (Figure 8d) of Y‐zeolites with about 2 wt.% Na^+^ ions content shows that the signal around −6 ppm is retained, confirming the non‐exchangeable Na^+^ ions are located in D6R. These results collectively support the structural validity of the Na^+^ ions' positions proposed in our model.

### 2.6 Acid Strength on Y‐Zeolite With Non‐Exchangeable Na^+^ Ions

**FIGURE 9:**
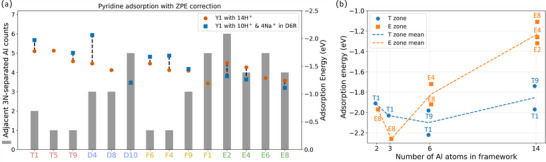
Pyridine adsorption energy and corresponding adjacent 3N‐separated Al counts of acid sites on Y1 with and without Na^+^ ions in D6R (a). Pyridine adsorption energy changes in the T zone and E zone with increasing number of Al atoms (b).

Having these results in hand, we estimated the acid strength of 14H‐Y1 and 4Na‐10H‐Y1 based on the computed adsorption structures and energy of pyridine (Figure 9a; Tables S4 and S5). It is noted that the computed adsorption energy as a measure for acid strength includes not only the protonation energy but also the interaction between the protonated probe molecule and the local environment, as discussed in previous studies [38, 52, 53, 54, 55, 56, 57, 58]. For our 14H‐Y1 model, pyridine adsorption energy decreases with the increase in the number of adjacent 3N‐Al separations. At T zone (Figures 1 and 7a) having only three Al atoms situated in D6R (T1, T5, and T9) with one or two adjacent 3N‐Al separation, pyridine has stronger adsorption energies, while at D zone also having three Al atoms situated in D6R (D4, D8 and D10) with three or five adjacent 3N‐Al separation, pyridine has lower adsorption energies, and the largest difference between T1 and D10 is 0.54 eV (Table S5). At the F zone, having four Al atoms situated in D6R, the adsorption energy of pyridine also decreases with the number of adjacent 3N‐Al separations, but in rather small extent. At the E zone, having four Al atoms situated in D6R, no such relationship can be clearly found. Nevertheless, the largest pyridine adsorption energy has been found at the T zone with three Al atoms in D6R. It can be roughly concluded that aggregated Al distribution with 3N‐Al separations can weaken Brønsted acid strength, and this agrees with the previous reports [59].

Figure 9b presents the acid strength changes in the T and E zones with the increase of Al atoms. Notably, the acid strength of the T1 site increases when the third Al atom at the T5 site, located at a 4N‐Al distance from T1, and the corresponding Na^+^ ion is located within the D6R of the T region. Moreover, even as the number of Al atoms increased up to 14, the T zone acid sites are surrounded by only one or two Al atoms distributed at 3N‐Al separation (Figure 9a). As a result, the acid strength of the T zone exhibits only modest variations across this range. Conversely, the E region experiences a substantial reduction in acid strength with more closely spaced Al atoms. This denser local distribution significantly weakens the acid strength of the E zone.

**FIGURE 10:**
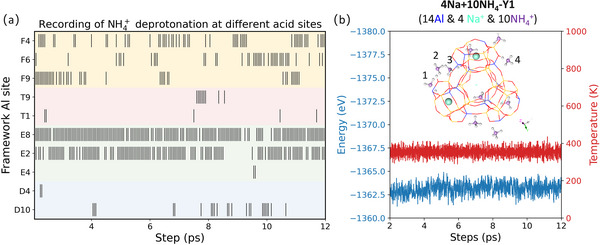
NH_3_ population in different zones based on coordination number including all N‐H distances shorter than 1.3 Å (a), variation of temperature and energy (b) as a function of simulation time (the inserted structure in (b) is the snapshot at 12 ps).

With four non‐exchangeable Na^+^ ions located in D6R units, 4Na+10H‐Y1 exhibits stronger pyridine adsorption than 14H‐Y1, originating from the electrostatic stabilization of the conjugated AlO_4_^−^ sites by the encapsulated Na^+^, and such interaction results in longer O^…^H (1.647 vs. 1.532 Å) and shorter N‐H (1.061 vs. 1.094 Å) distances compared to those of 14H‐Y1, which does not have such an interaction. Such an electrostatic interaction can be seen from the charge‐density‐difference analysis (Figure S8). The rather larger enhancement can be found in T and D zones, that is, 0.20 and 0.51 eV at T1 and D4, respectively, and the changes in F and E zones are rather small. To confirm this positional difference in acid strength, we used the 4Na+10NH_4_‐Y1 for AIMD simulations under ion‐exchange conditions (350 K). To exclude the influence of the surrounding AlO_4_^−^ on NH_4_^+^ ions deprotonation, all NH_4_^+^ ions in 4Na+10NH_4_‐Y1 were uniformly placed on the 4MR at the corresponding acid sites, despite the fact that the location of NH_4_^+^ ion depends on the surrounding environment, for example, in 13Na+1NH_4_‐Y1, the NH_4_^+^ ion preferably located on 6MR (Table S22), and in 4Na+9H+1NH_4_‐Y1 (Table S23), the NH_4_^+^ ion tends to locate on the 6MR with two or three Al atoms (Figure S9a,b), while for the 6MR containing only one Al atom, the NH_4_^+^ ion tends to locate on the corresponding 4MR (Figure S9c,d). Within 2 and 12 picoseconds (ps) of AIMD simulations, we analyzed the NH_3_ population from NH_4_^+^ deprotonation based on the coordination numbers (Figure 10a) and found that NH_3_ appears predominantly at E and F zones (F4, F6, F9, E8, and E2 sites). At 12 ps, NH_4_^+^ deprotonation takes place and releases NH_3_ at the F4, F6, E8, and E2 sites (Figure 10b). This site‐dependent behavior can also explain the differences in pyridine adsorption arising from variations in the local reaction environment.

**FIGURE 11:**
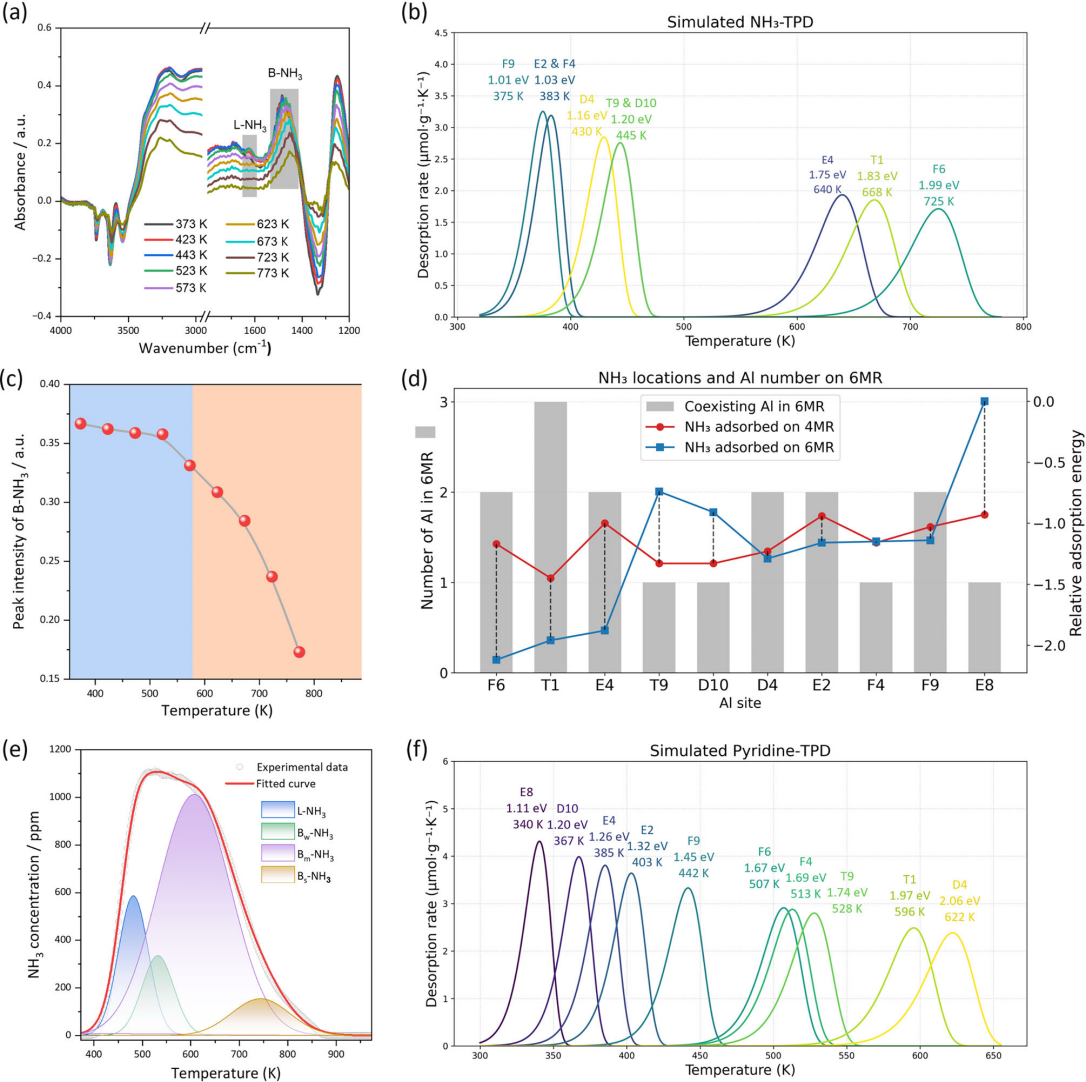
(a) NH_3_‐DRIFTS of Na‐H‐Y. (b) Simulated NH_3_ (pre‐exponential factor = 10^11^ s^−1^) desorption temperatures. (c) Changes in the intensity of NH_3_ adsorbed on Brønsted acid sites. (d) Relationship of NH_3_ adsorption energy on different acid sites with the number of coexisting Al in 6MR. (e) NH_3_‐TPD of Na‐H‐Y. (f) Simulated pyridine (pre‐exponential factor = 10^14^ s^−1^) desorption temperatures.

Next, we compared the simulated desorption temperature with the temperature‐programmed desorption of ammonia (NH_3_‐TPD), which has been widely used as a probing molecule to determine the number and strength of acid sites in zeolites (Figure 11). At first, we recorded IR spectra of NH_3_ and found both Lewis and Brønsted acid sites at low temperature (Figure 11a) and with the increase of temperature the intensity of the Lewis acid sites decreases rapidly and disappears at around 573 K, whereas the Brønsted acid bands remained nearly unchanged (Figure 11c). The same trend is also found for the IR spectra of pyridine (Figure S10). With the further increase of temperature, the intensity of the Brønsted acid sites also decreases and disappears at around 600 K. For NH_3_ adsorption on the Brønsted acid sites, our simulated NH_3_‐TPD using the Redhead equation in the range from 375 to 725 K (Figure 11b) agrees with the reported experimental range (380–700 K [60]) and our new measurement (Figure 11e). Based on the experimental classification of the NH_3_‐TPD [61, 62, 63], we assigned our computed desorption temperature at 725, 640–670, and 380–440 K roughly to strong, medium, and weak acid sites, respectively.

**FIGURE 12:**
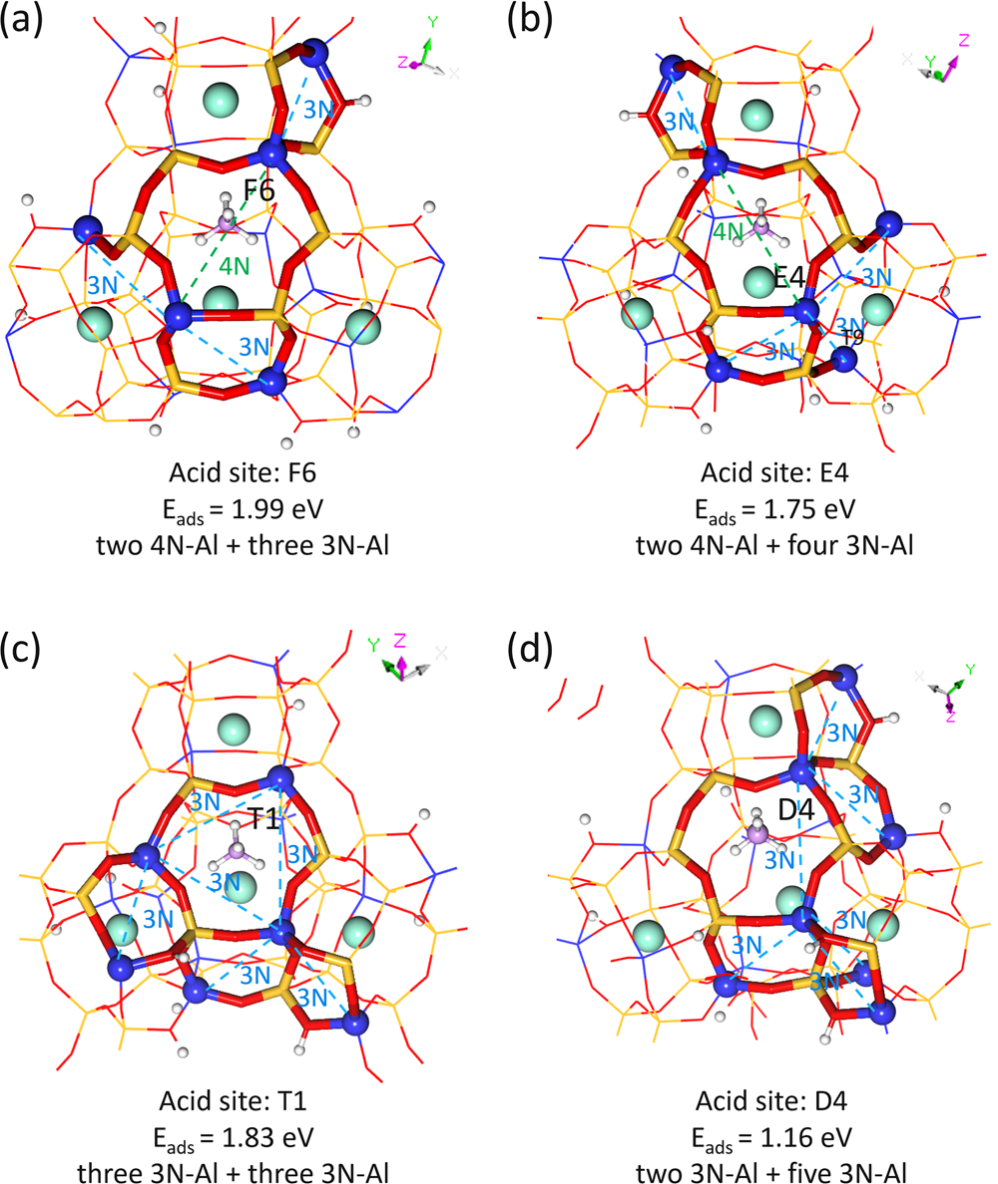
Local environment of adsorbed NH_4_^+^ on 6MR with different numbers of 4N‐Al and 3N‐Al separations based on the 4Na+10H‐Y1 model.

To understand the effect in determining the desorption temperature of NH_3_, we further analyzed the local environments for the adsorbed NH_4_^+^ based on the 4Na+10H‐Y1 model (Figures 11d and 12). For strong adsorption at F6 (Figure 12a), the adsorption energy of NH_3_ is −1.99 eV, and the adsorbed NH_4_^+^ is located on a 6MR containing two diagonally located Al atoms at 4N‐Al separation. As 4N‐Al represents the maximum Al‐Al separation within a 6MR, the well‐separated AlO_4_^−^ units stabilize a single NH_4_^+^ and minimize the destabilizing influence of 3N‐Al sites. For medium adsorption at E4 (Figure 12b), the adsorption energy of NH_3_ is −1.75 eV, and the adsorbed NH_4_^+^ is located on a 6MR containing one 4N‐Al separation and four 3N‐Al separations. The denser 3N‐Al environment reduces the stabilization by the 4N‐Al separation. For medium adsorption at T1 (Figure 12c), NH_3_ has an adsorption energy of −1.83 eV, and the adsorbed NH_4_^+^ is located on a 6MR containing three Al atoms at 3N‐Al separation. Although the AlO_4_^−^ units contribute to stabilization, the predominant 3N‐Al interaction leads to weaker adsorption compared to the configuration at F6. For weak adsorption at D4 (Figure 12d), the adsorption energy of NH_3_ is −1.16 eV, and the adsorbed NH_4_^+^ is located on a 6MR containing two Al atoms in 3N‐Al separation, along with five additional 3N‐Al separations around. Based on these analyses, we might propose the dependence of NH_3_ adsorption energy on the local environment of containing different numbers of 4N‐Al and 3N‐Al separations to differentiate the strong, medium, and weak acid sites observed in the experiment of NH_3_‐TPD.

The same results have also been found for the desorption of pyridine. For example, the simulated pyridine‐TPD from 340 to 622 K (Figure 11f) closely matches the experimentally determined range from 364 to 623 K [64], and the desorption temperatures at 596–622, 507–528 and 340–442 K are also close to the experimentally estimated values at 623, 523, and 423 K from temperature‐dependent pyridine‐IR spectroscopy, classified as strong, medium, and weak Brønsted acid sites [65, 66].

However, detailed analysis shows that the adsorbed pyridinium ions and ammonium ions are located at different sites due to not only their different electronic property (*sp* ^2^ vs. *sp* ^3^ N) but also their geometric differences, such as NH_4_^+^ ions can have up to tridentate hydrogen bonding with the surrounding framework oxygen atoms (Tables S22 and S23; Figure 12), while pyridine can have only monodentate hydrogen bonding predominantly residing within the super cage of Y‐zeolite. This shows the confinement effect of different sites, and therefore, the sequence of the acid strength based on pyridine adsorption is different from that based on ammonia adsorption. Despite the interesting results for pyridine adsorption, especially when compared to experimental results, it is not readily justified to claim that pyridine adsorption energy is a measure of acid strength.

### 2.7 Catalytic Activity

Finally, we tested the catalytic activity of propane cracking by using two different acid models, that is, 14H‐Y1 and 4Na+10H‐Y1 (Figure S11). Since the T1 site in the T zone has the strongest pyridine adsorption energy, we used the T1 site for our comparison. All structural details of the initial states, transition states, and final states are given in the Supporting Information. Compared 14H‐Y1 with 4Na+10H‐Y1 with 14Al substitution, it is found that 4Na+10H‐Y1 has not only stronger pyridine adsorption energy (−1.97 vs. −1.77 eV) but also lower activation barrier (2.00 vs. 2.27 eV) and less endothermic reaction energy (1.95 vs. 2.25 eV). This energetic effect of 4Na+10H‐Y1 comes from the electrostatic stabilization of the conjugated AlO_4_^−^ sites by the encapsulated Na^+^, and such interaction results in longer C‐H (1.173 vs. 1.184 Å) and shorter O^…^H (2.072 vs. 2.068 Å) distances compared to those of 14H‐Y1, which does not have such interaction. Such an electrostatic interaction can be seen from the charge‐density‐difference analysis (Figure S12). Therefore, not only the acid strength but also the local electrostatic environment is responsible for the enhanced catalytic activity, as proposed by Iglesia and co‐workers [53, 54, 55, 56, 57]. It is noted that a similar synergistic effect has been reported for propane cracking on multicharged extra‐framework aluminum species and FAU zeolite framework [67].

## 3 Conclusion

For aluminum‐substituted silica zeolites, the most important features are the stability related to the Si/Al ratio as well as the strength and number of the Brønsted acid sites, which play a central role in catalysis, such as catalytic cracking, hydrocracking, adsorption, and separation in petroleum chemistry and engineering. Since the acid sites originate from the charge‐balanced Al substitution, determining the distribution of framework Al atoms and characterizing their respective acid properties has long been a complex challenge in scientific research and industrial applications.

In addition to ongoing experimental studies using modern techniques, theoretical studies have been conducted to investigate the distribution of Al atoms in the framework, the resulting structures and stability, the number and strength of the Brønsted acid sites, and the respective catalytic activity in many reactions. However, previous theoretical studies have simply used proton (H^+^) as the charge‐balance agent and ignored the role of alkali ions (such as Na^+^) in the initial stage of the synthesis under basic conditions. Based on the difference between H^+^ and Na^+^, this simplification using H^+^ as a charge‐balance agent might lead to a different distribution of framework Al atoms, resulting in different acid properties and catalytic activity from that using Na^+^ ions as a charge‐balance agent.

Based on the initial stage of the synthesis under alkali conditions using NaOH, we carried out systematic investigations into the topological structures of Al‐substituted Y‐zeolite based on density functional theory computations, descriptors deduced from machine learning, and AIMD simulations accompanied by an experimental analysis. Different results from previous studies with H^+^ as a charge‐balancing agent have been obtained. The first difference is the distribution of framework Al atoms. Using H^+^ as a charge‐balancing agent, the distribution of framework Al atoms prefers the next‐nearest‐neighbor separation (3N‐Al); while using Na^+^ ions as a charge‐balancing agent, the distribution of framework Al atoms prefers the next‐next‐nearest‐neighbor separation (4N‐Al) and the next‐next‐next‐nearest‐neighbor Al separation (5N‐Al). This preference is due to the cooperative balance between the Na^+^/AlO_4_^−^ attractive and the AlO_4_^−^/AlO_4_^−^ repulsive interaction, and the preferential occupancy of Na^+^ ions following the order six‐membered rings (6MR) > double six‐membered rings (D6R) > four‐membered rings (4MR). Based on these substitutions, the computed sequential substitution enthalpy shows the possible and thermodynamically favorable substitution of up to 14 Al, which has a Si/Al ratio of 2.4. This explains reasonably the preferred formation of Y‐zeolite with rather lower Si/Al ratio (≤ 3) under the synthetic condition and the difficulty to synthesize Y‐zeolite with higher Si/Al ratio (>3).

Our structural topology agrees with the results deduced from the ^29^Si NMR measurement in differentiating the environment of the Si center in association with the next Al centers and the best agreement is found for synthesized Y‐zeolite under only basic condition without any organic directing agents for Si/Al = 3, where the Al atoms are not equally distributed among these D6Rs, and this structure is more stable than the one with equally distributed Al atoms by 0.23 eV. This demonstrates once again the different contributions of Na^+^/AlO_4_^−^ attractive and AlO_4_^−^/AlO_4_^−^ repulsive interaction and the different location of Na^+^ ions.

It is also confirmed that there are 16 non‐exchangeable Na^+^ ions located within the D6Rs, in agreement with the experimental findings, and these non‐exchangeable Na^+^ ions have been overlooked in the previous theoretical studies in Y‐zeolites. The acid strength of Y‐zeolite with non‐exchangeable Na^+^ ions can be classified as strong, medium, and weak, as confirmed by the experimentally determined temperature‐programmed desorption of ammonia and pyridine, supported by DFT computations. The experimentally assigned acid strengths can be roughly explained by local environment‐dependent adsorption energy; that is, the more dispersed the distribution of Al atoms in the framework, the higher the adsorption energy of pyridine and ammonia, and the stronger the acid strength. However, detailed analysis shows that ammonia and pyridine have different adsorption configurations in describing different acid strengths based on the nature of ammonia and pyridine, as well as the environment. Finally, Y‐zeolite with non‐exchangeable Na^+^ ions exhibit higher catalytic activity in propane cracking as compared to pure H‐Y zeolite.

This work provides new insights into the effect of Na^+^ ions as a charge‐balancing agent in determining the distribution of framework Al atoms of Y‐zeolite, based on DFT computation and machine learning methods, and the resulting acid strength and catalytic activity. More importantly, the well‐defined Na‐Y structure provides a solid foundation for the study of Y‐type zeolite modification guidelines for industrial applications. This funding is not only interesting for Y‐zeolites but also for other zeolites in industrial applications.

## Conflicts of Interest

The authors declare no conflicts of interest.

## Supporting information




**Supporting File 1**: anie71359‐sup‐0001‐SuppMat.docx.

**Supporting File 2**: anie71359‐sup‐0002‐SI‐Structure‐Coordinates.7z.

## Data Availability

The data that support the findings of this study are available in the supplementary material of this article.
