# Supplementary material for: Encapsulated Non‐Exchangeable Na+ Ions Determining the Upper Limit of Al Inclusion in FAU—A Multiscale Simulation
Source: Angew Chem Int Ed Engl. 2026 Feb 3;65(11):e24044. doi: 10.1002/anie.202524044 (PMC12970491; doi:10.1002/anie.202524044)
Supplement: Supplementary file 1 — Supporting File 1: anie71359‐sup‐0001‐SuppMat.docx. [file ANIE-65-e24044-s002.docx]

Encapsulated Non-Exchangeable Na^+^ Ions Determining the Upper Limit of Al Inclusion in FAU‒A Multiscale Simulation

Qi Dong^[a, b]^, Tao Zhang^[c]^, Chuanhao Zhang^[a]^, Tong Zhang^[d]^, Yanze Du^[d]^, Jinghong Ma^[a]^, Ruifeng Li*^[a]^, Bo Qin*^[d]^, Haijun Jiao*^[b]^

[a] Qi Dong, Chuanhao Zhang, Prof. Dr. Jinghong Ma and Prof. Dr. Ruifeng Li
State Key Laboratory of Clean and Efficient Coal Utilization, College of Chemistry and Chemical Engineering
Taiyuan University of Technology
79 West Yingze Street, 030024 Taiyuan, China
E-mail: [rfli@tyut.edu.cn](mailto:rfli@tyut.edu.cn)

[b] Qi Dong and Prof. Dr. Haijun Jiao
Leibniz-Institut für Katalyse e.V.
Albert-Einstein-Straße 29a,18059 Rostock, Germany
E-mail: [haijun.jiao@catalysis.de](mailto:haijun.jiao@catalysis.de)

[c] Tao Zhang
School of Chemistry and Life Resources
Renmin University of China, 100872 Beijing, China

[d] Bo Qin, Tong Zhang, Yanze Du
SINOPEC Dalian Research Institute of Petroleum & Petrochemicals Co., Ltd, 116045 Dalian, China
E-mail: [qinbo.fshy@sinopec.com](mailto:qinbo.fshy@sinopec.com)

**Table of Contents**

1. **Experiment Details**

1.1 Materials and Ion-exchange Process3

1.2 Characterizations3

**2. Computational Details**

2.1 DFT Calculation4

**Table S1.** ENMAX and ENMIN values of the PAW potentials 5

**Table S2.** Total energies (*E*_tot-cutoff energy_) and energy differences ∆*E_cutoff energy_* (eV) of configurations in Figures 1-3 (cutoff energy = 500 or 550 eV­­)­5

**Figure S1.** Structural comparation of configurations in Figure 1 optimized at different cutoff energies6

**Table S3.** Specific data configurations in Figure 1-3 6

**Table S4.** Specific data of ZPE-corrected pyridine adsorption energy (E_ads_) on 4Na-10H-Y1 7

**Table S5.** Specific data of ZPE-corrected pyridine adsorption energy (E_ads_) on 14H-Y17

2.2 ^23^Na NMR Calculation8

2.3 Ab Initio Molecular Dynamics (AIMD) Simulations 9

2.4 Thermal Desorption 10

**3. Machine Learning (ML) Details** 11-13

**4. Supplementary Figure**

**Figure S2.** Specific framework T-sites labeled at E, F, D, and T zones.14

**Figure S3.** Model precision and recall.15

**Figure S4.** SHAP dependence plot for the descriptor of ACSF_Na_38.16

**Figure S5.** Al distribution and the corresponding Na^+^ positions on Y-1 and Y-2 (Si/Al = 2.4-47).17

**Figure S6.** SHAP dependence plot for (a) SOAP_Na_PC1. (b) SOAP_Na_-PC1. (c) SOAP_Na_PC2. (d) SOAP_Na_-PC218-19

**Figure S7.** (a) Structural model of Na-Y1-cluster. (b) Calculated isotropic chemical shifts for the eight ‒ions in Na-Y1-cluster. 20

**Figure S8.** Charge-density-difference analysis of pyridine adsorbed on (a) 4Na+10H-Y1, (b) 14H-Y1 21

**Figure S9.** Configurations of F6-6MR (a), F6-4MR (b), T9-6MR (c) and T9-4MR (d) in Table S23. 22

**Figure S10.** Py-IR of Na-H-Y. 23

**Figure S11.** (a) Energy profiles of propane cracking. (b) Local structures of initial states (IS), transition states (TS), and reaction intermediates (Int) for propane cracking on 14H-Y1 and 4Na-10H-Y124

**Figure S12.** Charge-density-difference analysis of propane cracking for the initial state (IS), transition state (TS) and intermediate (Int) on (a) 14H-Y1 and (b) 4Na+10H-Y1.25

**5. Other Supplementary Tables**

**Table S7 to Table S20.** Structural energies of Al distribution and corresponding Na^+^ positions on Y-zeolites with 1 to 14 Al atoms on framework (Si/Al=47-2.4).26-40

**Table Sn-1.** Additions to Table Sn 27-40

**Table S21.** Energy difference of Na^+^ positions with increasing Al atoms.41

**Table S22.** Energy difference of one NH_4_^+^ positions on Na-Y1 with different Si/Al42

**Table S23.** Energy difference of one NH_4_^+^ positions on Y1 (4Na+1NH_4_+9H) with different Si/Al43

**Table S24.** Sequential substitution enthalpy44

**6. References** 45-46

**Experimental Details**

**1.1** **Materials and Ion-exchange Process**

Y-zeolite samples were supplied by Sinopec Dalian (Fushun) Research Institute of Petroleum and Petrochemicals. For the procedure of ammonium ion-exchange, Na-Y zeolite (about 12 wt.% Na_2_O) was mixed with a 1 M aqueous solution of ammonium nitrate (NH_4_∙NO_3_) at a solid-to-liquid ratio of 1:10 (g·mL^-1^). The mixture was stirred at 500 rpm for 1 h at 80 °C, followed by filtration and triple washing with deionized water. The resulting ion-exchanged zeolite was dried at 100 °C for 24 h to afford a product with a reduced Na_2_O content of 4 wt.%. This procedure was repeated twice, resulting in a final Na_2_O content stabilized at approximately 2 wt.%, named Na-H-Y.

**1.2 Characterizations**

Elemental compositions of the zeolite samples were determined by X-ray fluorescence (XRF) spectroscopy using a ZSX Primus IV spectrometer (Rigaku, Japan). The samples were finely ground, pressed into pellets and analyzed after instrument calibration. The X-ray tube was operated at a maximum power of 4 kW, equipped with a front-window Rh anode, with an operating voltage up to 60 kV and a maximum current of 150 mA. The Be window had a thickness of 30 μm. The system stability was maintained within ±0.0002% under a 1% fluctuation in the external circuit. Data acquisition was performed following standard operating protocols.

Temperature-programmed desorption of NH_3_ (NH_3_-TPD) was performed using an online FTIR spectrometer (Nicolet Antaris IGS Gas Analyzer) to monitor desorbed NH_3_. 0.10 g of catalyst was pretreated in flowing air (100 mL min^-1^) at 500 °C for 1 h, then cooled to 100 °C. NH_3_ adsorption was carried out at 100 °C until saturation, followed by N_2_ purging until the NH_3_ concentration decreased below 10 ppm. Desorption was recorded during heating from 100 to 700 °C in N_2_ (100 mL min^-1^) at a ramp rate of 10 °C min^-1^.

The measurements of in situ diffuse reflectance infrared Fourier transform spectroscopy (in situ DRIFTS) and pyridine infrared spectroscopy (Py-IR) were carried out on a Bruker INVENIO S spectrometer equipped with a liquid-nitrogen-cooled mercury cadmium telluride (MCT) detector using different in-house-built reaction cells. For in situ DRIFTS, about 0.06 g of catalyst was packed into a custom-designed high-temperature DRIFTS cell. The catalyst was calcined in situ at 500 °C for 1 h in flowing air, cooled to the target temperature, and a background spectrum was recorded. A flow of 500 ppm NH_3_ in Ar (50 mL min^-1^) was then introduced, and spectra were collected at a resolution of 4 cm^-1^ with 100 scans per spectrum. For Py-IR, self-supporting wafers were mounted in a custom-built transmission cell. The wafers were pretreated under vacuum at 100, 200, and 300 °C for 1 h. Pyridine vapor was then introduced for 30 min, followed by evacuation at the corresponding temperature for 15 min prior to recording the spectra.

^23^Na MQMAS NMR experiments were performed on a Bruker Avance III 500 spectrometer (11.7T) with resonance frequencies of 132.29 MHz for ^23^Na. 2D ^23^Na MQMAS NMR spectra were recorded using a 4 mm MAS NMR probe with a spinning rate of 12 kHz, accumulating 720 transients for each t1 increment at a recycle delay of 0.1 s.

**Computational Details**

**DFT calculation**: Plane wave-based pseudo-potential code implemented in the Vienna ab initio simulation package (VASP, 6.5.1) was used. The electron-ion interaction was described by the projector augmented wave method (PAW). The exchange and correlation energies were described with the Perdew-Burke-Ernzerhof scheme in the generalized gradient approximation (GGA-PBE).^[1,2]^ Structures and lattice parameters were fully relaxed and optimized with D3 parameter of Grimme to include dispersion correction.^[3]^ Cutoff energy of the plane wave is 500 eV, and the reasonability was checked in Table S1, S2 and Figure S1.

To deal with the orbitals occupied by the fractions near the Fermi level and the energy level broadening, the Gaussian smearing method with σ = 0.05 eV was used. The convergence criterion for elec­tron and ion steps is 10^−6^ eV in total energy and 0.02 eV/Å in force, respectively. A 1 × 1 × 1 Gamma centered Monkhorst-Pack k-point grids was used for sampling the Brillouin zone.^[4]^ Frequency analysis and zero-point energy (ZPE) of the adsorbed probe molecule were calculated using relaxed structure. Adsorption energy (∆*E*_ads_) of species X is defined as $\Delta E_{ads} = E(X/Y) - E(Y) - E(X)$, where $E(X/Y)$is the total energy of 4Na-10H-Y1 or 14H-Y1 zeolite and adsorbent $(X)$ in equilibrium; $E(Y)$is the total energy of bare 4Na-10H-Y1 or 14H-Y1 zeolite; $E(X)$is the total energy of free species $X$ in gas phase. To estimate the thermodynamic probability of Al substitution, we calculated sequential Al substitution enthalpy (∆∆*H*), which is defined as $\Delta\Delta H=\left[ E(^{48-(n+1)}{Si}_{(n+1)H}^{n+1}{Al}_{zeo})+E({Si(OH)}_{4}) \right]-[E(^{48-n}{Si}_{nH}^{n}{Al}_{zeo})+E({Al\left( OH \right)}_{3}\cdot H_{2}O)]$, where $E\left( {Al\left( OH \right)}_{3}\cdot H_{2}O \right)$ is the energy of the aqua complex of ${Al\left( OH \right)}_{3}$, $E({Si(OH)}_{4})$ is the energy of the formed ${Si(OH)}_{4}$, $E(^{48-n}{Si}_{nH}^{n}{Al}_{zeo})$ is the energy of the previous structure with nAl substitution, and $E(^{48-(n+1)}{Si}_{(n+1)H}^{n+1}{Al}_{zeo}$) is the energy of the zeolite structure with (n+1)Al substitution.

Due to the symmetry of the pure Si conventional cell (192T sites) of the FAU zeolite from the IZA database, the primitive cell (48T sites) is used for our study. Based on the pure Si zeolite, we gradually replaced 14 Si atoms by Al atoms and the Si/Al ratio ranges from 47 to 2.4, ranging from 1Al to 14Al.

**Table S1.** ENMAX and ENMIN values of the PAW potentials.

| Element | ENMAX (eV) | ENMIN (eV) |
| --- | --- | --- |
| Si | 245.345 | 184.009 |
| Al | 240.300 | 180.225 |
| O | 400.000 | 300.000 |
| Na | 101.968 | 76.476 |

**Table S2.** Total energies (E_tot-cutoff energy_) and energy differences *∆E*_cutoff energy_ (eV) of configurations in Figures 1-3 (cutoff energy = 500 or 550 eV).

| Configuration | *E*_tot_*_-_*_500_ (eV) | *∆E_500_* (eV) | *E*_tot_*_-_*_550_ (eV) | *∆E*_550_ (eV) |
| --- | --- | --- | --- | --- |
| Figure1-T1-4MR | -1147.059 | 0.21 | -1147.152 | 0.21 |
| Figure1-T1-6MR | -1147.270 | 0.00 | -1147.361 | 0.00 |
| Figure1-T1-D6R | -1147.114 | 0.16 | -1147.207 | 0.16 |
| Substitution enthalpy (∆*H*) in Figure 1 | | | | |
|  | Site | E_tot_ (eV) | ZPE (eV) | ∆*H* |
| 0 Al | - | -1145.50 | 10.12 | - |
| Figure1-T1-4MR | T1-6MR | -1147.22 | 10.06 | -0.91 |
| Figure1-T1-6MR | T1-4MR | -1147.05 | 10.11 | -0.69 |
| Figure1-T1-D6R | T1-D6R | -1147.11 | 10.09 | -0.77 |
|  |  |  |  |  |
| Configuration | *E*_tot_*_-_*_500_ (eV) | *∆E_500_* (eV) | *E*_tot_*_-_*_550_ (eV) | *∆E*_550_ (eV) |
| Figure2-E1-D6R | -1149.412 | 0.08 | -1149.503 | 0.08 |
| Figure2-E8-6MR | -1149.489 | 0.00 | -1149.579 | 0.00 |
| Figure2-F1-D6R | -1149.350 | 0.14 | -1149.443 | 0.14 |
| Figure2-F8-6MR | -1149.239 | 0.25 | -1149.3292 | 0.25 |
|  |  |  |  |  |
| Configuration | *E*_tot_*_-_*_500_ (eV) | *∆E_500_* (eV) | *E*_tot_*_-_*_550_ (eV) | *∆E*_550_ (eV) |
| Figure3-E10-D6R | -1151.672 | 0.05 | -1151.766 | 0.05 |
| Figure3-E11-D6R | -1151.721 | 0.00 | -1151.815 | 0.00 |
| Figure3-E6-D6R | -1151.484 | 0.24 | -1151.577 | 0.24 |
| Figure3-T3-D6R | -1151.493 | 0.23 | -1151.585 | 0.23 |
| Figure3-T5-D6R | -1151.720 | 0.00 | -1151.814 | 0.00 |
| Figure3-T6-D6R | -1151.670 | 0.05 | -1151.764 | 0.05 |


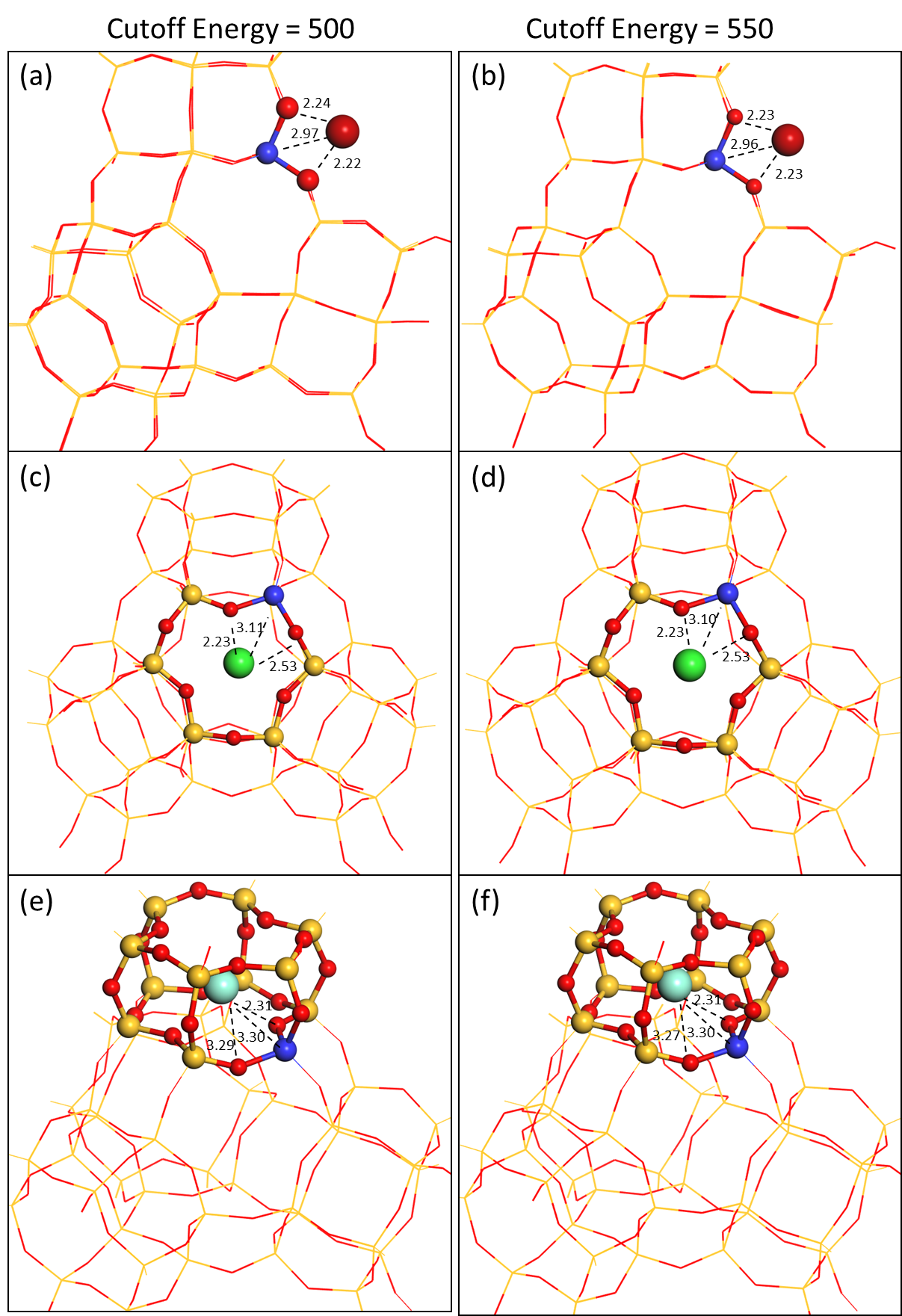


**Figure S1.** Structural comparation of configurations in Figure 1 optimized at different plane-wave cutoff energies.

**Table S3.** Specific data of configurations in Figure 1-3.

| Configuration | E_tot_ (eV) | ZPE (eV) | *∆E* (eV) | *∆E_rel_* (eV) |
| --- | --- | --- | --- | --- |
| Figure1-T1-4MR | -1147.05 | 14.79 | -1132.26 | 0.25 |
| Figure1-T1-6MR | -1147.25 | 14.74 | -1132.51 | 0.00 |
| Figure1-T1-D6R | -1147.10 | 14.75 | -1132.35 | 0.16 |
|  |  |  |  |  |
| Figure2-E1-D6R | -1149.41 | 14.73 | -1134.68 | 0.08 |
| Figure2-E8-6MR | -1149.49 | 14.74 | -1134.75 | 0.00 |
| Figure2-F1-D6R | -1149.35 | 14.75 | -1134.60 | 0.14 |
| Figure2-F8-6MR | -1149.24 | 14.74 | -1134.50 | 0.26 |
|  |  |  |  |  |
| Figure3-E10-D6R | -1151.67 | 14.74 | -1136.93 | 0.05 |
| Figure3-E11-D6R | -1151.72 | 14.74 | -1136.98 | 0.00 |
| Figure3-E6-D6R | -1151.48 | 14.75 | -1136.73 | 0.23 |
| Figure3-T3-D6R | -1151.49 | 14.75 | -1136.74 | 0.23 |
| Figure3-T5-D6R | -1151.71 | 14.74 | -1136.97 | 0.00 |
| Figure3-T6-D6R | -1151.67 | 14.77 | -1136.90 | 0.05 |

**Table S4.** Specific data of ZPE-corrected pyridine adsorption energy (*∆E*_ads_) on 4Na-10H-Y1. E (4Na-10H-Y1) = -1172.58 eV and the corresponding ZEP is 16.92 eV. E (Pyridine) = -71.20 eV, and the corresponding ZEP is 2.35 eV.

| Acid Sites | E_tot_ (eV) | ZPE (eV) | *∆E*_ads_ (eV) |
| --- | --- | --- | --- |
| T1 | -1246.15 | 19.67 | -1.97 |
| D4 | -1245.97 | 19.39 | -2.06 |
| T9 | -1245.89 | 19.63 | -1.74 |
| E2 | -1245.54 | 19.70 | -1.32 |
| F4 | -1245.48 | 19.27 | -1.69 |
| F6 | -1245.57 | 19.39 | -1.67 |
| E4 | -1245.47 | 19.70 | -1.26 |
| D10 | -1245.39 | 19.68 | -1.20 |
| F9 | -1245.34 | 19.39 | -1.45 |
| E8 | -1245.23 | 19.61 | -1.11 |

**Table S5.** Specific data of ZPE-corrected pyridine adsorption energy (*∆E*_ads_) on 14H-Y1. E(14H-Y1) = -1171.10 eV and the corresponding ZEP is 17.97 eV. E (Pyridine) = -71.20 eV, and the corresponding ZEP is 2.35 eV.

| Acid Sites | E_tot_ (eV) | ZPE (eV) | *∆E*_ads_ (eV) |
| --- | --- | --- | --- |
| T1 | -1244.23 | 20.47 | -1.77 |
| T5-D6R | -1244.18 | 20.42 | -1.78 |
| T9 | -1243.93 | 20.35 | -1.59 |
| F6 | -1243.95 | 20.42 | -1.55 |
| D4 | -1243.91 | 20.38 | -1.55 |
| E2 | -1243.92 | 20.37 | -1.56 |
| F4 | -1243.82 | 20.40 | -1.43 |
| D8-D6R | -1243.84 | 20.43 | -1.43 |
| F9 | -1243.82 | 20.42 | -1.42 |
| E4 | -1243.82 | 20.36 | -1.48 |
| E6-D6R | -1243.62 | 20.35 | -1.29 |
| D10 | -1243.62 | 20.41 | -1.23 |
| E8 | -1243.56 | 20.32 | -1.25 |
| F1-D6R | -1243.51 | 20.34 | -1.19 |

**^23^Na NMR Calculation:** To calculate the ^23^Na NMR, we adopted the same procedure and methods reported in a previous study.^[23]^ The ^23^Na NMR calculations were performed using a cluster model (Na-Y1-cluster, Figure S7a) derived from the fully optimized periodic Na-Y1 structure containing 14 framework Al atoms and 14 Na^+^ ions (Figure 7a), which was optimized by VASP.

The cluster model was constructed to preserve the local coordination environments of Na^+^ ions in sodalite cages, double six-ring units, and four-membered rings, with the Al-Na charge-compensation environment associated with the sodalite cage retained. Accordingly, the eight framework Al atoms associated with the sodalite cage, together with their charge-compensating Na^+^ ions, were explicitly retained in the Na-Y1-cluster model.

Framework Al atoms located outside the sodalite cage that could not be maintained with complete coordination in the finite cluster were replaced by Si to preserve structural integrity, and the corresponding Na^+^ ions were removed to maintain charge neutrality.

The resulting terminal oxygen atoms were saturated with hydrogen atoms to complete the cluster model.

To preserve the local geometries and Na^+^ positions defined by the periodic optimization, no further geometry optimization was performed at the cluster level. Single-point NMR calculations were carried out under structural constraints applied at the cluster boundaries.

The NMR shielding constants were calculated using the gauge-independent atomic orbital (GIAO) method^[17]^ as implemented in the Gaussian 16 package.^[18]^ The calculations were performed using the B3LYP hybrid exchange-correlation functional^[19-21]^ and the pcS-n basis sets of Jensen^[22]^ with pcS-4 for the Al atoms and pcS-1 for all other atoms. The ^23^Na chemical shifts were referenced to solid NaCl.^[24]^

The ^23^Na chemical shifts were obtained from the calculated isotropic shielding constants according to

$$\delta\left( {}^{23}{Na} \right)=\sigma_{ref}-\sigma_{calc}$$

where $\sigma_{calc}$​ is the computed isotropic shielding constant of Na in Na-Y1-cluster and $\sigma_{ref}$ is the corresponding shielding of the reference compound (solid NaCl).

**Ab Initio Molecular Dynamics (AIMD) Simulations**: All periodic Ab initio molecular dynamics (AIMD) simulations were performed in the canonical NVT ensemble and carried out using VASP with GGA-PBE. The temperature of simulated systems is maintained at 353 K employing the Andersen thermostat.^[5]^ We integrated the equations of motion with a time step of one femtosecond (fs) and each system is equilibrated for a time scale of two picoseconds (ps), followed by a production run of ten ps.

**Thermal Desorption**: The desorption temperature of NH_3_ and pyridine were estimated using the Redhead equation^[6]^, under the assumption that the pre-exponential factor $A$ is independent of surface coverage. This assumption holds for zeolites, as each Brønsted acid site typically adsorbs one probe molecule. The desorption rate as a function of coverage $\theta$ (in monolayers, ML) is given in equation 1 (Eq. 1), where $r\left( \theta\right)$ is the desorption rate [mol·(cm^2^·s)^-1^ ] as a function of coverage ($\theta$, ML); $A$ is the pre-exponential factor [s^-1^]; $E_{a}$ is the coverage-dependent activation energy for desorption [kJ·mol^-1^], $R$ is the gas constant [J·(K·mol)^-1^]; $T$ is the ab­solute temperature *K* and $n$ is the desorption order.

$r\left( \theta\right)=-\frac{d\theta}{dt}=A\theta^{n}exp\left( -\frac{E_{a}\left( \theta\right)}{RT} \right)$ Eq. 1

Under a constant heating rate, $\beta=\frac{dT}{dt}$, Eq. 1 can be transformed to equation 2 (Eq.2). In this study, the heating rate was set to 2K/min, in alignment with the experimental conditions used in NH_3_-TPD measurements.

$-\frac{d\theta}{dT}=\left( \frac{A}{\beta} \right)\theta^{n}exp\left( -\frac{E_{a}\left( \theta\right)}{RT} \right)$ Eq.2

The desorption activation energies were taken from the DFT-calculated static adsorption energies of the corresponding adsorption sites. A site-independent pre-exponential factor was employed for all adsorption sites of a given probe molecule, i.e., 10^11^ s^-1^ for NH_3_ desorption and 10^14^ s^-1^ for pyridine desorption, reflecting their different degrees of freedom. The resulting desorption temperatures and corresponding adsorption energies are summarized in Table S6.

**Table S6.** Desorption parameters for NH_3_ and pyridine derived from DFT adsorption energies.

| Adsorption Site (NH_3_) | Adsorption Energy (eV) | Desorption Temperature (K) |
| --- | --- | --- |
| F9 | 1.01 | 375 |
| E2 | 1.03 | 383 |
| F4 | 1.03 | 383 |
| D4 | 1.16 | 430 |
| T9 | 1.20 | 445 |
| D10 | 1.20 | 445 |
| E4 | 1.75 | 640 |
| T1 | 1.83 | 668 |
| F6 | 1.99 | 725 |
| Adsorption Site (Pyridine) | Adsorption Energy (eV) | Desorption Temperature (K) |
| E8 | 1.11 | 340 |
| D10 | 1.20 | 367 |
| E4 | 1.26 | 385 |
| E2 | 1.32 | 403 |
| F9 | 1.45 | 442 |
| F6 | 1.67 | 507 |
| F4 | 1.69 | 513 |
| T9 | 1.74 | 528 |
| T1 | 1.97 | 596 |
| D4 | 2.06 | 622 |

**Machine Learning (ML) Details:**

We generated structural descriptors capturing local chemical environments and topology for structur­al descriptors dependent on interatomic distances, computed rigorously by employing periodic boundary conditions to represent the infinite crystalline environment accurately. To handle interatomic distances within the periodic system and to avoid over­estimation due to artificial cell boundaries, the Minimum Image Convention (MIC) was consistently applied (Eq. 3), where $\vec{R}_{i}$ and $\vec{R}_{j}$ denote atomic positions within the simulation cell, $\vec{a}_{1}$, $\vec{a}_{2}$, $\vec{a}_{3}$ represent lattice vectors, and $\vec{n}=(n_{1}, n_{2}, n_{3})$are integer vectors indexing periodic cell images. This ensures that the shortest possible vector connecting atom pairs across periodic boundaries is always used for descriptor calculations. Additionally, atom positions were “unwrapped” from periodic images back into their continuous trajectories by applying the relation (Eq. 4), where $\vec{r}_{0}$ is a chosen reference position and the $\mathrm{round}\left( \cdot\right)$ operation ensures atomic coordinates remaining consistently tracked throughout structural analyses, avoiding artificial dis­continuities arising from periodic boundary crossings.

$\left\| \vec{R}_{i}-\vec{R}_{j} \right\|_{\mathrm{MIC}}=\min_{\vec{n}\in Z^{3}} \left\| \vec{R}_{i}-\vec{R}_{j}+n_{1}\vec{a}_{1}+n_{2}\vec{a}_{2}+n_{3}\vec{a}_{3} \right\|$ Eq. 3

${\vec{r}_{i}}^{\mathrm{unwrapped}}=\vec{r}_{0}+\left( \vec{r}_{i}-\vec{r}_{0}-round\left( \vec{r}_{i}-\vec{r}_{0} \right) \right)$ Eq. 4

Several geometric descriptors have been used in our study. MinNaAl stands for minimum Na-Al distance (Å) within the structure. NaAroundAl6 stands for average number of Al atoms within a radius of 6 Å around each Na atom. BridgeAngMean and BridgeAngStd stand for mean and standard deviations of Al-O-Si bridge angles (°). TTDistMean and TTDistStd stand for mean and standard deviations of distances (Å) between adjacent tetrahedral sites (T = Al, Si). To enhance the capacity of the model to capture both relative and nonlinear effects across compositional regimes, rank-normalized and squared forms of selected geometric descriptors were introduced. Rank features (*_rank) allow consistent comparison across Al-count groups by encoding intra-group percentile positions, whereas squared features (*_2) enable the model to resolve nonlinear energetic penalties associated with geometric extremes (e.g., short-range Coulomb repulsion or angular distortion).

**Atomic Environment Descriptors (SOAP and ACSF)**: To capture the local geometric environments of Na^+^ and Al atoms within the zeolite framework, we employed the Smooth Overlap of Atomic Positions (SOAP) descriptor. ^7,8^ SOAP encodes the spatial distribution of neighboring atoms by expanding the local atomic density in a basis of radial functions ($n_{\max}=8$) and spherical harmonics up to order $l_{\max}=6$, within a cutoff radius of 5 Å under periodic boundary conditions. For the species set (Na, Al, Si, O), this parameterization yields a per-center vector of 3696 elements as returned by Dscribe.^[9]^ For each structure, SOAP descriptors were computed for all Na centers and all Al centers and then averaged within each set to obtain sample-level SOAP_Na and SOAP_Al representations. To control dimensionality, principal component analysis (PCA)^[10]^ was applied separately to SOAP_Na and SOAP_Al, retaining more than 95% of the cumulative variance; in our dataset this resulted in 2 principal components for SOAP_Na and 3 for SOAP_Al. These five components were used as SOAP descriptors in the classification model.

Atom-Centered Symmetry Functions (ACSF)^[11]^ descriptor was similarly computed and averaged separately for Na and Al atomic sites, using standard radial and angular ACSF parameter sets with a radial cutoff of 6 Å. Particularly, a radial G^2^ function with parameters $\eta$ = 1, $R_{s}$ = 1.5, 2.0, 2.5, 3.0 (Eq. 5), and the angular G^4^ functions used two sets (η, ζ, λ) = (1,1,1), (2,1,1).

The radial term (G^2^) for a center atom i is defined as:

$G_{i}^{2}\left( \eta,R_{s} \right)=\sum_{j\neq i} e^{-\eta\left( R_{\mathrm{ij}}-R_{s} \right)^{2}}f_{c}(R_{\mathrm{ij}})$ Eq. 5

The angular term (G^4^) is defined as:

$G_{i}^{4}\left( \eta, \zeta, \lambda\right)=2^{1-\zeta}\sum_{j\neq i} \sum_{k\neq i, j} {(1+\lambda\cos\theta_{ijk})}^{\zeta}e^{-\eta\left( {R_{\mathrm{ij}}}^{2}+{R_{ik}}^{2}+{R_{\mathrm{jk}}}^{2} \right)}f_{c}\left( R_{\mathrm{ij}} \right)f_{c}\left( R_{\mathrm{ik}} \right)f_{c}\left( R_{\mathrm{jk}} \right)$ Eq. 6

where $R_{\mathrm{ij}}$, $R_{\mathrm{ik}}$ and $R_{\mathrm{jk}}$ are the interatomic distance between atom $i$, $j$ and $k$, $\theta_{ijk}$ is the angle formed by $\vec{R_{\mathrm{ij}}}$ and $\vec{R_{\mathrm{ik}}}$ (with $i$as the center), and $f_{c}$ is a smooth cutoff function ensuring locality.

In our study, structures were classified as stable (positive class) if their energies fell within the first lowest 5 samples in each Al-count group, as thermodynamically most favorable. Due to the severe class imbalance between the stable and unstable struc­tures, we employed the borderline synthetic minority oversampling technique (BorderlineSMOTE) to synthetically oversample minority class examples in the training dataset, thus mitigating imbalance-related biases. Subsequently, stable and unstable structures were classified using an ensemble-learning strategy (VotingClassifier) combining three complementary classifiers: XGBoost (Extreme Gradient Boosting),^[12]^ Random Forest,^[13]^ and HistGradientBoosting.^[14,15]^ Each classifier was selected for its dis­tinct strengths, collectively enhancing predictive robustness and accuracy.

Specifically, XGBoost is a gradient-boosting ensemble algorithm that builds sequential decision trees, each designed to minimize residual errors from previous iterations. Formally, XGBoost optimizes a regularized objective function (Eq. 7), where $l\left( y_{i},{\hat{y}_{i}}^{(t)} \right),$ denotes the chosen loss function (e.g., logistic loss for binary classification), ${\hat{y}_{i}}^{(t)}$ is the predicted value for sample $i$ at iteration $t$. The regularization term $\Omega\left( f_{k} \right)$ explicitly controls model complexity, reducing the risk of overfitting (Eq. 8), where $T_{k}$ denotes the number of leaves in the $k$th decision tree, and $w_{k}$ are the leaf weights. Hyperparameters were carefully optimized and set as follows: number of decision trees (estimators) = 200, maximum depth of each tree = 5, learning rate = 0.05, data sub­sampling ratio per tree = 0.9, and both sample subsampling and feature subsampling ratios per tree set to 0.9. To address the severe class imbalance explicitly, the class weighting parameter (scale_-_pos_-_weight =N_negative_/N_positive_) was introduced, ensuring balanced training of minority and majority classes.

$L\left( \Phi\right)=\sum_{i}^{N} l\left( y_{i},{\hat{y}_{i}}^{(t)} \right)+\sum_{k=1}^{N} \Omega\left( f_{k} \right)$ Eq. 7

$\Omega\left( f_{k} \right)=\gamma T_{k}+\frac{1}{2}\lambda\left\| w_{k} \right\|^{2}$ Eq. 8

**Random Forest** (**RF**) is a versatile ensemble learning method characterized by constructing an extensive collection of decision trees. Each tree in the ensemble is independently trained on a random bootstrap subset of the dataset, supplemented by randomized selection of input features at each decision split, thereby introducing intrinsic diversity among the trees. The final classification decision is determined by aggregating individual tree predictions through a majority-voting mechanism (Eq. 9). where $h_{t}\left( x \right)$ is the predicted class label from the $t$th decision tree, and $\boldsymbol{I(\cdot)}$ represents the indicator function. To systematically address class imbalance, each class was assigned balanced weights during training, proportional inversely to the number of instances in each class (Eq. 10), where $N$ is the total number of training samples, $N_{c}$ is the number of samples in class $c$, and $C$ is the total number of classes. This weighting strategy ensures equitable contribution of minority classes, improving classifier sensitivity.

$\hat{y}_{i}=\mathrm{argmax}_{c}\sum_{t=1}^{T} \mathbf{I}(h_{t}\left( x \right)=c)$ Eq. 9

$\mathrm{weight}_{c}=\frac{N}{C\cdot N_{c}}$ Eq. 10

HistGradientBoosting is an advanced implementation of gradient boosting optimized specifically for efficient handling of large datasets. In principle, HistGradientBoosting sequentially constructs regression trees to iteratively refine model predictions, where each new tree aims to minimize residual errors, formally known as pseudo-residuals, from the preceding ensemble (Eq. 11), where $F_{m}\left( x \right)$ is the aggregated model at iteration $m$, $h_{m}(x)$ is the regression tree trained on residual gradients, and $v$ (learning rate) controls the incremental contribution from each subsequent tree.

$F_{m}\left( x \right)=F_{m-1}\left( x \right)+vh_{m}(x)$ Eq. 11

**Model Evaluation Metrics and Threshold Optimization:** Model performance was evaluated using stratified 10-fold cross-validation, ensuring class proportion consistency across all folds. To assess classification quality comprehensively, we employed a combination of threshold-independent and threshold-sensitive metrics. The receiver operating characteristic (ROC) curve, which plots the true positive rate (TPR) against the false positive rate (FPR), was used to evaluate the global discriminative capacity of a model. The corresponding area under the curve (AUC) provides a scalar summary, with values approaching unity indicating near-perfect class separation.

Given the pronounced class imbalance in our dataset, additional emphasis was placed on the precision-recall (PR) curve, which offers more informative insight in scenarios where one class dominates. Here, precision (the fraction of true positives among predicted positives) and recall (also known as sensitivity) are jointly considered. Their harmonic mean, the$F_{1}$ score, was used to guide threshold selection (Eq. 12). An optimal classification threshold was identified by maximizing the $F_{1}$ score on the validation set, striking a balance between minimizing false negatives and false positives.

$F_{1}=2\cdot\frac{Precision\cdot Recall}{Precision+Recall}$ Eq. 12

**Model Interpretation Feature Importance and Model Interpretability:** To interpret the model and quantify the relative im­portance of each descriptor, we employed SHapley Additive exPlanations analysis (SHAP)^[16]^ based on the XGBoost component of the ensemble. Rooted in cooperative game theory, SHAP assigns each feature an attribution value that reflects its marginal contribution to the model’s output across all possible feature combinations. The predicted probability $f\left( x \right)$ for a given sample $x$ can be decomposed (Eq. 13), where $\Phi_{0}$ denotes the expected model output (i.e., the base value across the training distribution), and $\Phi_{i}$ represents the SHAP value for feature $x_{i}$, quantifying how much that descriptor shifts the prediction away from the baseline. This additive decomposition offers a consistent and locally accurate explanation of model behavior at the individual sample level.

$f\left( x \right)=\Phi_{0}+\sum_{i=1}^{M} \Phi_{i}x_{i}$ Eq. 13


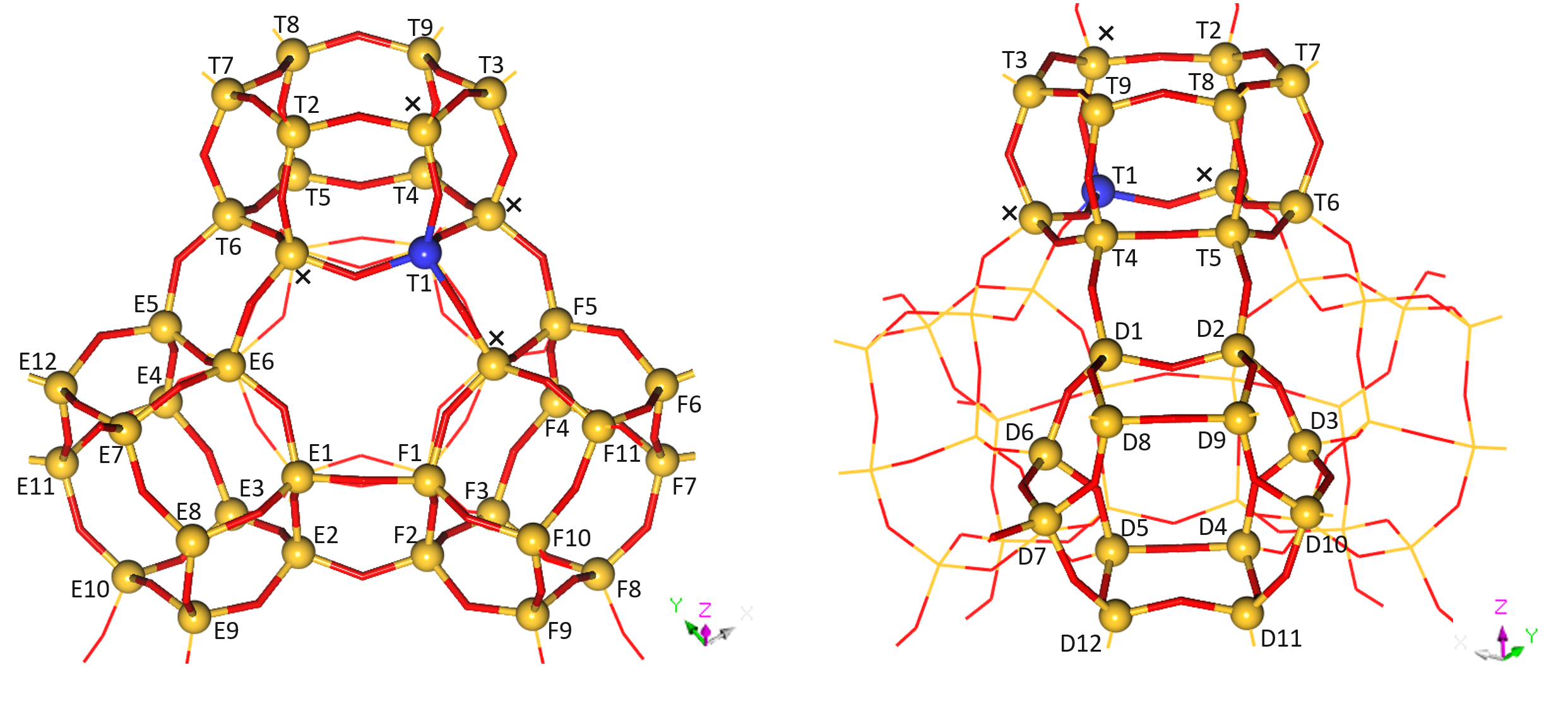


**Figure S2.** Specific framework T‒sites labeled at E, F, D and T zones.


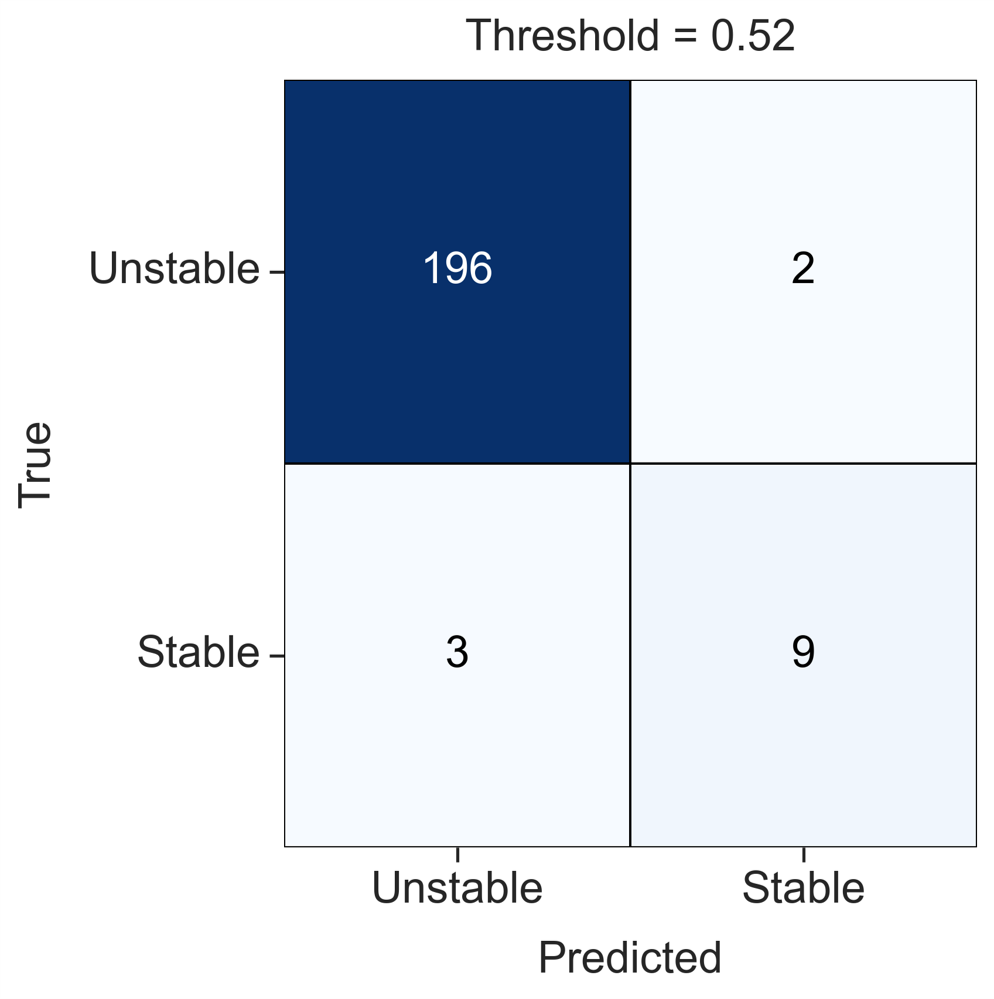


**Figure S3.** Model precision and recall.


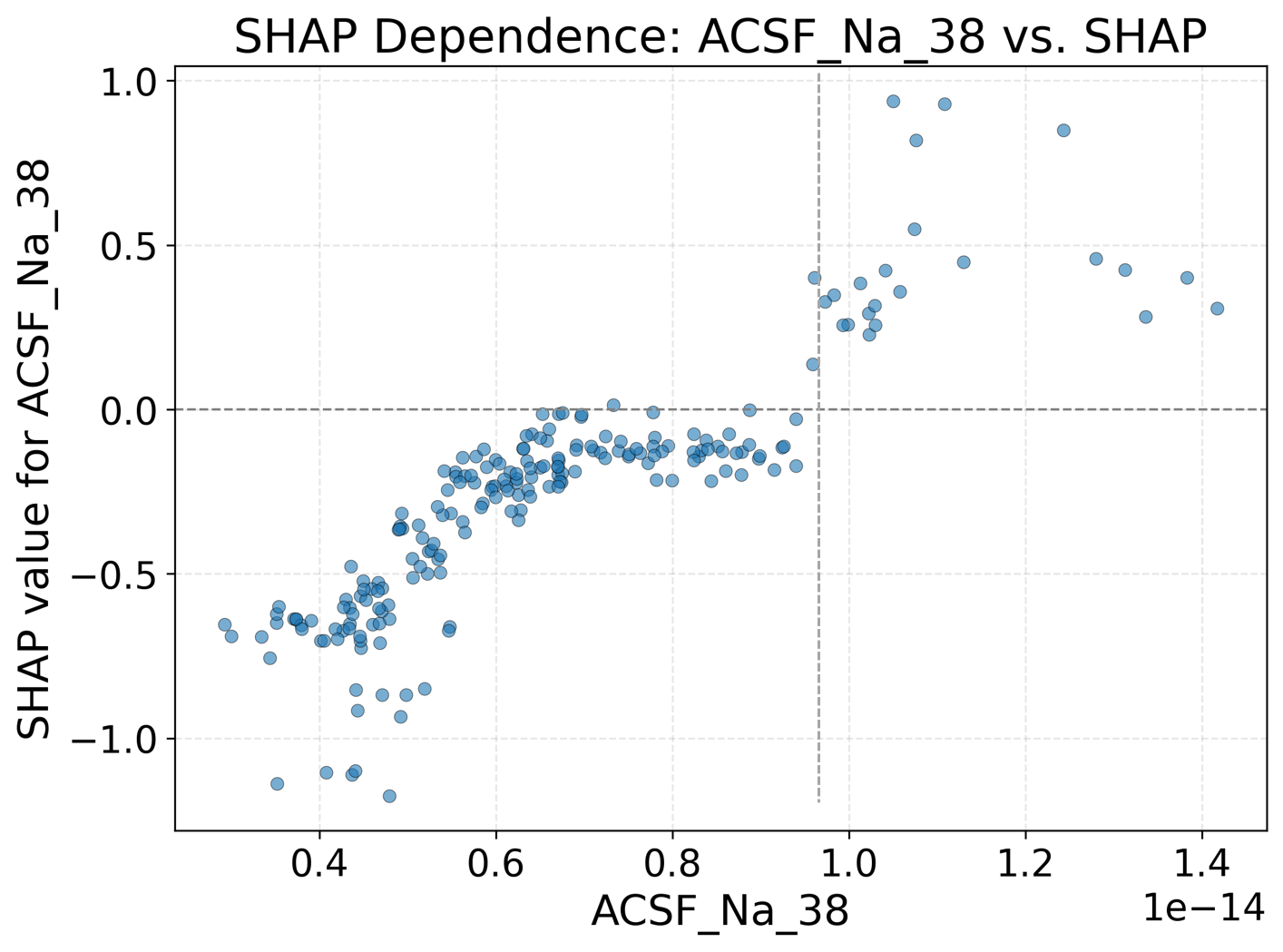


**Figure S4.** SHAP dependence plot for the descriptor of ACSF_Na_38.


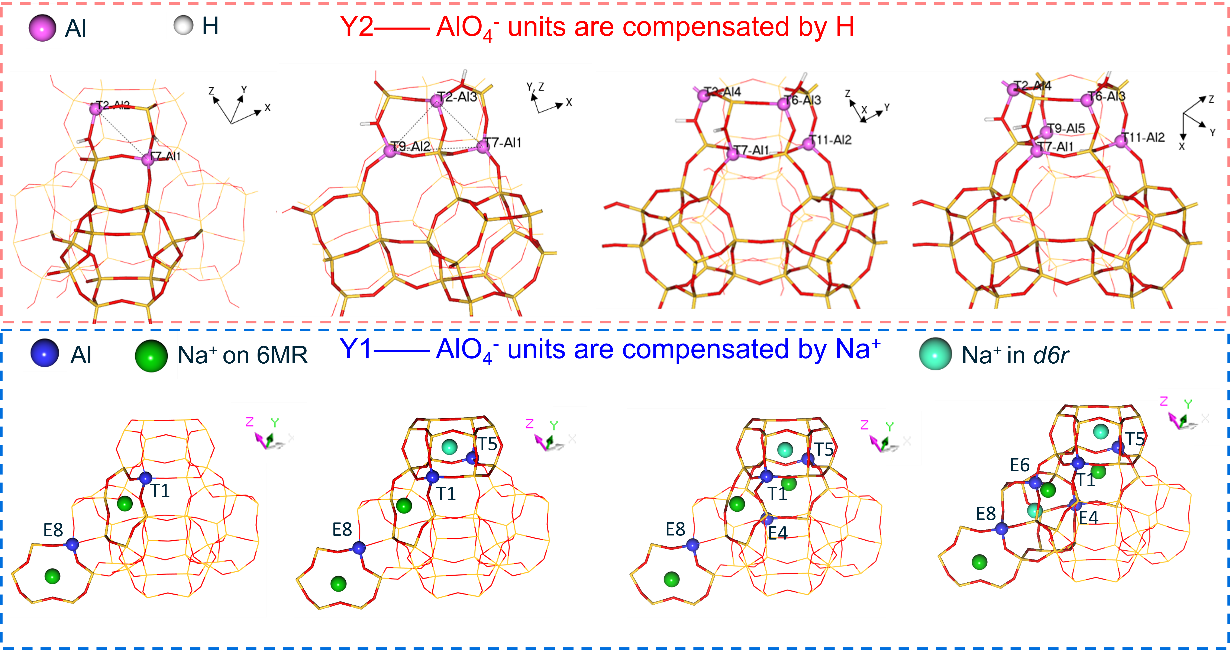


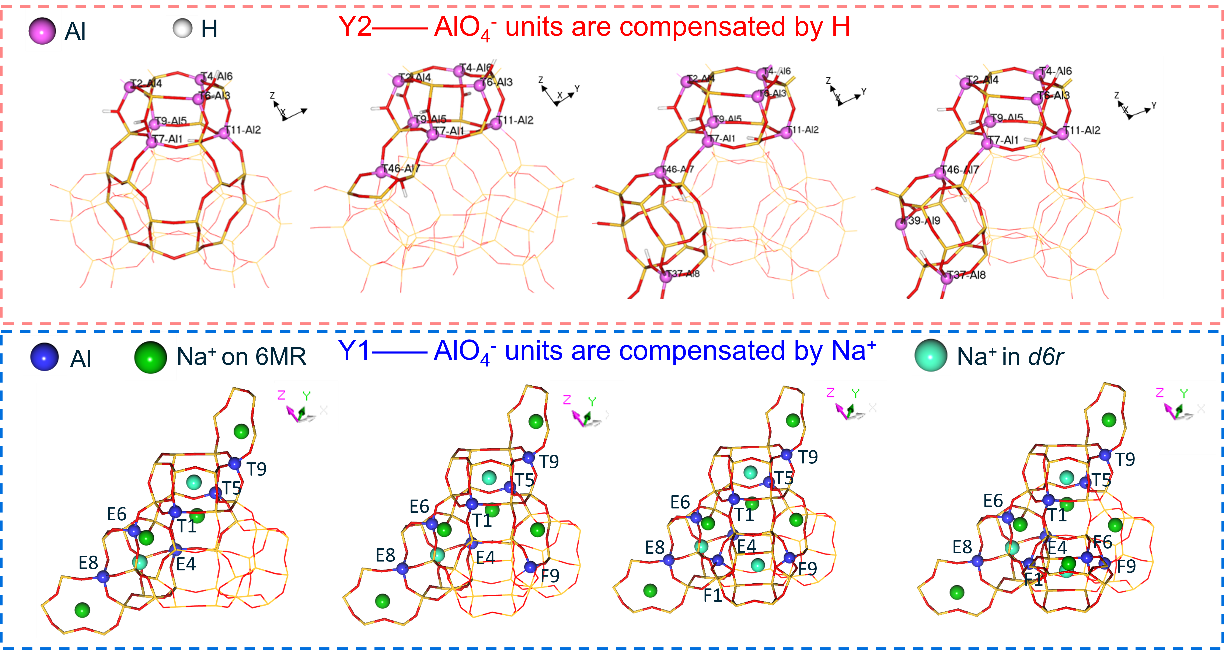


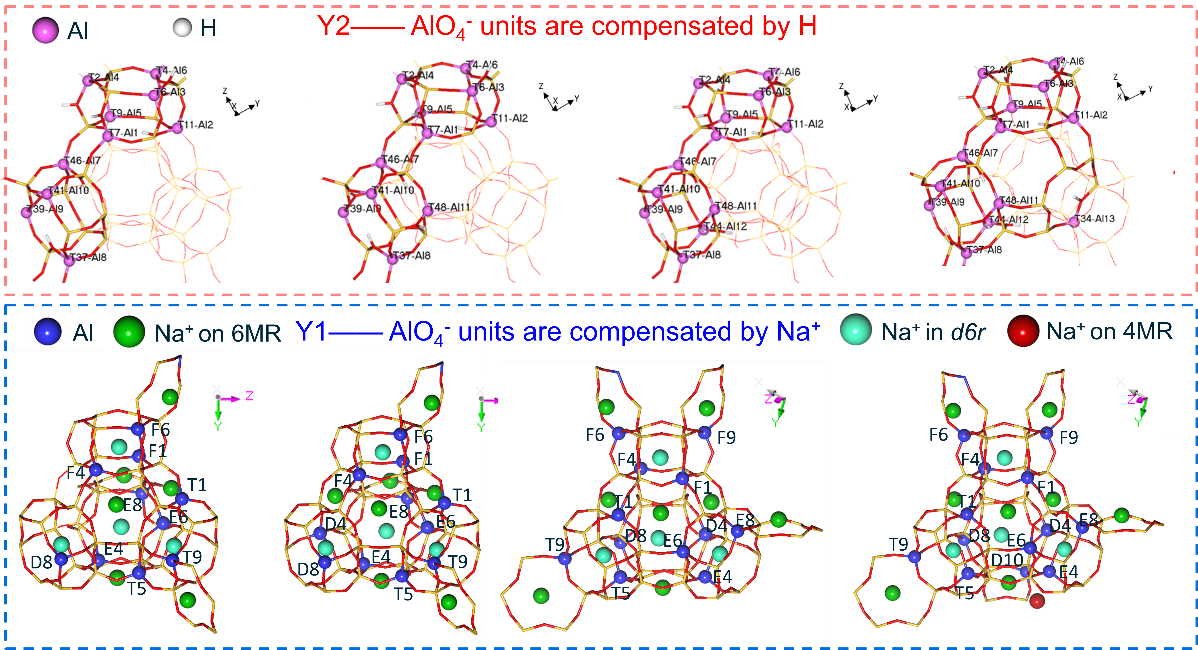


**Figure S5.** Al distribution and the corresponding Na^+^ positions on Y‒1 and Y‒2 (2 Al to 14 Al).


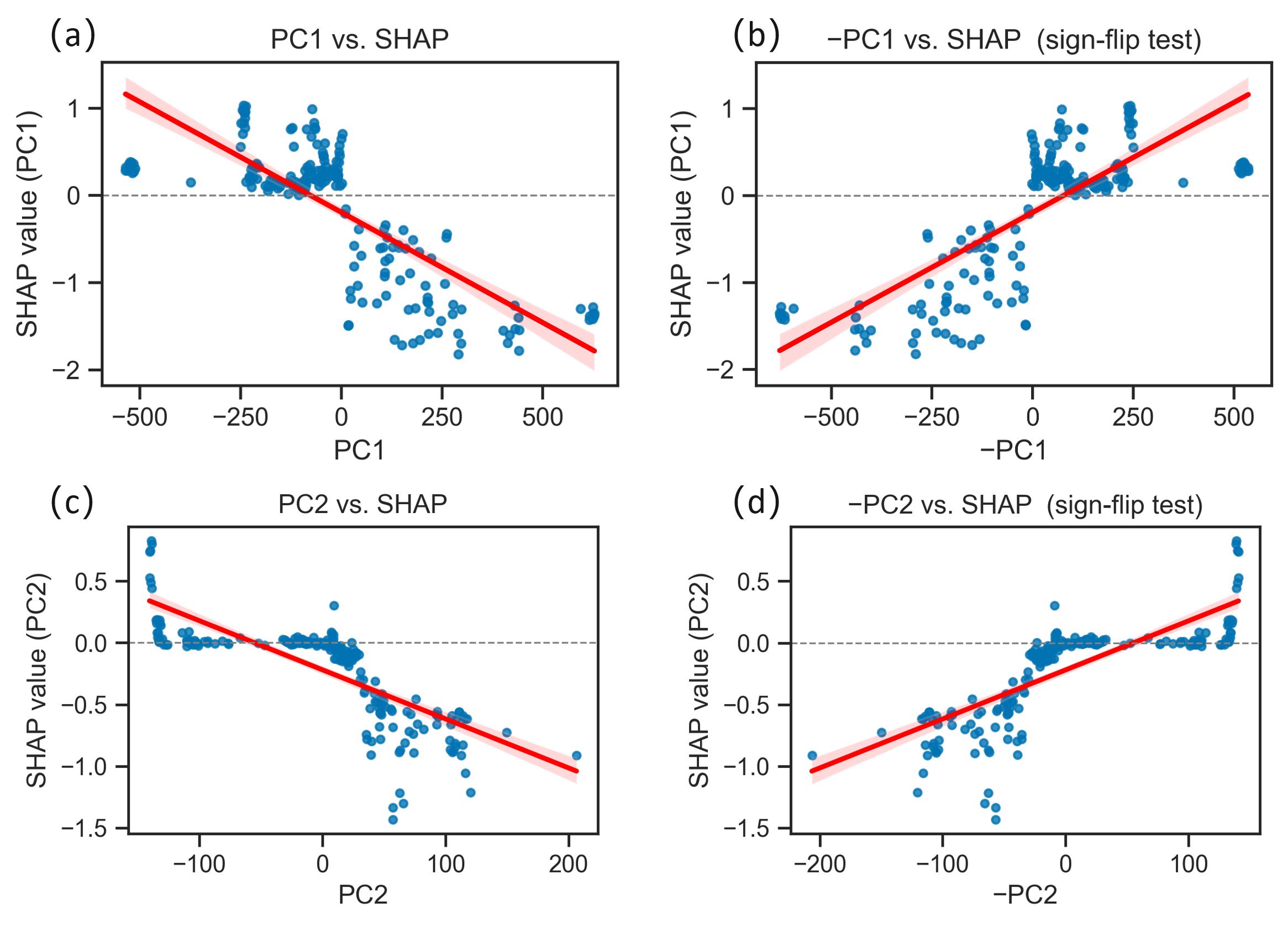


**Figure S6.** SHAP dependence plot for (a) SOAP_Na_PC1. (b) SOAP_Na_-PC1. (c) SOAP_Na_PC2. (d) SOAP_Na_-PC2.

Although SOAP_Na_PC1 and SOAP_Na_PC2 rank highly in our SHAP analysis, their sign‐dependent trends are artefacts of PCA’s inherent sign ambiguity. By definition, each principal axis satisfies $\sum v_{k}=\lambda_{k}v_{k}$, where $\sum$is the symmetric covariance matrix, $v_{k}$is the kth eigenvector and $\lambda_{k}$ is the corresponding eigenvalue. Since $v_{k}$ and ${-v}_{k}$ are equivalent solutions, flipping the sign of every projected score $z_{ik}=X_{i}\cdot v_{k}$ , where $X_{i}$ denotes the $i$th sample’s mean‐centered descriptor (feature) vector, leaves both the variance $\lambda_{k}$ and all pairwise Euclidean distances $\left\| z_{i}-z_{j} \right\|$ invariant, and preserves classifier metrics (accuracy, precision-recall, AUPRC); the only effect is to mirror the SHAP‐dependence plot across the vertical axis, inverting the slope without changing its magnitude. This unequivocally demonstrates that the sign of a principal component, and consequently the sign of its SHAP coefficient, holds no inherent physical meaning.

ACSF descriptors enable physical interpretation by explicitly encoding two-body (G_2_) and three-body (G_4_) geometric motifs, providing physically meaningful insights into zeolite framework stability. In the SHAP analysis, two of the most influential descriptors related to local atomic environments around Na^+^ are identified as ACSF_Na_38 and ACSF_Na_39. These descriptors correspond specifically to two Gaussian-angular G_4_ basis functions describing the O-Na-O three-body interaction, given by: $ACSF\_Na\_n\propto\left( 1+cos\theta\right)^{\xi}exp\left[ -\eta\left( r_{ij}^{2}+r_{ik}^{2}+r_{jk}^{2} \right) \right]f_{c}\left( r_{ij} \right)f_{c}\left( r_{ik} \right)f_{c}\left( r_{jk} \right)$,^[10]^ where $\theta$ is the O-Na-O bond angle, $r_{ij}$ and $r_{ik}$ are the Na-O distances, $r_{jk}$ is the O-O distance, $\eta$ and $\xi$are the parameterization constants ($\xi$ = 1 for ACSF_Na_38, $\xi$= 2 for ACSF_Na_39), and $f_{c}\left( r \right)$ is the smooth cutoff function with $r_{c}$=6  Å. Because all coordination distances in the Na-O-O structure lie within the cutoff radius, the exponential and cutoff factors can be approximated as a constant prefactor $K$. Under this approximation the descriptors reduce to $G_{4}\left( \theta\right)\approx{K\left( 1+cos\theta\right)}^{\xi}$, SHAP dependence plots reveal that both ACSF_Na_38 and ACSF_Na_39 increase the predicted stability when their values exceed characteristic thresholds ( about 8×10^−15^ for ACSF_Na_38 and 4×10^−28^ for ACSF_Na_39). Inverting the simplified functional forms, these thresholds correspond to $1+cos\theta\geq\frac{8\times{10}^{-15}}{K} \left( \xi= 1 \right)$ and $1+cos\theta\geq\sqrt{\frac{4\times{10}^{-28}}{K}} \left( \xi= 2 \right)$. Using the FAU framework parameters ($r_{ij}=r_{ik}\approx2.35 \mathbf{Å}$ , $r_{jk}\approx2.60 \mathbf{Å}$, $K\approx3\times{10}^{-8}$) in the $G_{4}$expression gives yields $cos\theta\approx-0.9999$ in both cases, which corresponds to an O-Na-O bond angle of approximately 179.9°. Consequently, the model strongly favors a nearly linear O-Na-O configuration. Physically, a linear O-Na-O arrangement maximizes electrostatic screening of the Na^+^ cation by the anionic aluminosilicate framework and minimizes local strain. ACSF_Na_38 and ACSF_Na_39 capture the primary increase in stability as the O-Na-O angle approaches 180°.


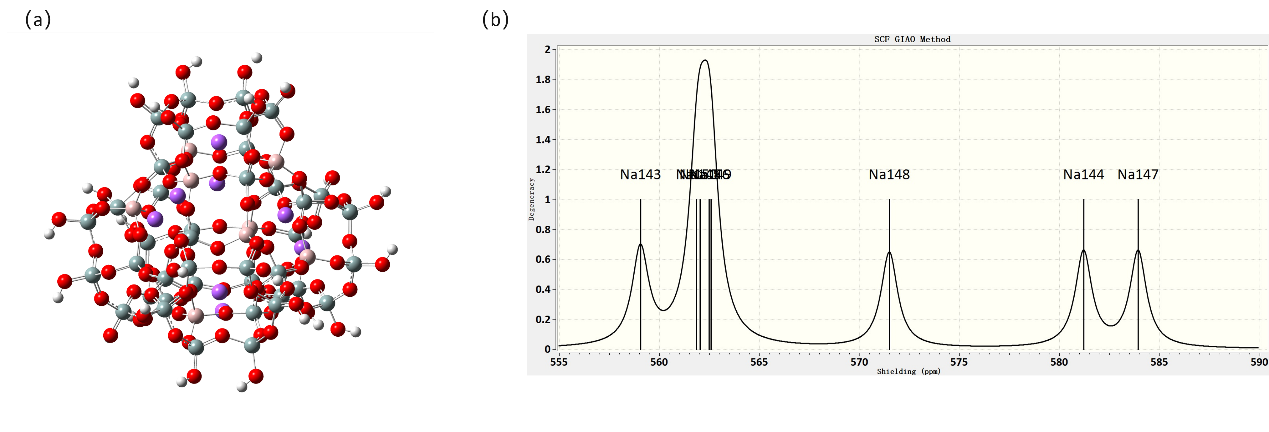


**Figure S7.** (a) Structural model of Na‒Y1‒cluster. Red, white, cyan, and pink spheres represent O, H, Si, and Al atoms, respectively. (b) Calculated isotropic chemical shifts for the eight Na^+^ ions in Na-Y1-cluster.


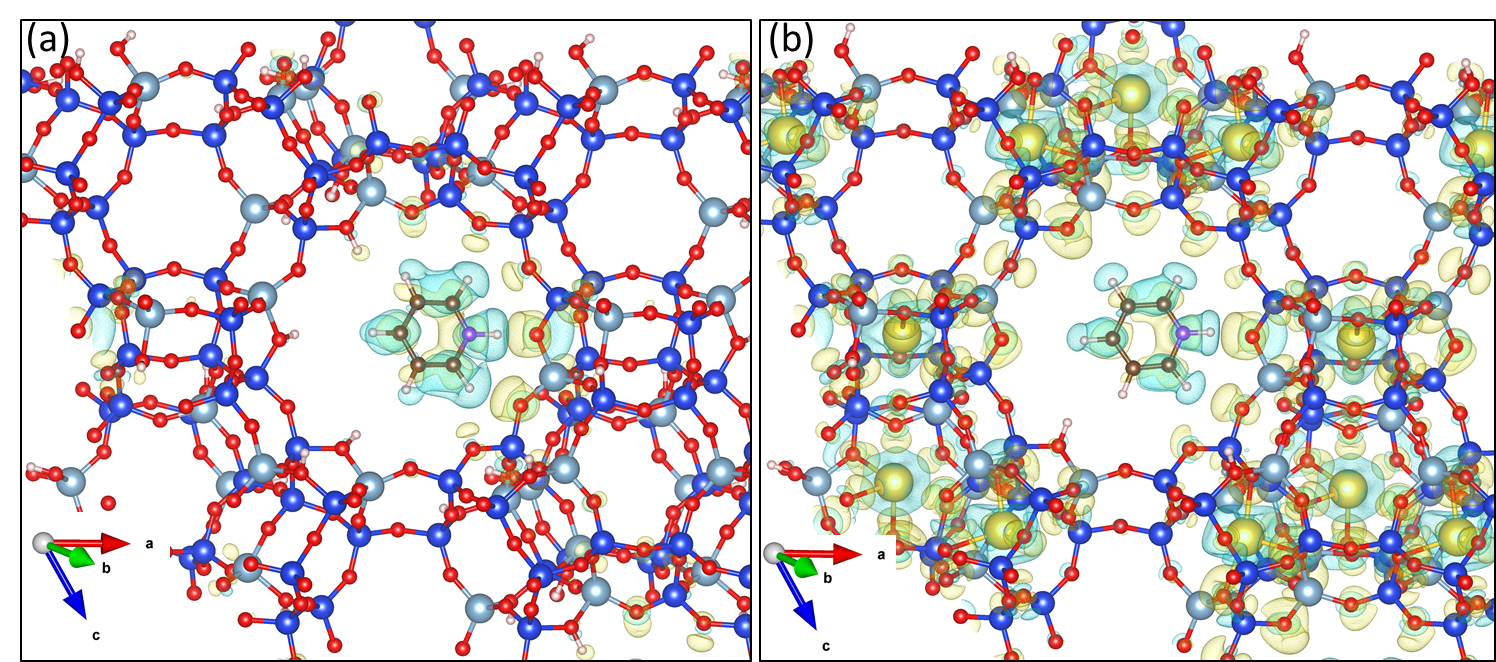


**Figure S8.** Charge-density-difference analysis of pyridine adsorbed on (a) 14H-Y1, (b) 4Na+10H-Y1. Red, white, blue, cyan, gray, purple and gold spheres represent O, H, Si, Al, C, N and Na atoms, respectively.

**
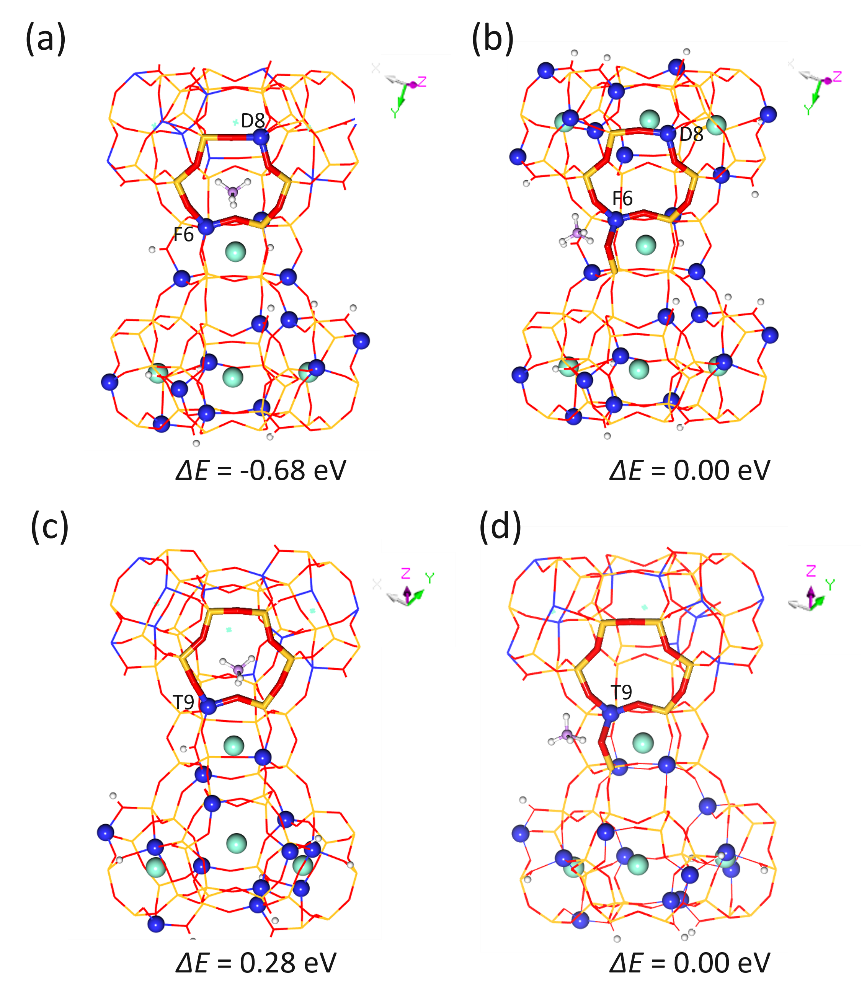
**

**Figure S9.** Configurations of NH_4_^+^ adsorbed on (a) F6-6MR, (b) F6-4MR, (c) T9-6MR and (d) T9-4MR in Table S23.

**Figure S10.** Py-IR of Na-H-Y.


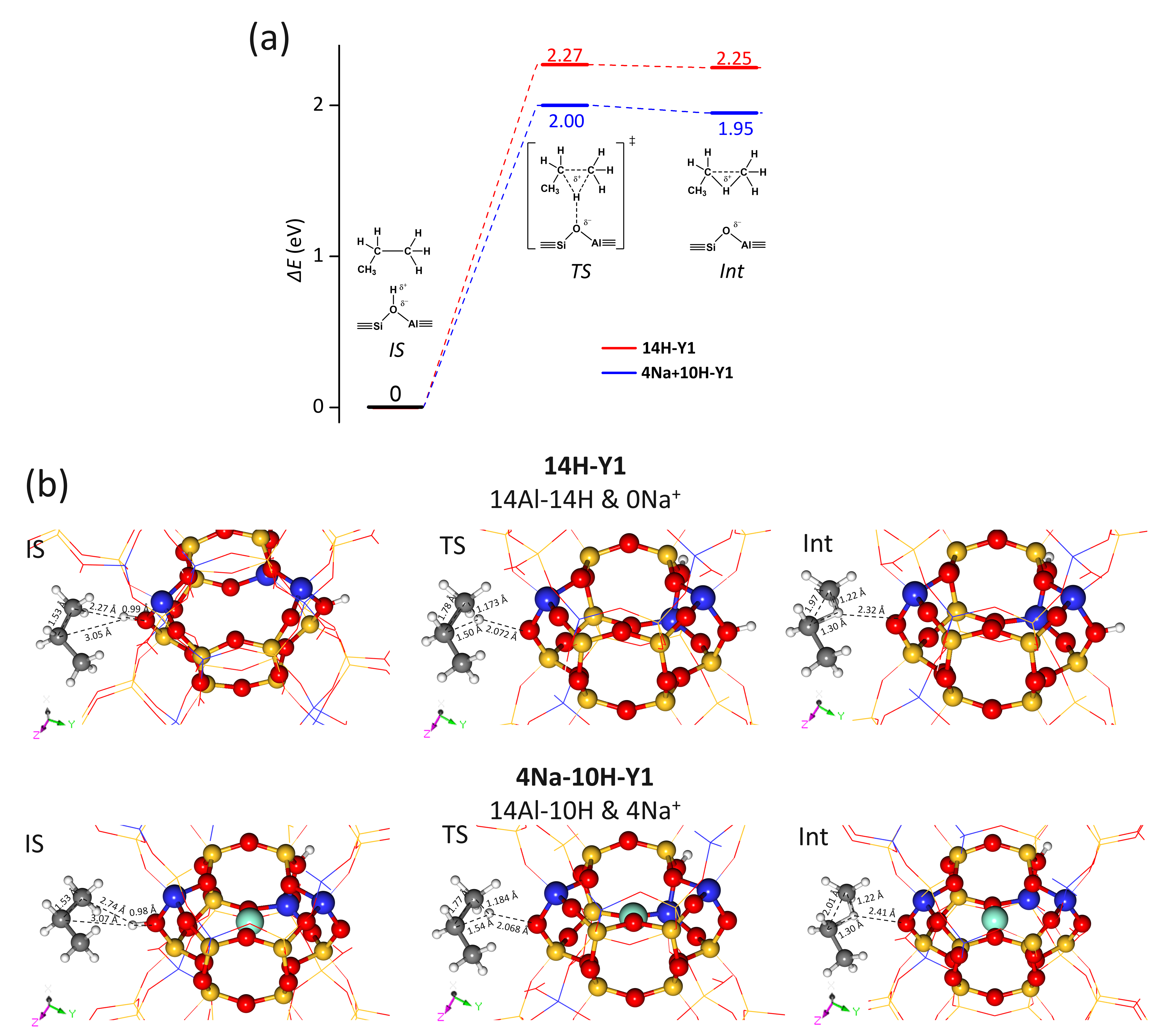


**Figure S11.** (a) Energy profiles of propane cracking. (b) Local structures of initial states (IS), transition states (TS), and reaction intermediates (Int) for propane cracking on 14H-Y1 and 4Na-10H-Y1.


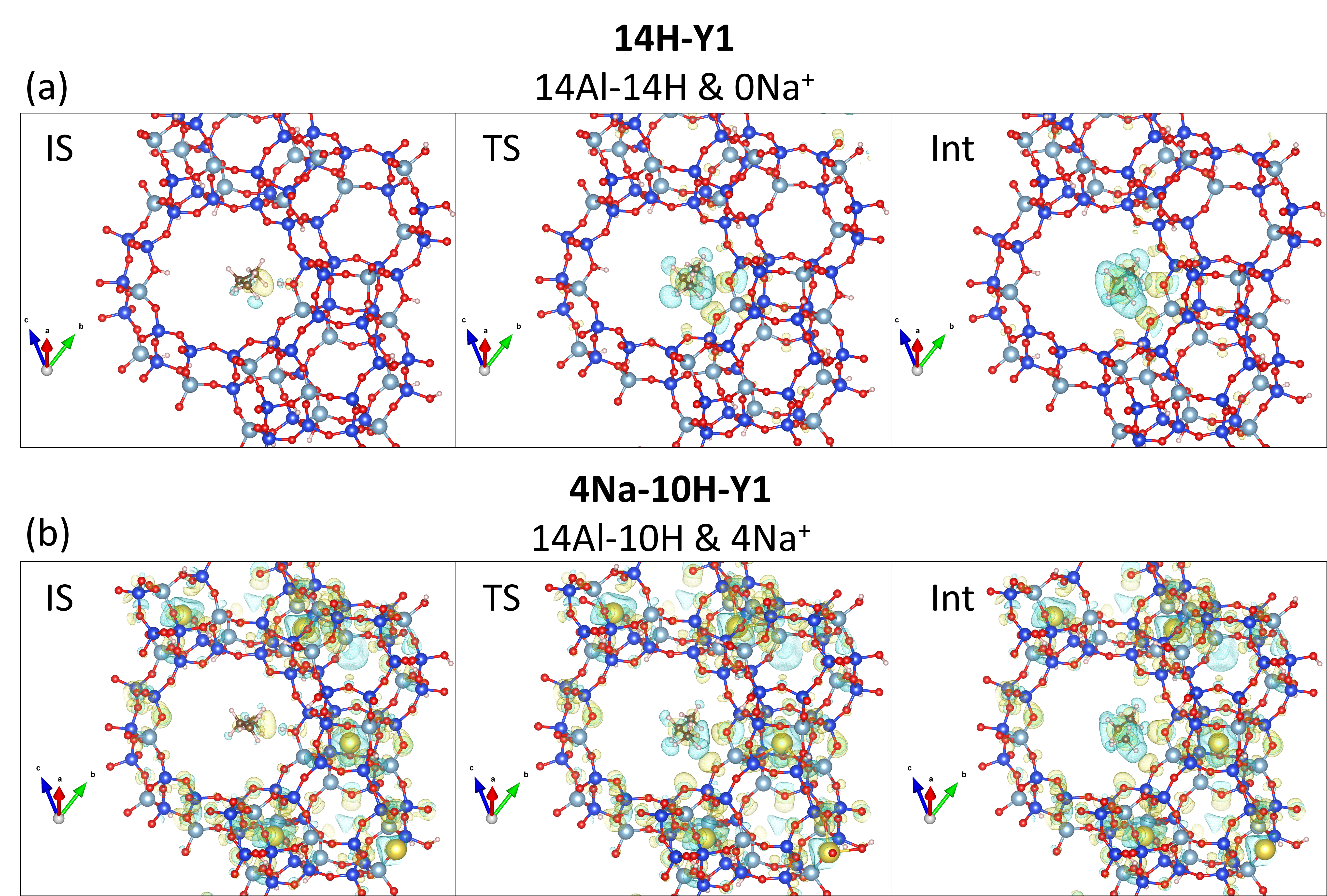


**Figure S12.** Charge-density-difference analysis of propane cracking for the initial state (IS), transition state (TS) and intermediate (Int) on (a) 14H-Y1 and (b) 4Na+10H-Y1. Red, white, blue, cyan, gray and gold spheres represent O, H, Si, Al, C and Na atoms, respectively.

| **Table S7**. Structural energies of Al distribution and corresponding Na^+^ positions on Y-zeolites  (Si/Al = 23, Si = 47, Al = 1) | | | | | | | |
| --- | --- | --- | --- | --- | --- | --- | --- |
| T zone | Energy (eV) | E zone | Energy (eV) | D zone | Energy (eV) | F zone | Energy (eV) |
| **T1-6MR** | **-1147.28** |  |  |  |  |  |  |
| T1-D6R | -1147.11 |  |  |  |  |  |  |
| T1-4MR | -1147.07 |  |  |  |  |  |  |
| **Table S8**. Structural energies of Al distribution and corresponding Na^+^ positions on Y-zeolites  (Si/Al = 23, Si = 46, Al = 2) | | | | | | | |
| Based on  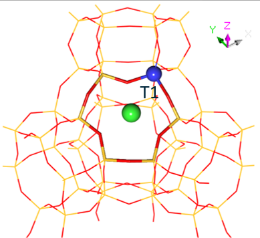  **T1-6MR** from Table S7 | | | | | | | |
| T zone | Energy (eV) | E zone | Energy (eV) | D zone | Energy (eV) | F zone | Energy (eV) |
| T5-D6R | -1149.31 | **E8-6MR** | **-1149.47** | D6-6MR | -1149.26 | F1-D6R | -1149.33 |
| T6-D6R | -1149.30 | E1-D6R | -1149.39 | D7-6MR | -1149.23 | F10-6MR | -1149.31 |
| T8-D6R | -1149.27 | E9-6MR | -1149.31 | D1-6MR | -1149.23 | F9-6MR | -1149.24 |
| T7-D6R | -1149.26 | E2-6MR | -1149.28 | D10-6MR | -1149.15 | F4-6MR | -1149.24 |
| T9-D6R | -1149.16 | E5-6MR | -1149.26 | D2-6MR | -1149.13 | F2-6MR | -1149.23 |
| T4-d6 | -1149.16 | E7-6MR | -1149.24 | D9-6MR | -1149.12 | F8-6MR | -1149.21 |
| T2-D6R | -1149.15 | E11-6MR | -1149.19 | D12-6MR | -1149.11 | F5-6MR | -1149.20 |
| T8-6MR | -1149.14 | E6-4MR | -1149.18 | D3-6MR | -1149.11 | F7-6MR | -1149.15 |
| T7-6MR | -1149.12 | E6-D6R | -1149.17 | D11-6MR | -1149.10 | F3-6MR | -1149.14 |
| T5-6MR | -1149.11 | E10-6MR | -1149.17 | D8-6MR | -1149.09 | F11-6MR | -1149.11 |
| T4-6MR | -1149.09 | E1-4MR | -1149.16 | D4-6MR | -1149.08 | F6-6MR | -1149.09 |
| T6-6MR | -1149.09 | E12-6MR | -1149.16 | D5-6MR | -1149.06 | F10-D6R | -1149.07 |
| T3-D6R | -1149.05 | E4-6MR | -1149.15 | D10-D6R | -1149.04 | F2-D6R | -1149.07 |
| T9-6MR | -1149.03 | E3-6MR | -1149.12 | D2-D6R | -1149.04 | F9-D6R | -1149.00 |
| T3-6MR | -1149.01 | E2-4MR | -1149.06 | D3-D6R | -1149.04 | F3-4MR | -1148.99 |
| T2-6MR | -1149.01 | E5-4MR | -1149.02 | D9-D6R | -1149.04 | F8D6R | -1148.98 |
| T5-4MR | -1148.91 | E8-D6R | -1149.02 | D7-D6R | -1149.03 | F2-4MR | -1148.98 |
| T8-4MR | -1148.88 | E2-D6R | -1149.00 | D4-D6R | -1149.03 | F3-D6R | -1148.97 |
| T7-4MR | -1148.88 | E9-4MR | -1149.00 | D6-D6R | -1149.02 | F9-4MR | -1148.96 |
| T2-4MR | -1148.87 | E12-4MR | -1148.96 | D8-D6R | -1149.02 | F5-4MR | -1148.96 |
| T9-4MR | -1148.84 | E3-4MR | -1148.95 | D5-D6R | -1149.01 | F6-4MR | -1148.96 |
| T6-4MR | -1148.84 | E10-4MR | -1148.94 | D1-D6R | -1148.98 | F8-4MR | -1148.95 |
| T3-4MR | -1148.84 | E5-D6R | -1148.94 | D11-D6R | -1148.96 | F1-4MR | -1148.94 |
| T4-4MR | -1148.81 | E7-D6R | -1148.94 | D5-4MR | -1148.95 | F11-D6R | -1148.94 |
|  |  | E4-4MR | -1148.91 | D12-4MR | -1148.93 | F7-D6R | -1148.93 |
|  |  | E11-4MR | -1148.91 | D2-4MR | -1148.93 | F6-D6R | -1148.93 |
|  |  | E12-D6R | -1148.91 | D4-4MR | -1148.91 | F11-4MR | -1148.92 |
|  |  | E11-D6R | -1148.89 | D1-4MR | -1148.91 | F4-D6R | -1148.91 |
|  |  | E7-4MR | -1148.89 | D9-4MR | -1148.90 | F5-4MR middle | -1148.89 |
|  |  | E8-4MR | -1148.89 | D6-4MR | -1148.89 | F7-4MR | -1148.89 |
|  |  | E9-D6R | -1148.87 | D3-4MR | -1148.88 | F4-4MR | -1148.88 |
|  |  | E4-D6R | -1148.85 | D11-4MR | -1148.86 | F10-4MR | -1148.88 |
|  |  | E3-D6R | -1148.85 | D8-4MR | -1148.83 | F5-D6R | -1148.84 |
|  |  | E10-D6R | -1148.85 | D10-4MR | -1148.81 |  |  |
|  |  |  |  | D12-D6R | -1148.80 |  |  |
|  |  |  |  | D7-4MR | -1148.77 |  |  |
| **Table S9**. Structural energies of Al distribution and corresponding Na^+^ positions on Y-zeolites  (Si/Al = 15, Si = 45, Al = 3) | | | | | | | |
| Based on  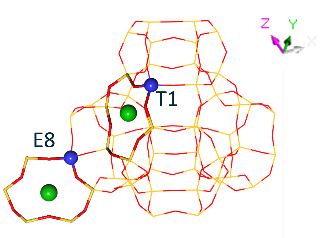  **E8-6MR** from Table S8  The first two sequences of Al:  T1-6MR→E8-6MR | | | | | | | |
| T zone | Energy (eV) | E zone | Energy (eV) | D zone | Energy (eV) | F zone | Energy (eV) |
| **T5-D6R** | **-1151.70** | **E11-D6R** | **-1151.70** | D9-6MR | -1151.45 | F4-6MR | -1151.45 |
| T6-D6R | -1151.65 | E10-D6R | -1151.65 | D5-6MR | -1151.44 | F11-6MR | -1151.44 |
| T3-D6R | -1151.47 | E6-D6R | -1151.47 | D6-6MR | -1151.44 | F10-6MR | -1151.44 |
| T2-D6R | -1151.39 | E2-D6R | -1151.39 | D12-D6R | -1151.43 | F1-D6R | -1151.43 |
| T8-6MR | -1151.38 | E4-6MR | -1151.38 | D3-6MR | -1151.42 | F8-6MR | -1151.41 |
| T5-6MR | -1151.38 | E11-6MR | -1151.37 | D11-6MR | -1151.41 | F2-6MR | -1151.41 |
| T2-6MR | -1151.37 | E2-6MR | -1151.36 | D1-6MR | -1151.40 | F6-6MR | -1151.39 |
| T8-D6R | -1151.36 | E4-D6R | -1151.36 | D4-6MR | -1151.39 | F9-6MR | -1151.39 |
| T9-6MR | -1151.36 | E5-6MR | -1151.36 | D2-6MR | -1151.37 | F3-6MR | -1151.37 |
| T7-D6R | -1151.35 | E3-D6R | -1151.34 | D8-6MR | -1151.36 | F7-6MR | -1151.37 |
| T9-D6R | -1151.34 | E5-D6R | -1151.34 | D10-6MR | -1151.36 | F5-6MR | -1151.36 |
| T3-4MR | -1151.29 | E6-4MR | -1151.34 | D5-D6R | -1151.32 | F10-D6R | -1151.32 |
| T4-6MR | -1151.34 | E12-6MR | -1151.33 | D11-D6R | -1151.29 | F2-D6R | -1151.29 |
| T6-6MR | -1151.33 | E10-6MR | -1151.33 | D3-D6R | -1151.26 | F8-D6R | -1151.26 |
| T7-6MR | -1151.32 | E3-6MR | -1151.31 | D4-D6R | -1151.26 | F9-D6R | -1151.26 |
| T4-D6R | -1151.28 | E12-D6R | -1151.27 | D10-D6R | -1151.25 | F3-D6R | -1151.25 |
| T7-4MR | -1151.17 | E3-4MR | -1151.14 | D11-4MR | -1151.22 | F7-D6R | -1151.21 |
| T2-4MR | -1151.14 | E2-4MR | -1151.15 | D2-D6R | -1151.21 | F9-4MR | -1151.18 |
| T5-4MR | -1151.14 | E11-4MR | -1151.12 | D12-4MR | -1151.20 | F4-D6R | -1151.17 |
| T6-4MR | -1151.12 | E10-4MR | -1151.12 | D4-4MR | -1151.18 | F6-4MR | -1151.16 |
| T4-4MR | -1151.07 | E12-4MR | -1151.06 | D9-D6R | -1151.17 | F2-4MR | -1151.16 |
| T9-4MR | -1151.14 | E5-4MR | -1151.14 | D3-4MR | -1151.16 | F11-D6R | -1151.15 |
| T8-4MR | -1151.12 | E4-4MR | -1151.12 | D6-D6R | -1151.15 | F1-4MR | -1151.15 |
|  |  |  |  | D1-D6R | -1151.13 | F8-4MR | -1151.14 |
|  |  |  |  | D2-4MR | -1151.13 | F6-D6R | -1151.13 |
|  |  |  |  | D10-4MR | -1151.12 | F7-4MR | -1151.13 |
|  |  |  |  | D5-4MR | -1151.12 | F10-4MR | -1151.12 |
|  |  |  |  | D9-4MR | -1151.09 | F5-4MR | -1151.12 |
|  |  |  |  | D8-D6R | -1151.08 | F11-4MR | -1151.11 |
|  |  |  |  | D8-4MR | -1151.07 | F3-4MR | -1151.10 |
|  |  |  |  | D6-4MR | -1150.97 | F4-4MR | -1151.08 |
|  |  |  |  | D1-4MR | -1150.93 | F5-D6R | -1151.08 |
| **Table S9-1**. Structural energies of Al distribution and corresponding Na^+^ positions on Y-zeolites  (Si/Al = 15, Si = 45, Al = 3) | | | | | | | |
| Based on  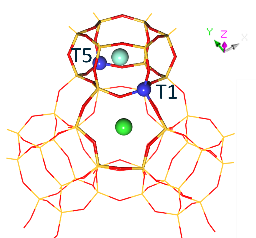  **T5-D6R** from Table S8  The first two sequences of Al:  T1-6MR→T5-D6R | | | | | | | |
| T zone | Energy (eV) | E zone | Energy (eV) | D zone | Energy (eV) | F zone | Energy (eV) |
| T2-6MR | -1151.32 | **E8-6MR** | **-1151.70** | D7-6MR | -1151.43 | F10-6MR | -1151.55 |
| T3-6MR | -1151.24 | E5-6MR | -1151.69 | D10-6MR | -1151.37 | F7-6MR | -1151.48 |
| T9-6MR | -1151.23 | E4-6MR | -1151.62 | D3-6MR | -1151.37 | F1-D6R | -1151.46 |
| T7-6MR | -1151.21 | E11-6MR | -1151.58 | D5-D6R | -1151.29 | F9-6MR | -1151.44 |
| T7-4MR | -1151.10 | E10-6MR | -1151.54 | D6-6MR | -1151.26 | F8-6MR | -1151.43 |
| T3-4MR | -1151.08 | E9-6MR | -1151.53 | D11-D6R | -1151.25 | F5-6MR | -1151.36 |
| T2-4MR | -1151.06 | E6-4MR | -1151.48 | D4-D6R | -1151.25 | F2-6MR | -1151.33 |
| T9-4MR | -1151.05 | E1-D6R | -1151.47 | D7-D6R | -1151.21 | F4-6MR | -1151.30 |
|  |  | E6-D6R | -1151.37 | D5-6MR | -1151.18 | F5-4MR | -1151.27 |
|  |  | E7-6MR | -1151.36 | D11-6MR | -1151.18 | F2-D6R | -1151.24 |
|  |  | E5-4MR | -1151.35 | D12-6MR | -1151.18 | F11-6MR | -1151.21 |
|  |  | E12-6MR | -1151.27 | D10-D6R | -1151.18 | F3-6MR | -1151.21 |
|  |  | E1-4MR | -1151.25 | D12-D6R | -1151.17 | F6-6MR | -1151.20 |
|  |  | E4-4MR | -1151.24 | D8-D6R | -1151.17 | F8-4MR | -1151.17 |
|  |  | E3-6MR | -1151.22 | D6-D6R | -1151.16 | F10-D6R | -1151.16 |
|  |  | E8-D6R | -1151.09 | D6-4MR | -1151.14 | F9-D6R | -1151.12 |
|  |  | E9-4MR | -1151.07 | D4-6MR | -1151.13 | F3-D6R | -1151.12 |
|  |  | E10-4MR | -1151.06 | D1-4MR | -1151.13 | F3-4MR | -1151.11 |
|  |  | E7-D6R | -1151.04 | D8-6MR | -1151.13 | F5-D6R | -1151.10 |
|  |  | E3-4MR | -1151.03 | D1-6MR | -1151.12 | F6-D6R | -1151.08 |
|  |  | E9-D6R | -1151.03 | D3-D6R | -1151.09 | F2-4MR | -1151.06 |
|  |  | E12-4MR | -1151.02 | D5-4MR | -1151.05 | F1-4MR | -1151.05 |
|  |  | E5-D6R | -1151.00 | D1-D6R | -1151.04 | F9-4MR | -1151.04 |
|  |  | E7-4MR | -1150.98 | D12-4MR | -1151.02 | F11-4MR | -1151.04 |
|  |  | E11-4MR | -1150.98 | D9-D6R | -1151.00 | F11-D6R | -1151.02 |
|  |  | E8-4MR | -1150.97 | D9-4MR | -1150.98 | F4-D6R | -1151.01 |
|  |  | E12-D6R | -1150.96 | D9-6MR | -1150.98 | F4-4MR | -1151.00 |
|  |  | E10-D6R | -1150.95 | D4-4MR | -1150.93 | F7-D6R | -1151.00 |
|  |  | E3-D6R | -1150.94 | D11-4MR | -1150.92 | F6-4MR | -1150.96 |
|  |  | E4-D6R | -1150.92 | D8-4MR | -1150.88 | F8-D6R | -1150.95 |
|  |  | E11-D6R | -1150.92 | D10-4MR | -1150.88 | F10-4MR | -1150.94 |
|  |  | E2-6MR | -1150.03 | D7-4MR | -1150.83 | F7-4MR | -1150.88 |
|  |  | E2-4MR | -1149.71 | D3-4MR | -1150.79 |  |  |
|  |  | E2-D6R | -1149.65 |  |  |  |  |
| **Table S10**. Structural energies of Al distribution and corresponding Na^+^ positions on Y-zeolites  (Si/Al = 11, Si = 44, Al = 4) | | | | | | | |
| Based on  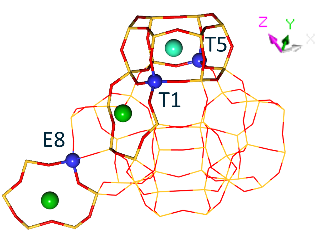  **T5-D6R** from Table S9  The first three sequences of Al:  T1-6MR→E8-6MR→T5-D6R | | | | | | | |
| T zone | Energy (eV) | E zone | Energy (eV) | D zone | Energy (eV) | F zone | Energy (eV) |
| T2-6MR | -1153.75 | **E4-6MR** | **-1154.00** | D12-D6R | -1153.82 | F1-D6R | -1153.72 |
| T9-6MR | -1153.63 | E11-D6R | -1153.94 | D3-6MR | -1153.80 | F11-6MR | -1153.71 |
| T3-4MR | -1153.52 | E5-6MR | -1153.92 | D5-6MR | -1153.71 | F10-6MR | -1153.69 |
| T7-6MR | -1153.50 | E10-D6R | -1153.84 | D11-6MR | -1153.68 | F4-6MR | -1153.69 |
| T7-4MR | -1153.49 | E6-D6R | -1153.77 | D5-D6R | -1153.67 | F7-6MR | -1153.68 |
| T9-4MR middle | -1153.42 | E11-6MR | -1153.76 | D6-6MR | -1153.62 | F5-6MR | -1153.65 |
| T2-4MR | -1153.41 | E12-D6R | -1153.76 | D4-6MR | -1153.60 | F2-6MR | -1153.65 |
| T9-4MR | -1153.36 | E10-6MR | -1153.68 | D11-D6R | -1153.60 | F6-6MR | -1153.65 |
| T7-4MR | -1153.35 | E2-6MR | -1153.63 | D10-6MR | -1153.56 | F8-6MR | -1153.62 |
| T3-4MR middle | -1153.32 | E12-6MR | -1153.59 | D4-D6R | -1153.55 | F3-6MR | -1153.59 |
| T2-4MR middle | -1153.23 | E2-D6R | -1153.58 | D8-6MR | -1153.55 | F9-6MR | -1153.58 |
|  |  | E6-4MR | -1153.58 | D3-D6R | -1153.50 | F10-D6R | -1153.55 |
|  |  | E4-D6R | -1153.57 | D10-D6R | -1153.50 | F2-D6R | -1153.54 |
|  |  | E3-6MR | -1153.55 | D9-6MR | -1153.48 | F3-D6R | -1153.48 |
|  |  | E5-D6R | -1153.55 | D1-6MR | -1153.47 | F1-4MR | -1153.47 |
|  |  | E3-D6R | -1153.51 | D6-D6R | -1153.46 | F9-D6R | -1153.45 |
|  |  | E5-4MR | -1153.46 | D5-4MR | -1153.39 | F5-D6R | -1153.44 |
|  |  | E4-4MR | -1153.40 | D12-4MR | -1153.37 | F7-D6R | -1153.43 |
|  |  | E10-4MR | -1153.37 | D4-4MR | -1153.37 | F4-D6R | -1153.43 |
|  |  | E3-4MR | -1153.37 | D11-4MR | -1153.36 | F8-4MR | -1153.43 |
|  |  | E2-4MR | -1153.37 | D1-D6R | -1153.34 | F2-4MR | -1153.41 |
|  |  | E11-4MR | -1153.37 | D1-4MR | -1153.33 | F9-4MR | -1153.40 |
|  |  | E12-4MR | -1153.26 | D6-4MR | -1153.32 | F3-4MR | -1153.40 |
|  |  | E2-4MR middle | -1153.26 | D10-4MR | -1153.32 | F6-D6R | -1153.39 |
|  |  |  |  | D8-D6R | -1153.31 | F11-4MR | -1153.38 |
|  |  |  |  | D9-4MR | -1153.31 | F11-D6R | -1153.38 |
|  |  |  |  | D8-4MR | -1153.27 | F4-4MR | -1153.37 |
|  |  |  |  | D9-D6R | -1153.24 | F5-4MR | -1153.35 |
|  |  |  |  | D3-4MR | -1153.24 | F10-4MR | -1153.35 |
|  |  |  |  |  |  | F6-4MR | -1153.33 |
|  |  |  |  |  |  | F8-D6R | -1153.27 |
|  |  |  |  |  |  | F7-4MR | -1153.27 |
| **Table S11**. Structural energies of Al distribution and corresponding Na^+^ positions on Y-zeolites  (Si/Al = 8.6, Si = 43, Al = 5) | | | | | | | |
| Based on  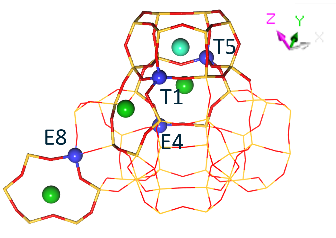  **E4-6MR** from Table S10  The first four sequences of Al:  T1-6MR→E8-6MR→T5-D6R→E4-6MR | | | | | | | |
| T zone | Energy (eV) | E zone | Energy (eV) | D zone | Energy (eV) | F zone | Energy (eV) |
| T9-6MR | -1156.08 | **E6-D6R** | **-1156.34** | D5-6MR | -1156.10 | F1-D6R | -1156.07 |
| T2-6MR | -1156.05 | E2-D6R | -1156.00 | D12-D6R | -1156.03 | F2-6MR | -1156.03 |
| T7-6MR | -1155.86 | E10-D6R | -1155.88 | D9-6MR | -1155.96 | F11-6MR | -1156.02 |
| T3-4MR | -1155.83 | E2-6MR | -1155.87 | D8-6MR | -1155.94 | F3-6MR | -1156.01 |
| T2-4MR | -1155.72 | E12-D6R | -1155.84 | D11-6MR | -1155.93 | F8-6MR | -1156.00 |
| T9-4MR | -1155.70 | E10-6MR | -1155.83 | D4-6MR | -1155.92 | F10-6MR | -1155.96 |
| T7-4MR middle | -1155.60 | E6-4MR | -1155.81 | D6-6MR | -1155.91 | F7-6MR | -1155.94 |
| T7-4MR | -1155.58 | E12-6MR | -1155.71 | D5-D6R | -1155.86 | F5-6MR | -1155.89 |
| T9-4MR | -1155.57 | E6-4MR middle | -1155.66 | D1-6MR | -1155.84 | F6-6MR | -1155.88 |
| T3-4MR middle | -1155.53 | E10-4MR | -1155.53 | D10-6MR | -1155.84 | F4-6MR | -1155.88 |
| T2-4MR middle | -1155.50 | E2-4MR | -1155.53 | D11-D6R | -1155.83 | F10-D6R | -1155.87 |
|  |  | E12-4MR middle | -1155.53 | D6-D6R | -1155.74 | F9-6MR | -1155.83 |
|  |  | E2-4MR middle | -1155.43 | D1-D6R | -1155.72 | F2-D6R | -1155.80 |
|  |  | E12-4MR | -1155.39 | D10-D6R | -1155.71 | F3-D6R | -1155.79 |
|  |  |  |  | D6-4MR | -1155.69 | F5-D6R | -1155.78 |
|  |  |  |  | D1-4MR | -1155.69 | F1-4MR | -1155.77 |
|  |  |  |  | D11-4MR | -1155.68 | F9-D6R | -1155.77 |
|  |  |  |  | D4-4MR | -1155.66 | F4-D6R | -1155.73 |
|  |  |  |  | D4-D6R | -1155.65 | F11-D6R | -1155.72 |
|  |  |  |  | D5-4MR | -1155.64 | F4-4MR | -1155.70 |
|  |  |  |  | D9-D6R | -1155.64 | F2-4MR | -1155.69 |
|  |  |  |  | D12-4MR | -1155.63 | F5-4MR | -1155.64 |
|  |  |  |  | D10-4MR | -1155.62 | F3-4MR | -1155.64 |
|  |  |  |  | D8-D6R | -1155.62 | F6-D6R | -1155.63 |
|  |  |  |  | D8-4MR | -1155.59 | F10-4MR | -1155.62 |
|  |  |  |  | D9-4MR | -1155.55 | F8-D6R | -1155.61 |
|  |  |  |  |  |  | F7-4MR | -1155.58 |
|  |  |  |  |  |  | F9-4MR | -1155.58 |
|  |  |  |  |  |  | F6-4MR | -1155.56 |
|  |  |  |  |  |  | F11-4MR | -1155.55 |
|  |  |  |  |  |  | F8-4MR | -1155.52 |
|  |  |  |  |  |  | F7-D6R | -1155.50 |
| **Table S11-1**. Structural energies of Al distribution and corresponding Na^+^ positions on Y-zeolites  (Si/Al = 8.6, Si = 43, Al = 5) | | | | | | | |
| Based on  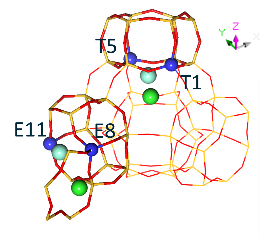  **E11-D6R** from Table S10  The first four sequences of Al:  T1-6MR→E8-6MR→T5-D6R→E11-D6R | | | | | | | |
| T zone | Energy (eV) | E zone | Energy (eV) | D zone | Energy (eV) | F zone | Energy (eV) |
| **T9-6MR** | **-1156.18** | E5-6MR | -1156.16 | D12-D6R | -1156.12 | F1-D6R | -1156.13 |
| T3-D6R | -1156.12 | E6-D6R | -1156.11 | D3-6MR | -1156.01 | F8-6MR | -1156.01 |
| T2-6MR | -1156.02 | E2-6MR | -1156.01 | D10-6MR | -1155.96 | F10-6MR | -1155.95 |
| T2-D6R | -1155.97 | E2-D6R | -1155.96 | D5-6MR | -1155.95 | F2-6MR | -1155.94 |
| T3-4MR | -1155.81 | E6-4MR | -1155.80 | D11-6MR | -1155.94 | F10-D6R | -1155.91 |
| T7-6MR | -1155.76 | E3-6MR | -1155.75 | D10-D6R | -1155.91 | F11-6MR | -1155.90 |
| T9-D6R | -1155.75 | E5-D6R | -1155.74 | D5-D6R | -1155.90 | F11-D6R | -1155.89 |
| T9-4MR middle | -1155.72 | E3-4MR middle | -1155.71 | D6-6MR | -1155.90 | F2-D6R | -1155.86 |
| T7-T7T5 middle | -1155.71 | E5-4MR middle | -1155.71 | D11-D6R | -1155.86 | F5-6MR | -1155.85 |
| T7-D6R | -1155.69 | E3-D6R-2Na | -1155.68 | D8-6MR | -1155.85 | F9-6MR | -1155.80 |
| T3-4MR middle | -1155.67 | E6-4MR middle | -1155.67 | D4-6MR | -1155.81 | F3-6MR | -1155.80 |
| T9-4MR | -1155.67 | E5-4MR | -1155.66 | D4-D6R | -1155.77 | F9-D6R | -1155.77 |
| T2-4MR | -1155.66 | E2-4MR | -1155.66 | D9-6MR | -1155.73 | F3-D6R | -1155.75 |
| T7-4MR | -1155.60 | E3-4MR | -1155.59 | D1-6MR | -1155.72 | F6-6MR | -1155.73 |
| T2-4MR middle | -1155.45 | E2-4MR middle | -1155.45 | D8-D6R | -1155.71 | F4-6MR | -1155.72 |
|  |  |  |  | D12-4MR | -1155.66 | F5-D6R | -1155.71 |
|  |  |  |  | D5-4MR | -1155.63 | F8-D6R | -1155.70 |
|  |  |  |  | D10-4MR | -1155.63 | F1-4MR | -1155.68 |
|  |  |  |  | D1-D6R | -1155.62 | F10-4MR | -1155.63 |
|  |  |  |  | D11-4MR | -1155.62 | F2-4MR | -1155.63 |
|  |  |  |  | D9-4MR | -1155.61 | F11-4MR | -1155.63 |
|  |  |  |  | D4-4MR | -1155.59 | F3-4MR | -1155.63 |
|  |  |  |  | D8-4MR | -1155.57 | F6-D6R | -1155.62 |
|  |  |  |  | D3-D6R | -1155.56 | F4-4MR | -1155.62 |
|  |  |  |  | D3-4MR | -1155.56 | F9-4MR | -1155.60 |
|  |  |  |  | D9-D6R | -1155.53 | F5-4MR | -1155.56 |
|  |  |  |  | D1-4MR | -1155.51 | F4-D6R | -1155.53 |
|  |  |  |  |  |  | F8-4MR | -1155.53 |
|  |  |  |  |  |  | F6-4MR | -1155.52 |
| **Table S12**. Structural energies of Al distribution and corresponding Na^+^ positions on Y-zeolites  (Si/Al = 7, Si = 42, Al = 6) | | | | | | | |
| Based on  **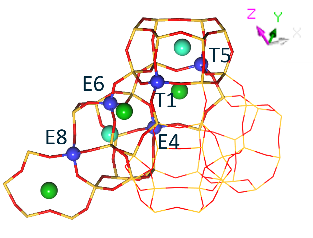**  **E6-D6R** from Table S11  The first five sequences of Al:  T1-6MR→E8-6MR→T5-D6R→E4-6MR→E6-D6R | | | | | | | |
| T zone | Energy (eV) | E zone | Energy (eV) | D zone | Energy (eV) | F zone | Energy (eV) |
| **T9-6MR** | **-1158.47** | E10-6MR | -1158.44 | **D12-D6R** | **-1158.46** | F11-6MR | -1158.38 |
| T2-6MR | -1158.28 | E2-6MR | -1158.32 | D11-6MR | -1158.34 | F6-6MR | -1158.32 |
| T7-6MR | -1158.21 | E12-6MR | -1158.24 | D5-6MR | -1158.34 | F1-D6R | -1158.31 |
| T3-4MR | -1158.20 | E10-4MR middle | -1158.17 | D5-D6R | -1158.32 | F10-6MR | -1158.30 |
| T2-4MR | -1158.11 | E10-4MR | -1158.16 | D6-6MR | -1158.30 | F2-6MR | -1158.29 |
| T9-4MR | -1158.09 | E2-4MR middle | -1158.09 | D11-D6R | -1158.29 | F5-6MR | -1158.28 |
| T9-4MR middle | -1158.08 | E2-4MR | -1158.03 | D9-6MR | -1158.28 | F4-6MR | -1158.28 |
| T3-4MR middle | -1158.01 | E12-4MR middle | -1157.98 | D4-D6R | -1158.28 | F2-D6R | -1158.26 |
| T7-4MR middle | -1157.95 | E12-4MR | -1157.92 | D4-6MR | -1158.27 | F7-6MR | -1158.24 |
| T7-4MR | -1157.95 |  |  | D8-6MR | -1158.25 | F9-6MR | -1158.23 |
| T2-4MR middle | -1157.87 |  |  | D1-6MR | -1158.24 | F8-6MR | -1158.23 |
|  |  |  |  | D1-D6R | -1158.24 | F3-6MR | -1158.22 |
|  |  |  |  | D6-D6R | -1158.24 | F5-D6R | -1158.19 |
|  |  |  |  | D10-6MR | -1158.19 | F10-D6R | -1158.18 |
|  |  |  |  | D8-4MR | -1158.16 | F4-D6R | -1158.17 |
|  |  |  |  | D10-D6R | -1158.14 | F3-D6R | -1158.16 |
|  |  |  |  | D1-4MR | -1158.12 | F9-D6R | -1158.15 |
|  |  |  |  | D4-4MR middle | -1158.09 | F1-4MR | -1158.13 |
|  |  |  |  | D4-4MR | -1158.08 | F6-D6R | -1158.06 |
|  |  |  |  | D11-4MR | -1158.07 | F11-D6R | -1158.05 |
|  |  |  |  | D6-4MR | -1158.07 | F4-4MR | -1158.05 |
|  |  |  |  | D5-4MR | -1158.03 | F2-4MR | -1158.04 |
|  |  |  |  | D1-4MR middle | -1158.03 | F5-4MR | -1158.02 |
|  |  |  |  | D9-D6R | -1158.03 | F7-D6R | -1158.00 |
|  |  |  |  | D12-4MR | -1158.01 | F7-4MR | -1157.99 |
|  |  |  |  | D8-D6R | -1157.98 | F8-D6R | -1157.98 |
|  |  |  |  | D10-4MR | -1157.93 | F3-4MR | -1157.98 |
|  |  |  |  | D9-4MR | -1157.85 | F6-4MR | -1157.98 |
|  |  |  |  |  |  | F10-4MR | -1157.94 |
|  |  |  |  |  |  | F5-4MR middle | -1157.94 |
|  |  |  |  |  |  | F9-4MR | -1157.93 |
|  |  |  |  |  |  | F11-4MR | -1157.92 |
|  |  |  |  |  |  | F8-4MR | -1157.89 |
| **Table S12-1**. Structural energies of Al distribution and corresponding Na^+^ positions on Y-zeolites  (Si/Al = 7, Si = 42, Al = 6) | | | | | | | |
| Based on  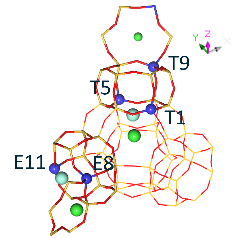  **T9-6MR** from Table S11-1  The first five sequences of Al:  T1-6MR→E8-6MR→T5-D6R→E11-D6R→T9-6MR | | | | | | | |
| T zone | Energy (eV) | E zone | Energy (eV) | D zone | Energy (eV) | F zone | Energy (eV) |
| T2-6MR | -1158.22 | **E5-6MR** | **-1158.52** | D12-D6R | -1158.33 | F4-6MR | -1158.34 |
| T7-6MR | -1157.95 | E6-D6R | -1158.38 | D3-6MR | -1158.29 | F5-6MR | -1158.28 |
| T7-4MR middle | -1157.93 | E2-6MR | -1158.22 | D6-6MR | -1158.22 | F2-6MR | -1158.21 |
| T2-4MR | -1157.89 | E2-D6R | -1158.20 | D11-6MR | -1158.18 | F1-D6R | -1158.21 |
| T7-4MR | -1157.80 | E6-4MR | -1158.08 | D5-D6R | -1158.16 | F10-6MR | -1158.20 |
| T2-4MR middle | -1157.72 | E5-D6R | -1158.02 | D11-D6R | -1158.15 | F9-6MR | -1158.15 |
|  |  | E6-4MR middle | -1157.95 | D5-6MR | -1158.12 | F3-6MR | -1158.13 |
|  |  | E5-4MR | -1157.92 | D4-D6R | -1158.04 | F6-6MR | -1158.13 |
|  |  | E3-6MR | -1157.92 | D10-D6R | -1158.03 | F11-6MR | -1158.13 |
|  |  | E2-4MR | -1157.92 | D4-6MR | -1158.01 | F8-4MR | -1157.99 |
|  |  | E3-D6R | -1157.87 | D10-6MR | -1158.00 | F2-D6R | -1157.98 |
|  |  | E3-4MR | -1157.86 | D6-D6R | -1158.00 | F10-D6R | -1157.97 |
|  |  | E5-4MR middle | -1157.84 | D3-D6R | -1157.98 | F9-4MR | -1157.93 |
|  |  | E3-4MR middle | -1157.79 | D8-6MR | -1157.98 | F11-4MR | -1157.92 |
|  |  | E2-4MR middle | -1157.67 | D8-D6R | -1157.96 | F9-D6R | -1157.92 |
|  |  |  |  | D1-6MR | -1157.93 | F2-4MR | -1157.90 |
|  |  |  |  | D12-4MR | -1157.90 | F6-4MR | -1157.90 |
|  |  |  |  | D9-6MR | -1157.89 | F3-D6R | -1157.89 |
|  |  |  |  | D11-4MR | -1157.88 | F5-4MR | -1157.89 |
|  |  |  |  | D8-4MR | -1157.86 | F1-4MR | -1157.88 |
|  |  |  |  | D9-4MR | -1157.84 | F10-4MR | -1157.87 |
|  |  |  |  | D4-4MR | -1157.82 | F8-D6R | -1157.86 |
|  |  |  |  | D1-4MR middle | -1157.82 | F6-D6R | -1157.85 |
|  |  |  |  | D10-4MR | -1157.82 | F3-4MR | -1157.84 |
|  |  |  |  | D9-D6R | -1157.81 | F4-4MR | -1157.82 |
|  |  |  |  | D1-4MR | -1157.80 | F5-D6R | -1157.81 |
|  |  |  |  | D5-4MR | -1157.75 | F4-D6R | -1157.80 |
|  |  |  |  | D6-4MR | -1157.67 | F11-D6R | -1157.78 |
|  |  |  |  | D3-4MR | -1157.65 |  |  |
|  |  |  |  | D1-D6R | -1157.65 |  |  |
| **Table S13.** Structural energies of Al distribution and corresponding Na^+^ positions on Y-zeolites  (Si/Al = 5.86, Si = 41, Al = 7) | | | | | | | |
| Based on  **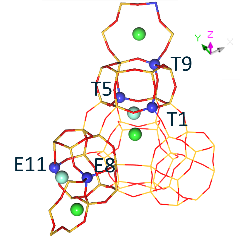**  **T9-6MR** from Table S12  The first six sequences of Al:  T1-6MR→E8-6MR→T5-D6R→E4-6MR→E6-D6R→T9-6MR | | | | | | | |
| T zone | Energy (eV) | E zone | Energy (eV) | D zone | Energy (eV) | F zone | Energy (eV) |
| T2-6MR | -1160.34 | E12-6MR | -1160.40 | D12-D6R | -1160.52 | **F4-6MR** | **-1160.69** |
| T7-6MR | -1160.25 | E2-6MR | -1160.38 | D6-6MR | -1160.44 | F6-6MR | -1160.53 |
| T2-4MR | -1160.18 | E2-4MR middle | -1160.17 | D11-D6R | -1160.44 | F5-6MR | -1160.53 |
| T7-4MR middle | -1160.01 | E2-4MR | -1160.14 | D11-6MR | -1160.42 | F7-D6R | -1160.49 |
| T2-4MR middle | -1160.01 | E12-4MR | -1160.05 | D5-D6R | -1160.41 | F11-6MR | -1160.47 |
| T7-4MR | -1159.98 | E12-4MR middle | -1160.03 | D4-D6R | -1160.41 | F9-6MR | -1160.43 |
|  |  |  |  | D6-D6R | -1160.35 | F2-6MR | -1160.40 |
|  |  |  |  | D5-6MR | -1160.35 | F10-6MR | -1160.39 |
|  |  |  |  | D4-6MR | -1160.31 | F3-6MR | -1160.38 |
|  |  |  |  | D9-6MR | -1160.30 | F7-4MR | -1160.34 |
|  |  |  |  | D1-6MR | -1160.28 | F4-D6R | -1160.26 |
|  |  |  |  | D10-D6R | -1160.27 | F1-D6R | -1160.23 |
|  |  |  |  | D8-6MR | -1160.26 | F2-D6R | -1160.21 |
|  |  |  |  | D10-6MR | -1160.23 | F8-4MR | -1160.21 |
|  |  |  |  | D8-4MR | -1160.23 | F1-4MR | -1160.21 |
|  |  |  |  | D1-4MR middle | -1160.20 | F8-D6R | -1160.20 |
|  |  |  |  | D1-4MR | -1160.19 | F6-4MR | -1160.17 |
|  |  |  |  | D9-D6R | -1160.18 | F5-4MR | -1160.16 |
|  |  |  |  | D11-4MR | -1160.16 | F9-D6R | -1160.16 |
|  |  |  |  | D4-4MR | -1160.13 | F6-D6R | -1160.15 |
|  |  |  |  | D1-D6R | -1160.12 | F3-D6R | -1160.15 |
|  |  |  |  | D12-4MR | -1160.10 | F2-4MR | -1160.14 |
|  |  |  |  | D4-4MR middle | -1160.09 | F5-D6R | -1160.14 |
|  |  |  |  | D8-D6R | -1160.09 | F9-4MR | -1160.11 |
|  |  |  |  | D5-4MR | -1160.00 | F4-4MR | -1160.10 |
|  |  |  |  | D6-4MR | -1159.98 | F10-D6R | -1160.10 |
|  |  |  |  | D10-4MR | -1159.97 | F3-4MR | -1160.06 |
|  |  |  |  | D9-4MR | -1159.93 | F11-4MR | -1160.04 |
|  |  |  |  |  |  | F10-4MR | -1160.03 |
|  |  |  |  |  |  | F11-D6R | -1160.00 |
| **Table S13-1**. Structural energies of Al distribution and corresponding Na^+^ positions on Y-zeolites  (Si/Al = 5.86, Si = 41, Al = 7) | | | | | | | |
| Based on  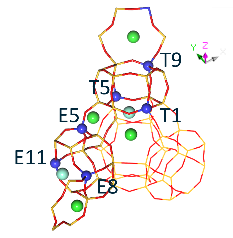  **E5-6MR** from Table S12-1  The first six sequences of Al:  T1-6MR→E8-6MR→T5-D6R→E11-D6R→T9-6MR→E5-6MR | | | | | | | |
| T zone | Energy (eV) | E zone | Energy (eV) | D zone | Energy (eV) | F zone | Energy (eV) |
| T2-6MR | -1160.50 | E2-D6R | -1160.85 | **D9-6MR** | **-1160.58** | F4-6MR | -1160.59 |
| T2-4MR | -1160.23 | E3-4MR middle | -1160.51 | D6-6MR | -1160.53 | F11-6MR | -1160.54 |
| T7-6MR | -1160.20 | E2-6MR | -1160.50 | D11-6MR | -1160.53 | F5-6MR | -1160.52 |
| T7-4MR | -1160.15 | E2-4MR | -1160.24 | D8-6MR | -1160.52 | F2-6MR | -1160.52 |
| T7-4MR middle | -1160.09 | E3-6MR | -1160.20 | D12-D6R | -1160.50 | F1-D6R | -1160.51 |
| T2-4MR middle | -1160.04 | E3-4MR | -1160.15 | D5-6MR | -1160.47 | F10-6MR | -1160.46 |
|  |  | E2-4MR middle | -1160.04 | D4-6MR | -1160.46 | F9-6MR | -1160.45 |
|  |  |  |  | D10-6MR | -1160.45 | F3-6MR | -1160.45 |
|  |  |  |  | D1-6MR | -1160.42 | F6-6MR | -1160.42 |
|  |  |  |  | D11-D6R | -1160.36 | F2-D6R | -1160.35 |
|  |  |  |  | D5-D6R | -1160.32 | F10-D6R | -1160.31 |
|  |  |  |  | D10-D6R | -1160.28 | F9-D6R | -1160.28 |
|  |  |  |  | D4-D6R | -1160.28 | F3-D6R | -1160.27 |
|  |  |  |  | D1-4MR middle | -1160.26 | F5-4MR | -1160.26 |
|  |  |  |  | D8-4MR | -1160.26 | F8-D6R | -1160.26 |
|  |  |  |  | D3-D6R | -1160.25 | F6-4MR | -1160.23 |
|  |  |  |  | D1-4MR | -1160.24 | F9-4MR | -1160.23 |
|  |  |  |  | D11-4MR | -1160.24 | F2-4MR | -1160.23 |
|  |  |  |  | D4-4MR | -1160.23 | F1-4MR | -1160.21 |
|  |  |  |  | D12-4MR | -1160.21 | F8-4MR | -1160.20 |
|  |  |  |  | D3-4MR | -1160.20 | F4-D6R | -1160.18 |
|  |  |  |  | D9-D6R | -1160.19 | F5-D6R | -1160.17 |
|  |  |  |  | D6-D6R | -1160.16 | F11-D6R | -1160.17 |
|  |  |  |  | D8-D6R | -1160.16 | F4-4MR | -1160.14 |
|  |  |  |  | D9-4MR | -1160.14 | F3-4MR | -1160.12 |
|  |  |  |  | D5-4MR | -1160.07 | F10-4MR | -1160.07 |
|  |  |  |  | D6-4MR | -1160.03 | F11-4MR | -1160.04 |
|  |  |  |  | D1-D6R | -1159.98 | F6-D6R | -1159.98 |
| **Table S14**. Structural energies of Al distribution and corresponding Na^+^ positions on Y-zeolites  (Si/Al = 5, Si = 40, Al = 8) | | | | | | | |
| Based on  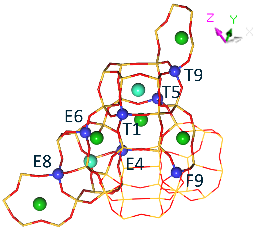  **F4-6MR** from Table S13  The first seven sequences of Al:  T1-6MR→E8-6MR→T5-D6R→E4-6MR→E6-D6R→T9-6MR→F4-6MR | | | | | | | |
| T zone | Energy (eV) | E zone | Energy (eV) | D zone | Energy (eV) | F zone | Energy (eV) |
| T2-6MR | -1162.62 | E2-6MR | -1162.71 | D1-D6R | -1162.74 | **F1-D6R** | **-1162.79** |
| T7-6MR | -1162.53 | E12-6MR | -1162.62 | D12-D6R | -1162.69 | F11-6MR | -1162.67 |
| T2-4MR | -1162.42 | E2-4MR middle | -1162.40 | D11-6MR | -1162.69 | F6-6MR | -1162.66 |
| T7-4MR middle | -1162.26 | E2-4MR | -1162.40 | D4-6MR | -1162.68 | F10-D6R | -1162.64 |
| T2-4MR middle | -1162.24 | E12-4MR middle | -1162.28 | D5-6MR | -1162.65 | F10-6MR | -1162.64 |
| T7-4MR | -1162.24 | E12-4MR | -1162.26 | D8-6MR | -1162.65 | F2-D6R | -1162.61 |
|  |  |  |  | D9-6MR | -1162.64 | F9-D6R | -1162.60 |
|  |  |  |  | D11-D6R | -1162.62 | F6-D6R | -1162.59 |
|  |  |  |  | D4-D6R | -1162.57 | F2-6MR | -1162.58 |
|  |  |  |  | D10-6MR | -1162.57 | F9-6MR | -1162.56 |
|  |  |  |  | D10-D6R | -1162.51 | F8-D6R | -1162.54 |
|  |  |  |  | D5-D6R | -1162.49 | F11-D6R | -1162.53 |
|  |  |  |  | D8-4MR | -1162.48 | F1-4MR | -1162.44 |
|  |  |  |  | D1-4MR | -1162.46 | F8-4MR | -1162.34 |
|  |  |  |  | D9-D6R | -1162.42 | F6-4MR | -1162.28 |
|  |  |  |  | D8-D6R | -1162.42 | F9-4MR | -1162.26 |
|  |  |  |  | D11-4MR | -1162.38 | F2-4MR | -1162.26 |
|  |  |  |  | D4-4MR | -1162.36 | F11-4MR | -1162.24 |
|  |  |  |  | D12-4MR | -1162.34 | F10-4MR | -1162.24 |
|  |  |  |  | D1-4MR middle | -1162.33 |  |  |
|  |  |  |  | D4-4MR middle | -1162.31 |  |  |
|  |  |  |  | D5-4MR | -1162.28 |  |  |
|  |  |  |  | D10-4MR | -1162.24 |  |  |
|  |  |  |  | D9-4MR | -1162.20 |  |  |
| **Table S14-1**. Structural energies of Al distribution and corresponding Na^+^ positions on Y-zeolites  (Si/Al = 5, Si = 40, Al = 8) | | | | | | | |
| Based on  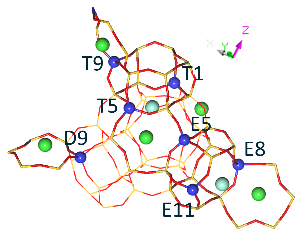  **D9-6MR** from Table S13-1  The first seven sequences of Al:  T1-6MR→E8-6MR→T5-D6R→E11-D6R→T9-6MR→E5-6MR→D9-6MR | | | | | | | |
| T zone | Energy (eV) | E zone | Energy (eV) | D zone | Energy (eV) | F zone | Energy (eV) |
| T2-6MR | -1162.65 | E2-D6R | -1162.91 | **D12-D6R** | **-1162.87** | F5-6MR | -1162.75 |
| T7-6MR | -1162.39 | E2-6MR | -1162.61 | D5-D6R | -1162.66 | F4-6MR | -1162.75 |
| T2-4MR | -1162.29 | E3-D6R | -1162.54 | D6-6MR | -1162.56 | F9-6MR | -1162.62 |
| T7-4MR | -1162.17 | E3-6MR | -1162.29 | D11-D6R | -1162.56 | F2-6MR | -1162.61 |
| T7-4MR middle | -1162.16 | E2-4MR | -1162.27 | D5-6MR | -1162.53 | F3-6MR | -1162.59 |
| T2-4MR middle | -1162.10 | E3-4MR | -1162.22 | D11-6MR | -1162.53 | F10-6MR | -1162.58 |
|  |  | E3-4MR middle | -1162.17 | D4-D6R | -1162.50 | F1-D6R | -1162.49 |
|  |  | E2-4MR middle | -1162.09 | D6-D6R | -1162.48 | F6-D6R | -1162.44 |
|  |  |  |  | D4-6MR | -1162.40 | F2-D6R | -1162.37 |
|  |  |  |  | D3-D6R | -1162.37 | F6-4MR | -1162.34 |
|  |  |  |  | D1-6MR | -1162.35 | F3-D6R | -1162.33 |
|  |  |  |  | D12-4MR | -1162.25 | F5-D6R | -1162.32 |
|  |  |  |  | D1-D6R | -1162.20 | F8-4MR | -1162.32 |
|  |  |  |  | D4-4MR | -1162.20 | F8-D6R | -1162.31 |
|  |  |  |  | D11-4MR | -1162.15 | F1-4MR | -1162.30 |
|  |  |  |  | D1-4MR | -1162.14 | F2-4MR | -1162.27 |
|  |  |  |  | D3-4MR | -1162.13 | F9-4MR | -1162.27 |
|  |  |  |  | D5-4MR | -1162.09 | F4-D6R | -1162.26 |
|  |  |  |  | D1-4MR middle | -1162.08 | F9-D6R | -1162.25 |
|  |  |  |  | D6-4MR | -1162.04 | F5-4MR | -1162.25 |
|  |  |  |  |  |  | F4-4MR | -1162.24 |
|  |  |  |  |  |  | F10-D6R | -1162.21 |
|  |  |  |  |  |  | F3-4MR | -1162.21 |
|  |  |  |  |  |  | F10-4MR | -1162.17 |
| **Table S15**. Structural energies of Al distribution and corresponding Na^+^ positions on Y-zeolites  (Si/Al = 4.3, Si = 39, Al = 9) | | | | | | | |
| Based on  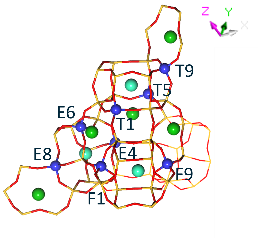  **F1-D6R** from Table S14  The first eight sequences of Al:  T1-6MR→E8-6MR→T5-D6R→E4-6MR→E6-D6R→T9-6MR→F4-6MR→F1-D6R | | | | | | | |
| T zone | Energy (eV) | E zone | Energy (eV) | D zone | Energy (eV) | F zone | Energy (eV) |
| T2-6MR | -1164.78 | E12-6MR | -1164.73 | D1-D6R | -1164.93 | **F6-6MR** | **-1165.05** |
| T7-6MR | -1164.73 | E2-6MR | -1164.71 | D12-D6R | -1164.87 | F9-6MR | -1164.87 |
| T2-4MR | -1164.56 | E2-4MR middle | -1164.67 | D5-6MR | -1164.85 | F11-6MR | -1164.81 |
| T2-4MR middle | -1164.42 | E2-4MR | -1164.47 | D5-D6R | -1164.82 | F8-4MR | -1164.68 |
| T7-4MR middle | -1164.41 | E12-4MR middle | -1164.40 | D4-6MR | -1164.80 | F6-4MR | -1164.65 |
| T7-4MR | -1164.40 | E12-4MR | -1164.36 | D11-6MR | -1164.78 | F9-4MR | -1164.55 |
|  |  |  |  | D4-D6R | -1164.74 | F11-4MR | -1164.51 |
|  |  |  |  | D11-D6R | -1164.73 |  |  |
|  |  |  |  | D9-6MR | -1164.68 |  |  |
|  |  |  |  | D8-6MR | -1164.68 |  |  |
|  |  |  |  | D10-6MR | -1164.65 |  |  |
|  |  |  |  | D8-4MR | -1164.63 |  |  |
|  |  |  |  | D9-D6R | -1164.61 |  |  |
|  |  |  |  | D1-4MR | -1164.61 |  |  |
|  |  |  |  | D8-D6R | -1164.55 |  |  |
|  |  |  |  | D4-4MR | -1164.55 |  |  |
|  |  |  |  | D10-D6R | -1164.54 |  |  |
|  |  |  |  | D10-4MR | -1164.51 |  |  |
|  |  |  |  | D4-4MR middle | -1164.49 |  |  |
|  |  |  |  | D1-4MR middle | -1164.49 |  |  |
|  |  |  |  | D5-4MR | -1164.45 |  |  |
|  |  |  |  | D11-4MR | -1164.44 |  |  |
|  |  |  |  | D12-4MR | -1164.42 |  |  |
|  |  |  |  | D9-4MR | -1164.33 |  |  |
| **Table S15-1**. Structural energies of Al distribution and corresponding Na^+^ positions on Y-zeolites  (Si/Al = 4.3, Si = 39, Al = 9) | | | | | | | |
| Based on  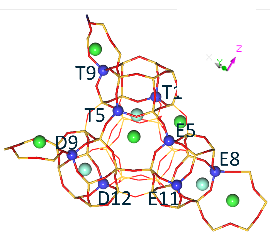  **D12-D6R** from Table S14-1  The first eight sequences of Al:  T1-6MR→E8-6MR→T5-D6R→E11-D6R→T9-6MR→E5-6MR→D9-6MR→D12-D6R | | | | | | | |
| T zone | Energy (eV) | E zone | Energy (eV) | D zone | Energy (eV) | F zone | Energy (eV) |
| T2-6MR | -1164.80 | E2-D6R | -1165.12 | D4-6MR | -1164.87 | **F5-6MR** | **-1164.97** |
| T7-6MR | -1164.62 | E2-6MR | -1164.94 | D1-6MR | -1164.86 | F4-6MR | -1164.96 |
| T2-4MR middle | -1164.53 | E3-D6R | -1164.83 | D6-6MR | -1164.86 | F10-6MR | -1164.96 |
| T2-4MR | -1164.52 | E3-6MR | -1164.67 | D1-4MR | -1164.74 | F9-6MR | -1164.89 |
| T7-4MR | -1164.45 | E2-4MR | -1164.60 | D4-4MR | -1164.66 | F2-6MR | -1164.88 |
| T7-4MR middle | -1164.44 | E3-4MR | -1164.54 | D3-4MR | -1164.65 | F1-D6R | -1164.84 |
|  |  | E3-4MR middle | -1164.48 | D1-4MR middle | -1164.54 | F3-6MR | -1164.82 |
|  |  | E2-4MR middle | -1164.46 | D6-4MR | -1164.49 | F6-D6R | -1164.80 |
|  |  |  |  |  |  | F10-D6R | -1164.70 |
|  |  |  |  |  |  | F8-D6R | -1164.66 |
|  |  |  |  |  |  | F2-D6R | -1164.65 |
|  |  |  |  |  |  | F8-4MR | -1164.65 |
|  |  |  |  |  |  | F6-4MR | -1164.65 |
|  |  |  |  |  |  | F9-4MR | -1164.62 |
|  |  |  |  |  |  | F5-D6R | -1164.62 |
|  |  |  |  |  |  | F3-4MR | -1164.61 |
|  |  |  |  |  |  | F4-D6R | -1164.60 |
|  |  |  |  |  |  | F9-D6R | -1164.58 |
|  |  |  |  |  |  | F1-4MR | -1164.55 |
|  |  |  |  |  |  | F4-4MR | -1164.54 |
|  |  |  |  |  |  | F5-4MR | -1164.54 |
|  |  |  |  |  |  | F3-D6R | -1164.53 |
|  |  |  |  |  |  | F2-4MR | -1164.51 |
|  |  |  |  |  |  | F10-4MR | -1164.51 |
| **Table S16**. Structural energies of Al distribution and corresponding Na^+^ positions on Y-zeolites  (Si/Al = 3.8, Si = 38, Al = 10) | | | | | | | |
| Based on  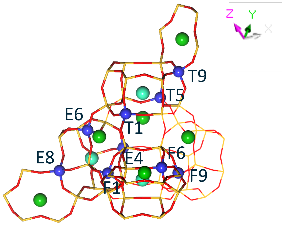  **F6-6MR** from Table S15  The first nine sequences of Al:  T1-6MR→E8-6MR→T5-D6R→E4-6MR→E6-D6R→T9-6MR→F4-6MR→F1-D6R→F6-6MR | | | | | | | |
| T zone | Energy (eV) | E zone | Energy (eV) | D zone | Energy (eV) | F zone | Energy (eV) |
| T2-6MR | -1167.01 | E2-6MR | -1167.03 | **D8-D6R** | **-1167.20** | F9-6MR | -1167.06 |
| T7-6MR | -1166.97 | E2-4MR middle | -1166.95 | D1-D6R | -1167.17 | F8-4MR | -1166.79 |
| T2-4MR | -1166.77 | E2-4MR | -1166.76 | D5-6MR | -1167.14 | F9-4MR | -1166.77 |
| T7-4MR middle | -1166.69 |  |  | D8-4MR | -1167.10 |  |  |
| T2-4MR middle | -1166.66 |  |  | D4-6MR | -1167.09 |  |  |
| T7-4MR | -1166.65 |  |  | D9-D6R | -1167.05 |  |  |
|  |  |  |  | D11-6MR | -1167.05 |  |  |
|  |  |  |  | D12-D6R | -1167.01 |  |  |
|  |  |  |  | D10-6MR | -1167.00 |  |  |
|  |  |  |  | D5-D6R | -1166.89 |  |  |
|  |  |  |  | D10-4MR | -1166.85 |  |  |
|  |  |  |  | D4-D6R | -1166.84 |  |  |
|  |  |  |  | D11-D6R | -1166.83 |  |  |
|  |  |  |  | D1-4MR middle | -1166.82 |  |  |
|  |  |  |  | D1-4MR | -1166.81 |  |  |
|  |  |  |  | D9-4MR | -1166.80 |  |  |
|  |  |  |  | D4-4MR | -1166.77 |  |  |
|  |  |  |  | D10-D6R | -1166.73 |  |  |
|  |  |  |  | D5-4MR | -1166.72 |  |  |
|  |  |  |  | D4-4MR middle | -1166.71 |  |  |
|  |  |  |  | D12-4MR | -1166.71 |  |  |
|  |  |  |  | D11-4MR | -1166.67 |  |  |
| **Table S16-1**. Structural energies of Al distribution and corresponding Na^+^ positions on Y-zeolites  (Si/Al = 3.8, Si = 38, Al = 10) | | | | | | | |
| Based on  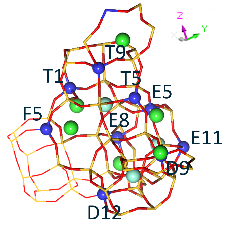  **T5-6MR** from Table S15-1  The first nine sequences of Al:  T1-6MR→E8-6MR→T5-D6R→E11-D6R→T9-6MR→E5-6MR→D9-6MR→D12-D6R→F5-6MR | | | | | | | |
| T zone | Energy (eV) | E zone | Energy (eV) | D zone | Energy (eV) | F zone | Energy (eV) |
| T2-6MR | -1166.95 | E2-6MR | -1167.25 | D1-4MR | -1167.07 | **F2-6MR** | **-1167.16** |
| T7-6MR | -1166.78 | E2-D6R | -1167.23 | D4-6MR | -1166.98 | F1-D6R | -1167.09 |
| T2-4MR middle | -1166.62 | E3-D6R | -1166.95 | D1-4MR middle | -1166.96 | F2-D6R | -1167.06 |
| T2-4MR | -1166.61 | E3-6MR | -1166.75 | D6-4MR | -1166.80 | F8-D6R | -1167.04 |
| T7-4MR | -1166.58 | E2-4MR | -1166.66 | D3-4MR | -1166.77 | F9-D6R | -1166.99 |
| T7-4MR middle | -1166.54 | E3-4MR | -1166.59 | D4-4MR | -1166.75 | F10-D6R | -1166.98 |
|  |  | E3-4MR middle | -1166.58 |  |  | F9-6MR | -1166.97 |
|  |  | E2-4MR middle | -1166.57 |  |  | F3-D6R | -1166.93 |
|  |  |  |  |  |  | F10-6MR | -1166.91 |
|  |  |  |  |  |  | F3-6MR | -1166.85 |
|  |  |  |  |  |  | F8-4MR | -1166.68 |
|  |  |  |  |  |  | F9-4MR | -1166.67 |
|  |  |  |  |  |  | F3-4MR | -1166.65 |
|  |  |  |  |  |  | F2-4MR | -1166.58 |
|  |  |  |  |  |  | F10-4MR | -1166.52 |
|  |  |  |  |  |  | F1-4MR | -1166.52 |
| **Table S17**. Structural energies of Al distribution and corresponding Na^+^ positions on Y-zeolites  (Si/Al = 3.4, Si = 37, Al = 11) | | | | | | | |
| Based on  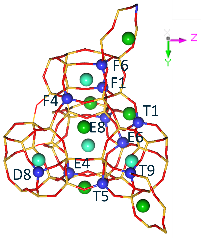  **D8-D6R** from Table S16  The first ten sequences of Al:  T1-6MR→E8-6MR→T5-D6R→E4-6MR→E6-D6R→T9-6MR→F4-6MR→F1-D6R→F6-6MR→D8-D6R | | | | | | | |
| T zone | Energy (eV) | E zone | Energy (eV) | D zone | Energy (eV) | F zone | Energy (eV) |
| T2-6MR | -1169.15 | E2-4MR middle | -1169.12 | **D4-6MR** | **-1169.59** | F9-6MR | -1169.10 |
| T7-6MR | -1169.01 | E2-6MR | -1169.02 | D5-6MR | -1169.53 | F9-4MR | -1168.94 |
| T2-4MR | -1168.97 | E2-4MR | -1168.91 | D11-6MR | -1169.53 | F8-4MR | -1168.94 |
| T2-4MR middle | -1168.88 |  |  | D10-6MR | -1169.31 |  |  |
| T7-4MR | -1168.86 |  |  | D4-4MR | -1169.22 |  |  |
| T7-4MR middle | -1168.86 |  |  | D10-4MR | -1169.21 |  |  |
|  |  |  |  | D4-4MR middle | -1169.15 |  |  |
|  |  |  |  | D5-4MR | -1169.14 |  |  |
|  |  |  |  | D11-4MR | -1169.13 |  |  |
|  |  |  |  | D12-4MR | -1169.08 |  |  |
| **Table S17-1**. Structural energies of Al distribution and corresponding Na^+^ positions on Y-zeolites  (Si/Al = 3.4, Si = 37, Al = 11) | | | | | | | |
| Based on  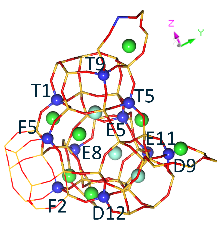  **F2-6MR** from Table S16-1  The first ten sequences of Al:  T1-6MR→E8-6MR→T5-D6R→E11-D6R→T9-6MR→E5-6MR→D9-6MR→D12-D6R→F5-6MR→F2-6MR | | | | | | | |
| T zone | Energy (eV) | E zone | Energy (eV) | D zone | Energy (eV) | F zone | Energy (eV) |
| T2-6MR | -1169.20 | E3-D6R | -1169.18 | D1-4MR | -1169.02 | **F8-D6R** | **-1169.21** |
| T7-4MR | -1168.81 | E3-4MR | -1168.79 | D1-4MR middle | -1168.95 | F10-D6R | -1169.03 |
| T7-6MR | -1168.73 | E3-4MR middle | -1168.62 | D4-4MR | -1168.94 | F10-6MR | -1168.96 |
| T7-4MR middle | -1168.66 |  |  | D1-4MR middle up | -1168.92 | F8-4MR middle | -1168.80 |
| T2-4MR | -1168.65 |  |  | D6-4MR | -1168.85 | F8-4MR | -1168.71 |
| T2-4MR middle | -1168.46 |  |  | D6-4MR middle | -1168.84 | F10-4MR middle | -1168.64 |
|  |  |  |  | D3-4MR | -1168.81 | F10-4MR | -1168.57 |
|  |  |  |  | D3-4MR middle | -1168.75 | F10-4MR middle | -1168.37 |
| **Table S18**. Structural energies of Al distribution and corresponding Na^+^ positions on Y-zeolites  (Si/Al = 3, Si = 36, Al = 12) | | | | | | | |
| Based on  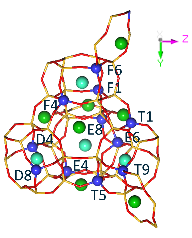  **D4-6MR** from Table S17  The first eleven sequences of Al:  T1-6MR→E8-6MR→T5-D6R→E4-6MR→E6-D6R→T9-6MR→F4-6MR→F1-D6R→F6-6MR→D8-D6R→D4-6MR | | | | | | | |
| T zone | Energy (eV) | E zone | Energy (eV) | D zone | Energy (eV) | F zone | Energy (eV) |
| T2-6MR | -1171.49 | E2-4MR middle | -1171.63 | **D10-6MR** | **-1171.57** | **F9-6MR** | **-1171.80** |
| T7-6MR | -1171.48 | E2-4MR | -1171.33 | D10-4MR | -1171.44 | F9-4MR middle | -1171.44 |
| T2-4MR | -1171.32 |  |  | D12-4MR | -1171.33 | F8-4MR | -1171.32 |
| T2-4MR middle | -1171.18 |  |  | D12-4MR middle | -1171.31 | F9-4MR | -1171.31 |
| T7-4MR middle | -1171.14 |  |  | D10-4MR middle | -1171.14 | F8-4MR middle | -1171.21 |
| T7-4MR | -1171.10 |  |  |  |  |  |  |
| **Table S18-1**. Structural energies of Al distribution and corresponding Na^+^ positions on Y-zeolites  (Si/Al = 3, Si = 36, Al = 12) | | | | | | | |
| Based on  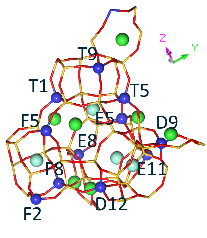  **F8-D6R** from Table S17-1  The first eleven sequences of Al:  T1-6MR→E8-6MR→T5-D6R→E11-6MR→T9-6MR→E5-6MR→D9-6MR→D12-D6R→F5-6MR→F2-6MR→F8-D6R | | | | | | | |
| T zone | Energy (eV) | E zone | Energy (eV) | D zone | Energy (eV) | F zone | Energy (eV) |
| T2-6MR | -1171.23 | E3-D6R | -1171.16 | D1-4MR | -1171.34 | F10-6MR | -1171.29 |
| T7-4MR middle | -1170.85 | E3-4MR | -1170.94 | D1-4MR middle | -1171.30 | F10-4MR middle | -1170.98 |
| T7-6MR | -1170.84 | E3-4MR middle | -1170.92 | D1-4MR middle | -1171.30 | F10-4MR | -1170.76 |
| T2-4MR | -1170.66 |  |  | D6-4MR middle | -1171.27 | F10-4MR middle | -1170.72 |
| T2-4MR middle | -1170.66 |  |  | D6-4MR | -1171.20 |  |  |
|  |  |  |  | D4-4MR | -1171.14 |  |  |
| **Table S19**. Structural energies of Al distribution and corresponding Na^+^ positions on Y-zeolites  (Si/Al = 2.7, Si = 35, Al = 13) | | | | | | | |
| Based on  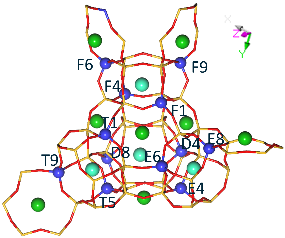  **F9-6MR** from Table S18  The first twelve sequences of Al:  T1-6MR→E8-6MR→T5-D6R→E4-6MR→E6-D6R→T9-6MR→F4-6MR→F1-D6R→F6-6MR→D8-D6R→D4-6MR→F9-6MR | | | | | | | |
| T zone | Energy (eV) | E zone | Energy (eV) | D zone | Energy (eV) | F zone | Energy (eV) |
| T2-4MR | -1173.55 | E2-4MR middle | -1173.69 | **D10-4MR** | **-1173.92** |  |  |
| T2-4MR middle | -1173.45 | E2-4MR | -1173.62 | D12-4MR | -1173.66 |  |  |
|  |  |  |  | D10-4MR middle | -1173.56 |  |  |
|  |  |  |  | D12-4MR middle | -1173.50 |  |  |
| **Table S19-1**. Structural energies of Al distribution and corresponding Na^+^ positions on Y-zeolites  (Si/Al = 2.7, Si = 35, Al = 13) | | | | | | | |
| Based on  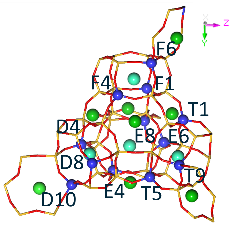  **D10-6MR** from Table S18  The first twelve sequences of Al:  T1-6MR→E8-6MR→T5-D6R→E4-6MR→E6-D6R→T9-6MR→F4-6MR→F1-D6R→F6-6MR→D8-D6R→D4-6MR→D10-6MR | | | | | | | |
| T zone | Energy (eV) | E zone | Energy (eV) | D zone | Energy (eV) | F zone | Energy (eV) |
| T2-4MR | -1173.53 | E2-4MR middle | -1173.58 | D12-4MR middle | -1173.26 | **F9-4MR middle** | **-1173.65** |
| T2-4MR middle | -1173.44 | E2-4MR | -1173.21 | D12-4MR | -1173.24 | F8-4MR | -1173.35 |
| T7-4MR middle | -1173.44 |  |  |  |  | F9-4MR | -1173.32 |
| T7-4MR | -1173.31 |  |  |  |  | F8-4MR middle | -1173.27 |
| **Table S20**. Structural energies of Al distribution and corresponding Na^+^ positions on Y-zeolites  (Si/Al = 2.4, Si = 34, Al = 14) | | | | | | | |
| Based on  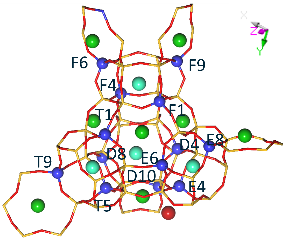  **D10-4MR** from Table S19  The first thirteen sequences of Al:  T1-6MR→E8-6MR→T5-D6R→E4-6MR→E6-D6R→T9-6MR→F4-6MR→F1-D6R→F6-6MR→D8-D6R→D4-6MR→F9-6MR→D10-4MR | | | | | | | |
| T zone | Energy (eV) | E zone | Energy (eV) | D zone | Energy (eV) | F zone | Energy (eV) |
| T2-4MR | -1175.65 | **E2-4MR** | **-1175.71** | D12-4MR | -1175.65 |  |  |
| T2-4MR middle | -1175.55 | E2-4MR middle | -1175.69 | D12-4MR middle | -1175.63 |  |  |
| **Table S20-1**. Structural energies of Al distribution and corresponding Na^+^ positions on Y-zeolites  (Si/Al = 2.4, Si = 34, Al = 14) | | | | | | | |
| Based  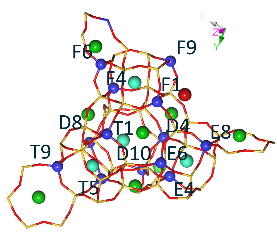  on **F9-4MR** from Table S19-1  The first thirteen sequences of Al:  T1-6MR→E8-6MR→T5-D6R→E4-6MR→E6-D6R→T9-6MR→F4-6MR→F1-D6R→F6-6MR→D8-D6R→D4-6MR→D10-6MR→F9-4MR | | | | | | | |
| T zone | Energy (eV) | E zone | Energy (eV) | D zone | Energy (eV) | F zone | Energy (eV) |
| T2-4MR | -1175.46 | **E2-4MR** | **-1175.65** | D12-4MR | -1175.60 |  |  |
| T2-4MR middle | -1175.35 | E2-4MR middle | -1174.83 | D12-4MR middle | -1175.57 |  |  |

**Table S21**. Energy difference of Na^+^ positions with increasing Al atoms.

| 14Al sequence: T1-6MR→E8-6MR→T5-D6R→E4-6MR→E6-D6R→T9-6MR→F4-6MR→F1-D6R→F6-6MR→D8-D6R→D4-6MR→F9-6MR→D10-6MR→E2-4MR | | | |
| --- | --- | --- | --- |
| Na^+^ Positions  *∆E* (eV)  Al number | 6MR | 4MR | D6R |
| *1Al:* T1 | -0.20 | 0.00 | 0.04 |
| *2Al:* E8 | -0.58 | 0.00 | -0.13 |
| *3Al:* T5 | -0.24 | 0.00 | -0.56 |
| *4Al:* E4 | -0.60 | 0.00 | -0.17 |
| *5Al:* E6 | - | 0.00 | - |
| *6Al:* T9 | -0.35 | 0.00 | - |
| *7Al:* F4 | -0.59 | 0.00 | -0.16 |
| *8Al:* F1 | - | 0.00 | - |
| *9Al:* F6 | -0.40 | 0.00 | - |
| *10Al:* D8 | - | 0.00 | - |
| *11Al:* D4 | -0.37 | 0.00 | - |
| *12Al:* F9 | -0.13 | 0.00 | - |
| *13Al:* D10 | - | 0.00 | - |
| *14Al:* E2 | - | 0.00 | - |

**Table S22**. Energy difference of one NH_4_^+^ positions on Na-Y1 with different Si/Al.

| Al number | NH_4_^+^ Positions | Energy (eV) | *∆E* (eV) |
| --- | --- | --- | --- |
| 1NH_4_-Y1  with 1Al atom | T1-6MR | -1168.03 | 0.07 |
|  | T1-4MR | -1168.10 | 0.00 |
| 2Na+1NH_4_-Y1  with 3Al atoms | E8-6MR | -1172.42 | -0.42 |
|  | E8-4MR | -1172.00 | 0.00 |
|  | T5D6R-6MR | -1172.09 | 0.05 |
|  | T5D6R-4MR | -1172.14 | 0.00 |
|  | T1-6MR | -1172.41 | -0.20 |
|  | T1-4MR | -1172.22 | 0.00 |
| 6Na+1NH_4_-Y1  with 7Al atoms | F4-6MR | -1181.42 | -0.30 |
|  | F4-4MR | -1181.12 | 0.00 |
|  | T1-6MR | -1181.38 | -0.50 |
|  | T1-4MR | -1180.87 | 0.00 |
|  | T9-6MR | -1181.37 | -0.37 |
|  | T9-4MR | -1181.00 | 0.00 |
|  | E8-6MR | -1181.33 | -0.21 |
|  | E8-4MR | -1181.12 | 0.00 |
|  | E4-6MR | -1181.33 | -0.21 |
|  | E4-4MR | -1180.90 | 0.00 |
| 13Na+1NH_4_-Y1  with 14Al atoms | F4-6MR | -1196.43 | -0.48 |
|  | F4-4MR | -1195.95 | 0.00 |
|  | D4-6MR | -1196.40 | -0.33 |
|  | D4-4MR | -1196.06 | 0.00 |
|  | F9-6MR | -1196.36 | -1.04 |
|  | F9-4MR | -1195.33 | 0.00 |
|  | E8-6MR | -1196.35 | -0.21 |
|  | E8-4MR | -1196.14 | 0.00 |
|  | T1-6MR | -1196.35 | -0.90 |
|  | T1-4MR | -1195.45 | 0.00 |
|  | T9-6MR | -1196.30 | -0.34 |
|  | T9-4MR | -1195.96 | 0.00 |
|  | E4-6MR | -1196.18 | -0.44 |
|  | E4-4MR | -1195.74 | 0.00 |
|  | F6-6MR | -1196.18 | -0.61 |
|  | F6-4MR | -1195.57 | 0.00 |

**Table S23**. Energy difference of one NH_4_^+^ positions on Y1 (**4Na+9H+1NH_4_**).

| Al number | NH_4_^+^ Positions | Energy (eV) | *∆E* (eV) |
| --- | --- | --- | --- |
| 4Na+9H+1NH_4_-Y1  with 14Al atoms | F4-6MR | -1193.39 | 0.02 |
|  | F4-4MR | -1193.41 | 0.00 |
|  | D4-6MR | -1193.48 | -0.08 |
|  | D4-4MR | -1193.40 | 0.00 |
|  | F9-6MR | -1193.12 | 0.10 |
|  | F9-4MR | -1193.22 | 0.00 |
|  | E8-6MR | -1192.29 | 0.75 |
|  | E8-4MR | -1193.04 | 0.00 |
|  | T1-6MR | -1194.22 | -0.57 |
|  | T1-4MR | -1193.64 | 0.00 |
|  | T9-6MR | -1192.97 | 0.28 |
|  | T9-4MR | -1193.25 | 0.00 |
|  | E4-6MR | -1194.11 | -0.89 |
|  | E4-4MR | -1193.22 | 0.00 |
|  | F6-6MR | -1194.05 | -0.68 |
|  | F6-4MR | -1193.38 | 0.00 |

**Table S24**. Sequential substitution enthalpy (ZPE calculation was performed with released Na^+^ ions and framework O atoms, and fixed framework Al and Si atoms).

|  | Site | E_tot_ (eV) | ZPE (eV) | ∆∆*H* |
| --- | --- | --- | --- | --- |
| 0 Al | - | -1145.50 | 10.12 | - |
| 1st Al | T1-6MR | -1147.22 | 10.06 | -0.91 |
| 1st Al | T1-4MR | -1147.05 | 10.11 | -0.69 |
| 1st Al | T1-D6R | -1147.11 | 10.09 | -0.77 |
| 2nd Al | E8-6MR | -1149.47 | 10.20 | -1.24 |
| 3th Al | T5-D6r | -1151.70 | 10.21 | -1.34 |
| 4th Al | E4-6MR | -1154.00 | 10.27 | -1.37 |
| 5th Al | E6-D6r | -1156.34 | 10.18 | -1.56 |
| 6th Al | T9-6MR | -1158.44 | 10.23 | -1.19 |
| 7th Al | F4-6MR | -1160.69 | 10.18 | -1.42 |
| 8th Al | F1-D6r | -1162.79 | 10.23 | -1.18 |
| 9th Al | F6-6MR | -1165.05 | 10.26 | -1.36 |
| 10th Al | D8-D6r | -1167.20 | 10.25 | -1.29 |
| 11th Al | D4-6MR | -1169.59 | 10.20 | -1.57 |
| 12th Al | F9-6MR | -1171.80 | 10.23 | -1.30 |
| 13th Al | D10-4MR | -1173.92 | 10.11 | -1.38 |
| 14th Al | E2-4MR | -1175.71 | 10.20 | -0.83 |

**References**

[S1] J. P. Perdew, K. Burke, M. Ernzerhof, “Generalized Gradient Approximation Made Simple” *Phys. Rev. Lett.* **1996**, 77, 3865-3868.

[S2] J. P. Perdew, Y. Wang, “Accurate and simple analytic representation of the electron-gas correlation energy” *Phys. Rev. B.* **1992**, 45, 13244-13249.

[S3] S. Grimme, J. Antony, S. Ehrlich, H. Krieg, “A consistent and accurate ab initio parametrization of density functional dispersion correction (DFT-D) for the 94 elements H-Pu” *J. Chem. Phys.* **2010**, 132, 154104.

[S4] H. J. Monkhorst, J. D. Pack, “Special points for Brillouin-zone integrations” *Phys. Rev. B.* **1976**, 13, 5188-5192.

[S5] H. C. Andersen, “Molecular dynamics simulations at constant pressure and/or temperature” *J. Chem. Phys.* **1980**, 72, 2384-2393.

[S6] P. A. Redhead, “Thermal desorption of gases” *Vacuum.* **1962**, 12, 203-211.

[S7] A. P. Bartók, R. Kondor, G. Csányi, “On representing chemical environments” *Phys. Rev. B.* **2013**, 87, 184115.

[S8] S. De, A. P. Bartok, G. Csanyi, “Comparing molecules and solids across structural and alchemical space” *Phys. Chem. Chem. Phys.* **2016**, 18, 13754-13769.

[S9] L. Himanen, M. O.J. Jäger, E. V. Morooka, F. F. Canova, Y. S. Ranawat, D. Z. Gao, P. Rinke, A. S. Foster, “DScribe: Library of descriptors for machine learning in materials science” *Comput. Phys. Commun.* **2020**, 247, 106949.

[S10] K. Pearson, “LIII. On lines and planes of closest fit to systems of points in space” *The London, Edinburgh, and Dublin Philosophical Magazine and Journal of Science* **1901**, 2, 559-572 (1901).

[S11] J. Behler, “Atom-centered symmetry functions for constructing high-dimensional neural network potentials” *J. Chem. Phys.* **2011**, 134, 074106.

[S12] T. Chen, T. He, “Xgboost: extreme gradient boosting” *R Package version 0.4-2*, **2015**.

[S13] L. Breiman, “Random Forests” *Machine Learning* **2001**, 45, 5-32.

[S14] G. Ke, Q. Meng, T. Finley, T. Wang, W. Chen, W. Ma, Q. Ye, T. Liu, “Lightgbm: A highly efficient gradient boosting decision tree” *Advances in neural information processing systems.* **2017**, 30.

[S15] F. Pedregosa, G. Varoquaux, A. Gramfort, V. Michel, B. Thirion, O. Grisel, M. Blondel, P. Prettenhofer, R. Weiss, V. Dubourg**,** J. Vanderplas, A. Passos, D. Cournapeau, M. Brucher, M. Perrot, É. Duchesnay, “Scikit-learn: Machine Learning in Python” *J. Mach. learn. Res.* **2011**, 12, 2825-2830.

[S16] X. Peng, R. Pan, X. Li, W. Zhong, F. Qian, “Molecular descriptor-assisted interpretable machine learning: A scheme for guiding the synthesis of zeolites with target structures” *Chemical Engineering Science* **2025**, 308, 121378.

[S17] K. Wolinski, J. F. Hinton, P. Pulay, "Efficient implementation of the gauge-independent atomic orbital method for NMR chemical shift calculations" *J. Am. Chem. Soc.* **1990**, 112, 8251-8260.

[S18] M. J. Frisch, G. W. Trucks, H. B. Schlegel, G. E. Scuseria, M. A. Robb, J. R. Cheeseman, G. Scalmani, V. Barone, G. A. Petersson, H. Nakatsuji, X. Li, M. Caricato, A. V. Marenich, J. Bloino, B. G. Janesko, R. Gomperts, B. Mennucci, H. P. Hratchian, J. V. Ortiz, A. F. Izmaylov, J. L. Sonnenberg, D. Williams-Young, F. Ding, F. Lipparini, F. Egidi, J. Goings, B. Peng, A. Petrone, T. Henderson, D. Ranasinghe, V. G. Zakrzewski, J. Gao, N. Rega, G. Zheng, W. Liang, M. Hada, M. Ehara, K. Toyota, R. Fukuda, J. Hasegawa, M. Ishida, T. Nakajima, Y. Honda, O. Kitao, H. Nakai, T. Vreven, K. Throssell, J. A. Montgomery, Jr., J. E. Peralta, F. Ogliaro, M. J. Bearpark, J. J. Heyd, E. N. Brothers, K. N. Kudin, V. N. Staroverov, T. A. Keith, R. Kobayashi, J. Normand, K. Raghavachari, A. P. Rendell, J. C. Burant, S. S. Iyengar, J. Tomasi, M. Cossi, J. M. Millam, M. Klene, C. Adamo, R. Cammi, J. W. Ochterski, R. L. Martin, K. Morokuma, O. Farkas, J. B. Foresman, and D. J. Fox, Gaussian 16, Revision C.01, Gaussian, Inc., Wallingford CT, **2016**.

[S19] C. Lee, W. Yang, R. G. Parr, “Development of the Colle-Salvetti correlation-energy formula into a functional of the electron density” *Phys. Rev. B.* **1988**, 37, 785-789.

[S20] A. D. Becke, “Density-functional thermochemistry. III. The role of exact exchange” *J. Chem. Phys.* **1993**, 98, 5648-5652.

[S21] A. D. Becke, “A new mixing of Hartree-Fock and local density-functional theories” *J. Chem. Phys.* **1993**, 98, 1372-1377.

[S22] F. Jensen, “Basis Set Convergence of Nuclear Magnetic Shielding Constants Calculated by Density Functional Methods” *J. Chem.* *Theory Comput.* **2008**, 4, 719-727.

[S23] C. Liu, G. Li, E. J. M. Hensen, E. A. Pidko, “Nature and catalytic role of extraframework aluminum in faujasite zeolite: a theoretical perspective” *ACS Catal.* **2015**, 5, 7024-7033.

[S24] Z. Zhao, Y. Xing, S. Li, X. Meng, F. Xiao, R. McGuire, A. N. Parvulescu, U. Müller, W. Zhang, “Mapping Al Distributions in SSZ-13 Zeolites from ^23^Na Solid-State NMR Spectroscopy and DFT Calculations” *J. Phys. Chem. C.* **2018**, 122, 9973-9979.
